# A Roadmap for Extreme-Mechanics Hydrogels: From Toughening Mechanisms to Intelligent System Integration

**DOI:** 10.1007/s40820-026-02179-8

**Published:** 2026-04-13

**Authors:** Aixin Tong, Zhiyu Huang, Annan He, Sijie Qiao, Zhicheng Shi, Xiaotian Wang, Zhen Chen, Peiying Hu, Kai Wang, Jin Qian, Weilin Xu, Fengxiang Chen

**Affiliations:** 1 State Key Laboratory of New Textile Materials and Advanced Processing, Hubei Key Laboratory of Digital Textile Equipment, Wuhan Textile University, Wuhan, 430073 People’s Republic of China; 2 Huanjiang Laboratory, Zhuji, 311800 People’s Republic of China; 3 School of Aeronautics and Astronautics, Zhejiang University, Hangzhou, 310027 People’s Republic of China

**Keywords:** Extreme-mechanics, Hydrogels, Mechanisms, Synthesis, Applications

## Abstract

Hydrogels, as water-rich, three-dimensional polymer networks, have emerged as essential materials across diverse fields including bio-integrated electronics, load-bearing biomedical implants, and soft robotics. However, conventional hydrogels often fail under mechanical extremes, limiting their deployment in mechanically demanding environments. This review bridges this gap by presenting a transformative design paradigm that shifts the focus from merely robust hydrogels to intelligently adaptive systems capable of withstanding and dynamically responding to extreme mechanical environments. We first deconstruct the fundamental toughening mechanisms—including sacrificial bonding, topological entanglements, and nanocomposite reinforcement—that form the foundation of mechanical robustness. Moving beyond static strength, we critically examine how stimuli-responsive elements (e.g., temperature, light, pH, magnetic fields) can be integrated to enable real-time, dynamic modulation of mechanical properties. Advanced fabrication strategies, particularly bioinspired structuring and 3D printing, are highlighted as essential tools for achieving hierarchical architectures that optimize stress distribution and functional integration. Finally, we showcase pioneering applications in artificial muscles, wearable sensors, and adaptive tissue scaffolds, culminating in a forward-looking perspective on the convergence of artificial intelligence, multiscale modeling, and self-growing materials to guide the development of next-generation autonomous hydrogel systems.

## Highlights

- Scenario-adaptive design principles for extreme-mechanics hydrogels, connecting application-specific mechanical requirements with multiscale material and structural design.
- Mechanistic integration of extreme mechanical performance and multi-environmental responsiveness, enabled by dynamic networks, nanocomposite hybridization, and biomimetic hardsoft architectures.
- System-level perspective on hydrogels as active components in intelligent systems, emphasizing multiscale structuring and closed-loop “sense–think–act” functionality.

## Graphical abstract

**Figure:**
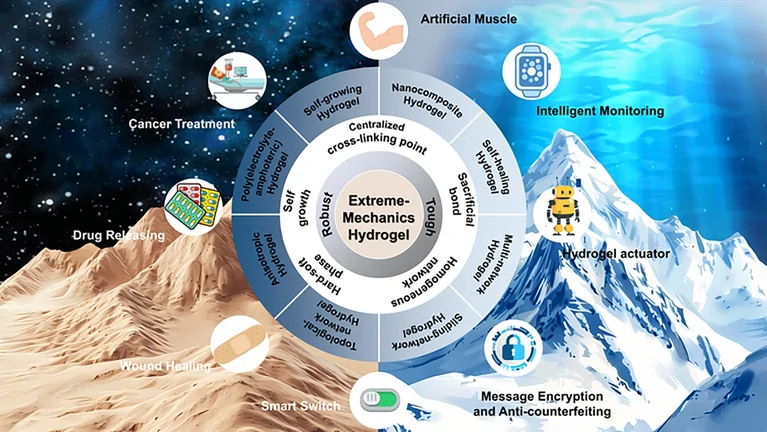

## Introduction

**Fig. 1:**
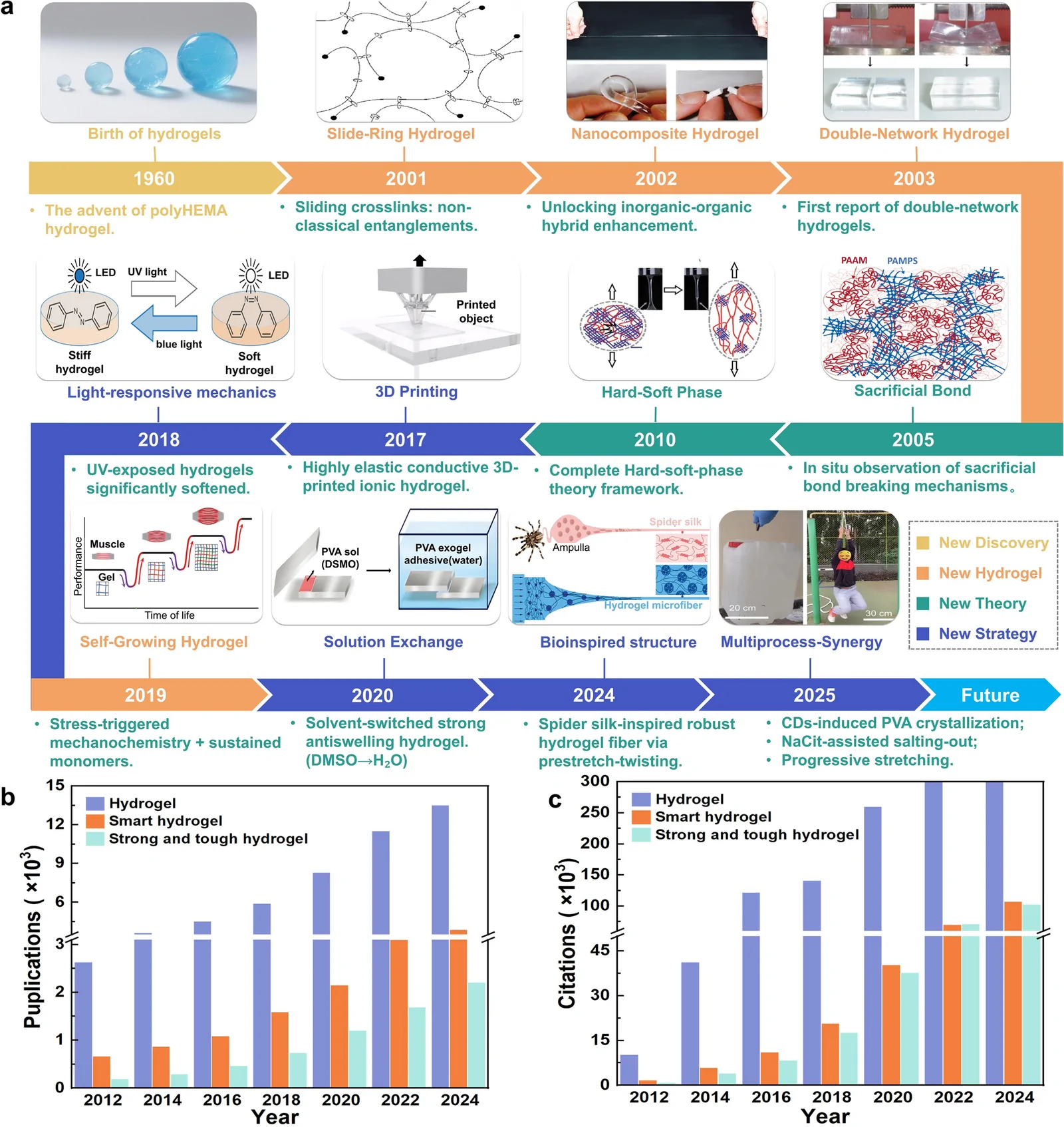
**a** Historical evolution of hydrogel research highlighting the development of intelligent functionalities, mechanical performance, and associated enhancement strategies. **b** Publication trends in hydrogel subfields (2011–2024) from WOS Core Collection, showing exponential growth in mechanical resilience investigations. **c** Citation evolution of hydrogel categories (2011–2024) via WOS, confirming paradigm shift toward mechanically enhanced systems. Reproduced with permission from [20–30]

In 1952, Otto Wichterle and Drahoslav Lim successfully synthesized the world’s first hydrophilic polymer material–polyhydroxyethyl methacrylate (PHEMA), and based on this, they developed the first batch of soft contact lenses [1]. This pioneering achievement marked the beginning of research on hydrogel materials. Hydrogels, due to their high-water content and three-dimensional crosslinked polymer network structure, possess good permeability and processability while maintaining flexibility and tissue compatibility. They have become a key material with high adaptability and potential for multiple functions. Since Tanaka et al. [2] first reported their volumetric phase transition behavior, significant efforts have been devoted to developing smart hydrogels capable of responding to diverse external stimuli. These stimulus-responsive systems have enabled dynamic modulation of hydrogel structure, morphology, and functionality, opening new avenues in applications such as artificial muscles [3], intelligent monitoring [4], soft actuators [5], information encryption [6], smart switches [7], wound healing [8], targeted drug delivery [9], and smart adaptive stealth [10]. Driven by this multifunctionality, the field has rapidly progressed from single-stimulus to multi-responsive hydrogels [11]. Parallel advancements in conductive hydrogels [12], self-healing materials, and self-growing systems [13] have further enriched their application potential in next-generation bioelectronics and adaptive systems. However, despite these sophisticated functional attributes, traditional hydrogels remain mechanically vulnerable, exhibiting low fracture stress, limited extensibility, and poor fatigue resistance. Most conventional hydrogels display fracture stresses below 1 MPa, stretchability under 100%, and fracture energy < 10 J m^−2^ [14, 15], rendering them unsuitable for mechanically demanding environments. Emerging applications demand hydrogels with extreme mechanical resilience, where toughness, strength, and fatigue resistance must rival or surpass natural tissues. For example, artificial muscles must simultaneously exhibit high toughness, rapid responsiveness, and durability under repeated strain [16]. Smart wearables and textile sensors require flexibility, large deformation tolerance, and long-term cyclic stability [4]. Hydrogel-based robotic grippers must endure repetitive gripping forces without structural failure [17]. In addition, hydrogel implants may be subjected to excessive stretching, stress fatigue, and internal microcracks caused by the frequent and continuous movements of human organs and joints (such as the continuous contraction and expansion of the heart or blood vessels), which poses a challenge for the application of hydrogel electronics. In contrast, biological cartilage withstands over one million compressive cycles annually at 4–9 MPa stress levels, with fracture energy exceeding 1000 J m^−2^ [18], setting a mechanical benchmark that synthetic hydrogels must approach or exceed. These escalating mechanical demands have catalyzed the development of diverse toughening theories and structural design strategies aimed at engineering hydrogels with resilience comparable to natural load-bearing tissues (Fig. 1a).

Despite the rapid evolution of smart hydrogel technologies, the development of hydrogels with both high strength and toughness, capable of performing reliably in mechanically extreme environments, remains relatively underexplored. As illustrated in Fig. 1b, the number of publications specifically addressing strong and tough hydrogels between 2010 and 2024 remains modest compared to the broader surge in hydrogel research. This disparity highlights a critical research gap: While hydrogel functionalities such as stimuli responsiveness, conductivity, and bioactivity have seen widespread attention, the mechanical durability required for demanding applications is still in its early stages of systematic investigation. Driven by the increasing demand for high-performance intelligent systems, researches are now shifting focus toward the design of intelligent hydrogels with enhanced mechanical robustness, i.e., integrating toughness, stretchability, and fatigue resistance into multifunctional frameworks. Concurrently, a growing body of work is investigating how external environmental stimuli [19] such as temperature, pH, light, and magnetic fields can dynamically modulate hydrogel structure and mechanical behavior, enabling adaptive performance in complex environments (Fig. 1c).

Building upon groundbreaking prior research (e.g., Gong’s comprehensive treatise on design principles for robust hydrogels [31], Nika Petelinšek and Stefan Mommer’s seminal work on load-bearing hydrogel solution [32], and Stephanie Fuchs, Kaavian Shariati, and Minglin Ma’s contributions to biomedical applications of specialty tough hydrogels [33]), this review bridges critical gaps by systematically analyzing polymer network structures and fabrication strategies for mechanically extreme hydrogels. We first outline five core toughening theories—nanocomposite interface strengthening, dynamic reconstruction of sacrificial bonds, topological optimization of homogeneous networks, biomimetic configurations of hard/soft phases, and self-growing environmental adaptability, revealing how each governs mechanical characteristics (e.g., fracture toughness, resilience, strain tolerance). Next, we categorize hydrogel systems (double-network, nanocomposite, slide-ring) by structural principles and performance metrics, then review three key fabrication pathways: biomimetic design, microphase separation, and advanced 3D printing, emphasizing their role in multiscale mechanical optimization. We further discuss harnessing environmental stimuli to actively regulate mechanical behavior for smart-responsive systems. Finally, we introduce a scenario-adaptive design framework that prioritizes mechanical properties according to specific operational environments, such as marine, wearable, aerospace, and biomedical settings, thereby providing a guided roadmap for material selection. We then highlight breakthrough applications that fulfill these scenario-specific mechanical demands, spanning soft robotics, wearable electronics, and bio-integrated devices, thereby demonstrating the cross-disciplinary potential of extreme-mechanics hydrogels, which refer to systems engineered to reliably withstand demanding conditions including tensile or compressive strength exceeding 1 MPa, fracture energy over 1000 J m^−2^, elongation greater than 500%, or high-cycle fatigue loading. By elucidating the interplay between polymer architecture, processing, and performance, this review provides a comprehensive roadmap for designing next-generation hydrogels that withstand mechanical extremes while delivering intelligent functionality. Following the conceptual framework outlined by Zheng et al. [34], we regard the ultimate objective as intelligent system integration. This entails the construction of intelligent hydrogel systems with a “sensing–decision–actuation” closed-loop capability. This entails the functional coupling of advanced hydrogels with external electronic, optical, or mechanical modules to form adaptive devices capable of autonomous perception, feedback, and actuation. By elucidating the interplay between polymer architecture, processing, and performance, this review aims to bridge the gap from fundamental toughening mechanisms to such sophisticated system-level implementations.

## Toughening Mechanisms for Mechanically Extreme Hydrogels

The rational design of hydrogels with exceptional mechanical performance has become a critical focus in materials science. Achieving this goal requires guidance from fundamental toughening theories that inform the structural optimization and functional enhancement of hydrogel networks. Among these, five representative frameworks, including concentrated crosslinking point theory, sacrificial bond theory, homogeneous network theory, hard–soft phase structure theory, and self-growth theory, have provided essential paradigms for engineering mechanically robust and responsive hydrogels.

**Fig. 2:**
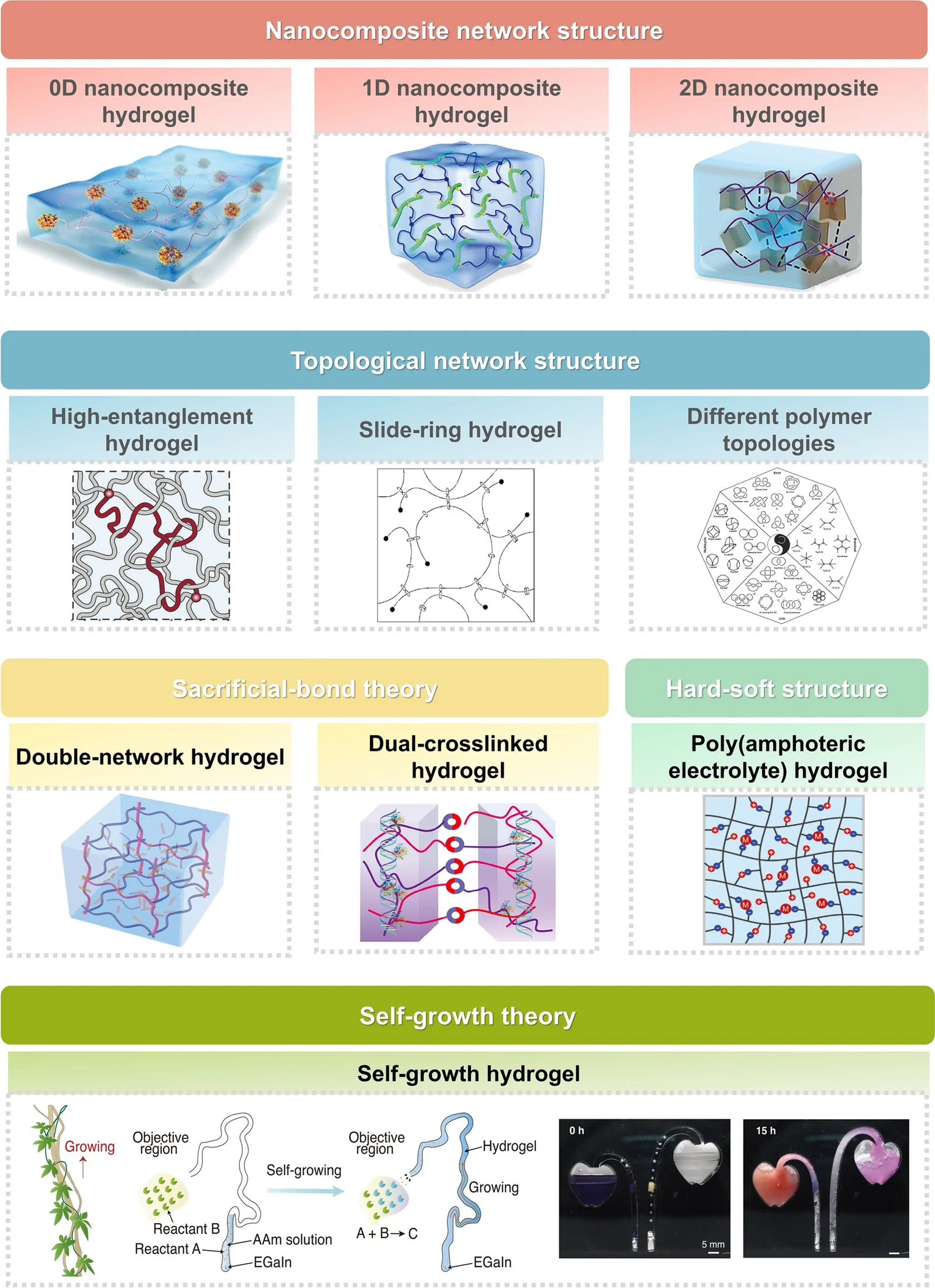
Overview of key hydrogel toughening theories, including concentrated crosslinking, sacrificial bonding, homogeneous networks, hard–soft phase structuring, and self-growth, and the representative hydrogel types they guide, such as nanocomposite, double-network, slide-ring, high-entanglement, and self-growing systems. Reproduced with permission from [20, 35–43]

These theories have led to the development of various hydrogel systems, including nanocomposite (NC) hydrogels, double-network (DN) hydrogels, slide-ring (SR) hydrogels, high-entanglement (HE) hydrogels, self-growth (SG) hydrogels, and poly(amphoteric electrolyte) (PA) hydrogels (Fig. 2). Each system embodies distinct molecular strategies to achieve extreme mechanical properties, such as high fracture toughness, large stretchability, and environmental adaptability under cyclic or high-load conditions (Tables 1, 2). In practice, these theories are often used synergistically: Concentrated crosslinking and homogeneous network strategies enhance structural strength and dimensional stability; sacrificial bonds and hard–soft phase configurations improve toughness, elasticity, and energy dissipation; and self-growth theory introduces dynamic remodeling and repairability. This section reviews the five major toughening theories and their corresponding hydrogel systems, providing a theoretical foundation for the development of next-generation hydrogels engineered for mechanical extremes.

**Table 1:** Comparison of toughening mechanisms and effects by nanofiller dimensionality

Comparison aspect	0D nanomaterials	1D nanomaterials	2D nanomaterials
Primary toughening mechanism	Act as multipoint, isotropic crosslinking sites, dissipating energy through interfacial debonding and friction	Act as fiber-like bridging agents, dissipating energy via pull-out, orientation, and crack deflection	Act as sheet-like barriers, dissipating energy through large-scale crack deflection/branching and interfacial sliding
Stress transfer characteristics	Uniform yet localized, primarily enhancing stiffness and strength	Highly anisotropic, enabling efficient axial load transfer, significantly improving tensile strength and toughness	Efficient in-plane, transferring, and dispersing stress via an ultra-large interface, markedly enhancing fracture toughness and tear resistance
Regulation of crack behavior	Restrict microcrack initiation but offer limited hindrance to macroscopic crack propagation	Effectively bridge cracks, substantially prolonging the propagation path	Act as the most potent barrier, forcing cracks into large-angle deflection and circumvention, thereby maximizing energy consumption
Key mechanical performance	Compressive modulus, hardness, isotropic reinforcement	Tensile strength, fracture energy, fatigue resistance (especially along the orientation direction)	Fracture toughness, tear strength, impact resistance, gas/solvent barrier properties
Typical functional roles	Multifunctional nodes (conductivity, magnetism, fluorescence, catalysis)	Conductive pathways, thermal management channels, directional reinforcement units	Flexible electrodes, barrier layers, photothermal conversion films, anisotropic actuators

**Table 2:** The design strategies, mechanical properties, and functional applications of strong and tough hydrogels

Strategy		Component		Mechanical properties	Applications	References
Nanocomposite	0D nanomaterials	Si NPs	PNIPAm@PAM	Tensile strength: 0.5 MPa; Tensile strain: 2800%; Compressive strength: 36 MPa (compressive strain 99.7%)	Hydrogel actuator, soft robotics, human-friendly wearable devices, and biomedical devices (Thermal-responsive)	[44]
		Fe_3_O_4_ NPs@CNCs	PAM/PAAc	Tensile strength: 28.9 MPa; Young’s modulus: 83 MPa; Toughness: 16.96 MJ m^−3^. Rapid bending velocity (3.2°min^−1^ .mm^−1^) in response to solvents	Hydrogel fiber actuator, artificial muscles, smart textiles, and soft robotics (Magnetically responsive)	[45]
		Au-Se NPs	PAM	Tensile strength: 2.48 MPa; Tensile strain: 1970%; Toughness: 9.95 MJ m^−3^	Smart drug release, biomedical and engineering fields. (Photothermal-responsive)	[46]
		Lig-Ag NPs	PVA	Tensile strength: 0.79 MPa; Tensile strain: 315%; Compressive strength: 210.75 kPa (compressive strain 60%); Young’s modulus: 2.26 kPa; Toughness: 1.04 MJ m^−3^. Fatigue resistance: 40% compressive strain for 100 cycles without resting intervals	Piezoresistive pressure sensors (Conductive)	[47]
		SiO_2_@PANI	PAM/DMx	Tensile strength: 307 kPa; Tensile strain: 1523%; Young’s modulus: 155 kPa; Toughness: 3.24 MJ m^−3^; Fatigue resistance: energy dissipation decreased from 133 to 75 kJ m^−3^ in 5 cycles at 300% strain	Sensors, human activity monitors, electronic skins, and soft electronic devices (Conductive)	[48]
		MPS-CSNPs	Agar/PHEAA	Tensile strength: 2.4 MPa; Tensile strain: 560%; Young’s modulus: 1.9 MPa; Tearing energy: 11.0 kJ m^−2^	Anti-counterfeiting, imaging, biosensing, and other optical applications (Fluorescence-responsive)	[49]
		dPEDOT NPs	CA/PDA /PAM	Tensile strain: 2500%; Young’s modulus: 1.9 MPa; Dynamic modulus: < 1.0 kPa, close to the modulus of soft brain tissue (0.2–3.1 kPa); Fracture energy: 1.35 ± 0.08 kJ m^−2^	Brain–machine interfaces (Conductive)	[50]
		GQDs	PVA/PNMA	Tensile strength: 303.06 kPa; Tensile strain: 198%; Compressive strength: 20.39 MPa (compressive strain 94.53%)	Deployable sensors for specific metal ions and information security and storage (Ionic-responsive)	[51]
		MOF (V-ZIF-8) NPs	Gelatin/PHEAA	Tensile strength: 2.4 MPa; Tensile strain: 1400%; Toughness: 14.10 MJ m^−3^	Imaging, sensing, and other optical applications (Fluorescence-responsive)	[52]
	1D nanomaterials	CNT-XLG	PAM/PAPBA	Tensile strength: 252–323 kPa; Tensile strain: 880–1200%; Compressive strength: 13.7 MPa; Young’s modulus: 48–50 kPa; Fracture energy of 911–1078 J m^−2^; Fatigue resistance: 100% tensile strain for 1000 cycles with residual strain < 9%	Personal healthcare, disease diagnosis, and implantable devices (Conductive)	[53]
		CNT	ACMO/ mPEG 480-acrylate	Tensile strength: 8.4 MPa; Tensile strain: 200%; Fatigue resistance: after 10 tensile cycles, the 200% strain-dried elastomer recovers to its original length within 1.5 h	Wearable sensors, flexible electronics, and health monitoring (Conductive)	[54]
		CNT	HAPAAm	Tensile strength: 0.267 MPa; Tensile strain: 3000%; Toughness: 3.42 MJ m^−3^; Fatigue resistance: following 1000 load-unload cycles at 100% strain, max stress, Young's modulus, and hysteresis ratio show negligible decay	Skin-like strain/pressure sensors (Conductive)	[55]
		TOCNF	PNIPAm/PVA/ PAM	Tensile strength: 1.97 MPa; Tensile strain: 344%; Work of fracture: 2907.5 kJ m^−3^	Invasive medical actuators, drug delivery systems, smart sensors and development of new—type functional materials. (Conductive)	[56]
		CNF	PAM	Tensile strength: 36 MPa; Young’s modulus: 310 MPa (along the growth direction); Tensile strength: 0.54 MPa; Young’s modulus: 0.135 MPa (perpendicular to the growth direction)	Soft tissues, intelligent sensor devices, and nanofluidic. (Conductive)	[57]
		ANF	PMAD/PVA	Tensile strength: 352.64 kPa; Tensile strain: 142.80%	Optical switch and sensor. (Thermal-responsive)	[58]
		TPU	PVA/ SA/ TA	Tensile strength: 1.05 MPa; Tensile strain:500%; Elastic modulus: 0.55 MPa; Fatigue resistance: under natural conditions, the material remains functional after 300 cycles at 30% strain; in wet conditions, after 800 cycles	Digital gesture recognition, wound dressings and patches, implantable medical devices, soft robotics, and environmental monitoring. (Conductive and humidity- responsive)	[59]
		FDA-SNF	PVA	Tensile strength: 7.84 ± 0.10 MPa; Toughness: 9.9 ± 0.4 MJ m^−3^; Elastic modulus: 13.71 ± 0.28 MPa; Fatigue resistance: no plastic deformation under 100 cycles of stretching	Artificial tendons and ligaments, joint connection parts of soft robots, external flexible grasping structures and energy absorption and buffering, wearable electronic devices. (Conductive)	[60]
	2D nanomaterials	MXene	Zn^2+^/SA/ PAM	Tensile strength: 2.19 MPa; Tensile strain: 305%; Tensile modulus: 769.10 kPa; Compressive strength: 0.35 MPa (compressive strain 80%). Energy dissipation: It undergoes 50 compression cycles at 80% strain with a 90.9% self-recovery rate	Human motion tracking, antibacterial wound healing and pressure sensing. (Conductive)	[61]
		MXene	Zr^4+^ /SA/PAM	Tensile strength: 2.68 MPa; Tensile strain:570%; Tensile modulus: 1.83 MPa; Toughness: 7.99 MJ m^−3^	Flexible wearable devices (such as sports monitoring and wireless transmission), tissue engineering, and energy storage. (Conductive)	[62]
		MXene-QACNF	PAM	Tensile strength: 449 kPa; Tensile strain: 1706%; Toughness: 5.46 MJ m^−3^	Flexible strain sensor. (Conductive)	[63]
		GO	PVA/PAM	Tensile strength: 300 kPa; Tensile strain: 2000%	Strain sensor, triboelectric nanogenerator. (Conductive)	[64]
		GO	CMCT/ PNIPAm	Tensile strength: 1.046 MPa; Tensile strain: 1286%; Compressive strength: 2.37 MPa (compressive strain 80%)	Biomedical sensor, remote actuator (Conductive, thermal-responsive, NIR-light-responsive and pH-responsive)	[65]
		GO	κ-Car/PAM	Compressive strength: 21.7 MPa; Tensile strength: 0.64 MPa; Tensile strain: 2398%; Fracture energy: 5.70 MJ m^−3^; Hysteresis: 0.98 MJ m^−3^ at λ = 10 Toughness recovery: ~ 97.23% at 90 °C and ~ 67.07% at room temperature	Stimulus-responsive load-bearing materials, drug delivery systems, wound dressings, flexible electronic devices, environmental protection, soft robotics, and aerospace. (Electrically responsive and thermally responsive)	[66]
		pBP nanosheets	PVA	Tensile strength: 2.71 MPa; Tensile strain: 400%; Compressive strength: meet the requirement of artificial articular cartilage (0.4–0.8 MPa)	Drug release, bone repair, wound healing, and biosensors (NIR-light-responsive, oxidation–reduction-responsive, and pH-responsive)	[67]
		f-BNNS- Clay	PNIPAm	Tensile strength: 40 kPa; Tensile strain: 980%	Strain sensor (Conductive)	[68]
Sacrificial bond theory	Double-network	PAAm/AcAG		Tensile strength: 8.0 MPa; Tensile strain: 1600%; Compressive strength: 50 MPa (compressive strain 90%); Toughness: 55 MJ m^−3^	Load-bearing damping material for a jointed robotic lander	[69]
		PMOx/PAA		Tensile fracture energy: 2.1 ± 0.50 kJ m^−3^; Compressive strength: 45 MPa (in PBS); Compressive strength: 60 MPa (in egg white)	Cartilage substitute material	[70]
		A11AUA/PAAm		Tensile strength: 0.75 MPa; Tensile strain: 3403%; Stiffness: 1016 kPa; Tearing energy: 7540 J m^−2^; Toughness: 17.53 MJ m^−2^	Tissue engineering, wound dressing, and soft robot	[71]
		PVA/SA-T@BT		Tensile strength: 52.3 kPa; Tensile strain: 1373.8%; Toughness: 587.10 kJ m^−3^; Adhesion strength to wood: 67.5 kPa	Epidermal sensor to detect signals of human movements like finger, writing as well as the bending of fingers, elbows, wrist, and knee	[72]
		PVA/PAA/GO		Tensile strength: 10.19 MPa; Tensile strain: 385%; Tensile modulus: 3.11 MPa; Compressive strength: 5.01 MPa (compressive strain 50%); Compressive modulus: 4.93 MPa. Friction coefficient: ~ 0.036 (after 500,000 cycles of friction)	Artificial joints	[73]
		PVA/PAA/Zr^4+^		Tensile strength: 11.6 MPa; Tensile strain: 219%; Young’s modulus: 428 MPa; Toughness: 19.8 MJ m^−3^	Miniature soft machines and robots	[74]
		PVA/PAA/Fe^3+^		Tensile strength: 53.0 ± 5.0 MPa; Tensile strain: 1374 ± 104%; Toughness: 315.7 ± 28.4 MJ m^−3^	Wearable soft strain sensors	[75]
	Dual-crosslinked	PAAM/PMAH/Zn^2+^		Tensile strength: 5.92 MPa; Tensile strain: 396%; Young’s modulus: 11.4 MPa;	Tissue engineering, drug mitigation, wearables, and soft robots	[76]
		P(NaSS-coDMAEAQ)		Tensile strength: 0.19–0.55 MPa; Young’s modulus: 0.08–0.4 MPa; Fracture strain: 500%-3300%; Fracture Energy: 1.5–4 kJ m^−2^	Fatigue-resistant soft materials	[77–79]
		Zr^4+^-P(AAm-coAMPS)		Tensile strength: 2.1–11.6 MPa; Young’s modulus: 0.45–28.5 MPa; Fracture strain:3320%-1350%; Fracture Energy: 1.5–4 kJ m^−2^	Biomedical and engineering fields	[17]
		PAAm–DVB		Tensile strength: 0.4–1.5 MPa; Young’s modulus: 0.027–0.9 MPa; Fracture strain: > 10,200%; Fracture Energy: 2.5–26 kJ m^−2^	Soft robots and intelligent devices	[80]
Topological network	High-entanglement	polyprotein (FL)_8_		Fracture stress: 390 kPa; Toughness: 250 + 68 k m^−3^; Fracture stress: 75 MPa; Fracture strain: ~ 107%	–	[81]
		AETC/4-BB@β-CD		Uniaxial Tensile Strain: 20,500%; Biaxial Tensile Strain: 10,000% in both directions; Tensile Toughness: 157 kJ m^−2^; Fracture energy: 157 kJ m^−2^	Luminescent materials, safety signs; wearable sensors; bioimaging, drug delivery, and tissue engineering; secure communication and data protection; smart window	[82]
		PAMPS/PAAm		Tensile strength: ∼3 MPa; Fracture energy: 8340 J m^−2^	Drug delivery	[83]
		PAAm/Laponite nanosheets		Tensile strength: 4.2 MPa; Modulus: 50 MPa	Soft robotics, additive manufacturing and biomedical applications	[84]
		PLA/PEG		Toughness: 1.2–3.9 MJ m^−3^; Elastic modulus: 0.15–4 MPa	Osteochondral repair	[85]
		PU/Copper(I) ion-coordinated 2,9-bis(hydroxymethyl)phenyl-1,10-phenanthroline		Tensile strength: 33.5 ± 0.5 MPa; Strain capacity: 4000 ± 280%; Cyclic stability: stable performance over 7000 cycles	–	[86]
		PAAm/TOCNFs/ [BMIm]Zn_x_Cl_y_		Tensile Strength: 5.9 MPa; Elastic Modulus: 30.4 MPa; Toughness: 22 MJ m^−3^	Wearable self-powered sensors, biomechanical monitoring, smart writing sensors, and smart healthcare	[87]
		Polyimide/poly-(vinyl pyrrolidone)		Tensile modulus: 22.57 MPa; Tensile strength: 2.14 MPa	–	[88]
	Slide-ring	PEG_4k_ -(azo)_2_/γ-CD/ HEMA		Tensile strength: 200 kPa; Tensile strain: 1140%; Elastic modulus: 520 kPa; Toughness: 2.1 MJ m^−3^	Sensing devices by 3D printing	[89]
		PEG/hydroxypropyl-α-CD rings/divinyl sulfone		Toughness: 6.6–22 MJ m^−3^; Almost 100% rapid recovery of extension energy	–	[90]
		HAMA/ NIPAAm		Tensile strain: 4100%; Fracture energy: 78,500 J m^−2^; Elastic modulus: 24–41 kPa	Wound healing	[91]
		Alg/PEG/hydroxypropyl-α-CD rings		Tensile strength: 199 ± 20 kPa; Tensile strain: 1239% ± 58%; Compressive stress: ∼200 kPa at 70% strain; Fracture energy: 668 ± 80 J m^−2^	Biomedicine	[92]
		PAAm/ Alg/ Ca^2+^/LCA-PEG_2K_-AC/β-CD-AC		Tensile strength: 449.3 kPa; Tensile strain: 2572%; Toughness: 8803.2 kJ m^−3^	Textile-based stretchable electronic devices	[93]
		PAAm/PR-PEGMA/ PEDOT: PSS		Tensile strength: 119 kPa; Tensile strain: 4363%; Elastic modulus: 45 kPa	Implantable neural electrodes	[94]
		Polypseudorotaxane/AAm		Fracture energy: 35.84 MJ m^−3^	Stretchable supercapacitor	[95]
Hard–soft phase structure	PA hydrogel	Zr^4+^-P(NaSS-co-DMAEA-Q)		Tensile strength: 3.7 MPa; Toughness:3.5 MJ m^−3^; Young’s modulus: 39.2 MPa	–	[96]
		pVBIPS/Fe (ClO_4_)^3^		Tensile strength: 1.34 MPa; Toughness: 2.29 MJ m^−3^; Young’s modulus: 1.2 MPa. Fracture energy: 19.55 kJ m^−2^	Soft strain sensor	[97]
		PVA/PSBMA-H_2_SO_4_		Tensile strength: 2.01 MPa; Tensile strain: 520%; Toughness: 4.61 MJ m^−3^; Young’s modulus: 127.3 kPa; Compressive strength: 12.71 MPa; Energy dissipation: 50 cycles, stress drops from 115 to 100 kPa, dissipated energy from 0.298 to 0.227 kJ m^−3^	Underwater hydrogel-based electronic equipment	[98]
		SBMA/MBAA/TA@BCNF/TA@XLG Nanoflowers		Tensile strain: > 2900%; Toughness 1.16 MJ m^−3^; Young’s modulus: < 10 kPa	Implantable electrodes and sensing devices	[99]
Self-growth theory	Self-growth hydrogel	pVB@xSn		Fracture energy: 1334.0 J m^−2^; Fatigue threshold: 720 J m^−2^	Wearable strain sensor and bioelectrode	[100]
		PBA/PAAm/Ca_3_(PO_4_)_2_		Tensile strength: 3.3 MPa; Young’s modulus: 120 MPa; Fracture energy: 1500 J m^−2^; Ballistic energy absorption: 1.5 kJ m^−2^; Puncture strength: 12 N; Bending strength: > 13.5 MPa	Flexible bulletproof vests, impact-resistant coatings for implantable devices and intelligent protection systems	[101]
		PAAm/PEGDA/NaAc		Young’s modulus:1.24 MPa (increased by 51.5 times)	/	[102]
		PVA/TA/PEGDA		Tensile strength: 14.1 MPa; Young’s modulus: 2.85 MPa; Toughness: 465 MJ m^−3^; Energy dissipation: energy dissipation efficiency ~ 90%	Impact protection material, surgical suture	[103]

### Concentrated Crosslinking Point Theory

The concentrated crosslinking point theory describes a toughening mechanism wherein polymer chains are physically or chemically tethered to nanomaterials that act as multifunctional crosslinking nodes. These nanomaterials, often modified at the surface, form covalent bonds or strong physical interactions with surrounding polymer chains, creating a dense and robust crosslinked network. The resulting architecture enhances mechanical performance by promoting load transfer, reducing stress concentration, and improving network uniformity. This concept underpins the design of NC hydrogels, which are among the earliest and most prominent hydrogel systems engineered for mechanical reinforcement. Haraguchi et al. [25] first reported an NC hydrogel composed of poly(N-isopropylacrylamide) (PNIPAAm) and clay nanosheets (CNS), synthesized without any traditional chemical crosslinker. In this system, clay platelets serve dual functions: They physically adsorb numerous polymer chains and act as inorganic crosslinkers bridging distinct polymer segments. The resulting hydrogel exhibited exceptional mechanical properties, including an elongation at break exceeding 1000% and a tensile strength reaching 100 kPa, while maintaining excellent flexibility and bendability.

#### 0D Nanocomposite Hydrogels

**Fig. 3:**
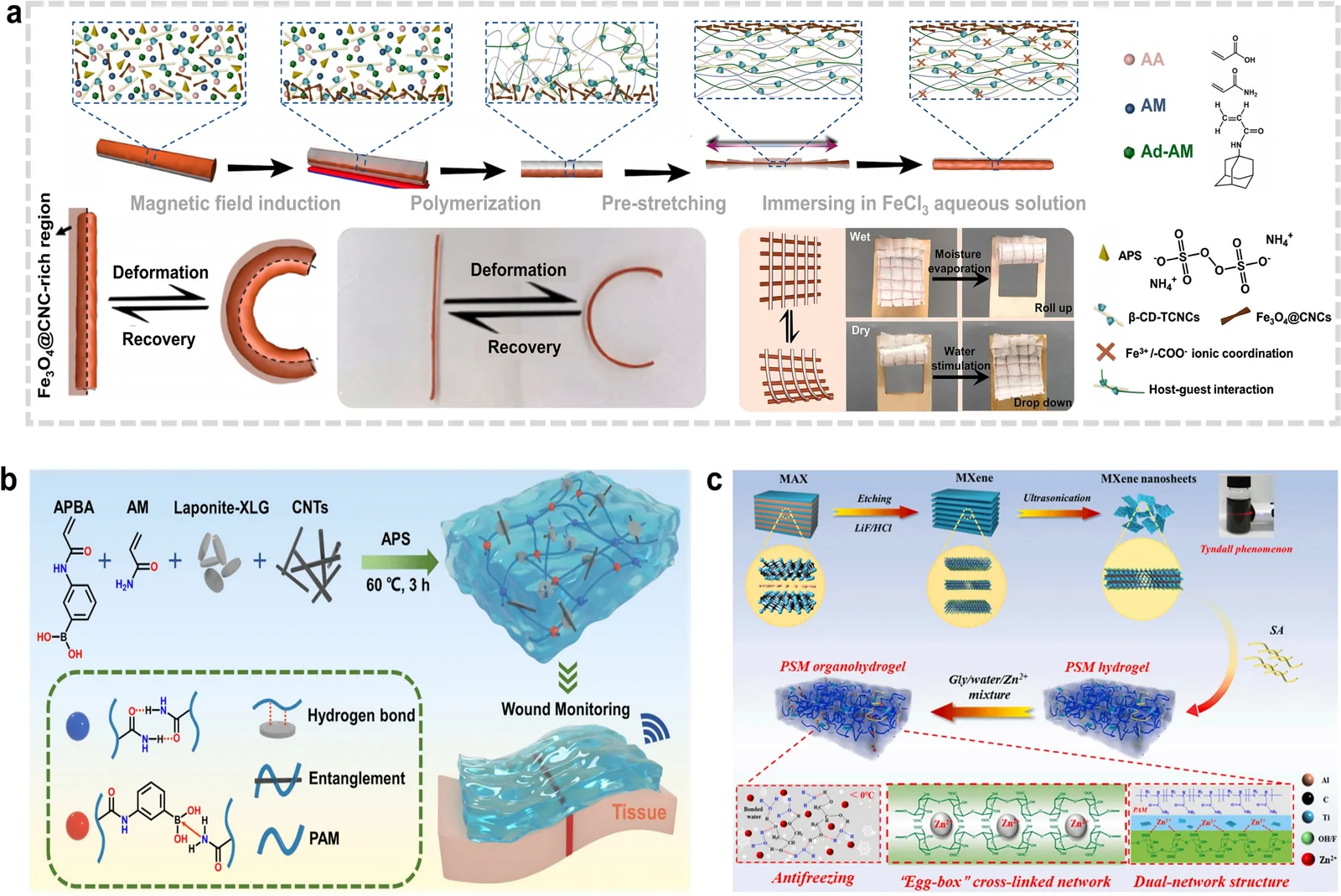
Mechanisms of mechanical enhancement in nanocomposite hydrogels reinforced with nanofillers of different dimensionalities. **a** 0D fillers: Fe_3_O_4_ NPs enabling magnetic responsiveness and network reinforcement. **b** 1D fillers: CNTs, and ANFs enhancing tensile strength, fatigue resistance, and structural entanglement. **c** 2D fillers: MXene nanosheets forming layered “egg-carton” structures for compressive strengthening and interfacial adhesion. Reproduced with permission from [45, 53, 61, 117]

Zero-dimensional (0D) nanomaterials are characterized by nanoscale dimensions in all directions, typically existing as spherical nanoparticles or nanoclusters. These materials, encompassing polymers, metals, metal oxides, and semiconductors, exhibit distinctive physicochemical properties, including size-dependent magnetic, optical, and electronic behavior [116]. Owing to their tunable surface chemistry and large surface-area-to-volume ratio, 0D nanomaterials have been widely employed as reinforcing agents in the fabrication of mechanically extreme hydrogels. Through chemical modification or surface functionalization, these nanoparticles can form robust interfacial interactions with polymer networks such as covalent bonding, hydrogen bonding, or coordination interactions, which significantly improve stress transfer efficiency and network integrity. By physically embedding 0D nanoparticles as dissipation sites, hydrogels gain enhanced fracture toughness and fatigue resistance, while acquiring multifunctional responsiveness like thermosensitivity or conductivity, paving the way for applications in smart actuators and wearable sensors. Zhai et al. [62] engineered magnetically responsive hydrogel fibers using Fe_3_O_4_-modified cellulose nanocrystals (Fe_3_O_4_@CNCs). Under an applied magnetic field, the Fe_3_O_4_@CNCs aligned within the hydrogel matrix, generating an anisotropic internal structure that endowed the fibers with excellent tensile properties (tensile strength and Young’s modulus both reaching 28.9 MPa, and toughness of 18.2 MJ m^−3^) and fast deformation under magnetic stimuli. Unlike SiNPs, which primarily enhance stiffness and water transport, Fe_3_O_4_@CNCs serve as magneto-responsive agents, enabling structural orientation and remote actuation through magnetic field control and ionic coordination interactions (Fig. 3a).

#### 1D Nanocomposite Hydrogels

One-dimensional (1D) nanomaterials are characterized by nanoscale dimensions in one direction and extended lengths in the other two, typically forming fibrous, rod-like, or tubular structures. Their high aspect ratios and anisotropic geometries make them particularly effective in enhancing the mechanical strength and toughness of hydrogels by promoting efficient stress transfer and crack deflection under tension or compression. These materials exhibit excellent intrinsic mechanical properties such as high tensile strength, stiffness, and energy dissipation capacity, which are imparted to the hydrogel matrix through strong interfacial interactions, including hydrogen bonding, electrostatic interactions, or covalent linkages. Following this, Shen et al. [53] successfully achieved the uniform dispersion of CNTs in aqueous solution by utilizing a synthetic layered silicate of LAPONITE@ XLG nanosheets as surfactants. They subsequently synthesized P(AAM-APBA)-XLG/CNT hydrogels via a one-step free-radical copolymerization process. The resulting hydrogel exhibited excellent elasticity and fatigue resistance, enabled by B-N coordination, hydrogen bonding, and polymer chain entanglement. Remarkably, the material maintained mechanical integrity over 1,000 consecutive tensile and compressive cycles, underscoring its potential for durable applications in high-strain environments (Fig. 3b). Additionally, the hydrogel demonstrated outstanding sensing capabilities, accurately detecting strain levels from 1 to 300% and pressure changes in the range of 1–80 kPa, making it highly suitable for wearable sensing and soft electronic interfaces. In a parallel study, Xing et al. [117] incorporated ANFs into a UV-curable hydrogel precursor, enabling the fabrication of ANF-reinforced 3D-printable hydrogel composites. With only 0.3 wt% ANFs, the composite exhibited a 30-fold increase in elastic modulus, a tenfold enhancement in tensile strength, nearly an order-of-magnitude improvement in fracture energy, and a 5.3–12.6 times higher fatigue threshold compared to the pure hydrogel.

#### 2D Nanocomposite Hydrogels

Two-dimensional (2D) nanomaterials are defined by their nanoscale thickness (typically 1–100 nm) and extended lateral dimensions in the other two directions, forming sheet-like structures with ultrahigh aspect ratios. First, their large surface area and high surface energy promote strong interfacial interactions such as hydrogen bonding, electrostatic attraction, or π-π stacking with polymer chains [118]. This improves load transfer efficiency, reduces interfacial slippage, and reinforces the gel network at both nano- and microscales. Second, the layered architecture formed by aligned or randomly dispersed nanosheets creates tortuous paths for crack propagation, which effectively hinders microcrack extension and enhances toughness. In some systems, 2D fillers can also promote network rearrangement or induce physical crosslinking, further contributing to energy dissipation and enhanced structural integrity. Additionally, the strong attraction between nanosheets and surrounding hydrogel components can lead to localized densification and the formation of interconnected gel domains, which restrict molecular mobility, increase cohesive strength, and allow stress coupling across the matrix [119]. This produces a comprehensive hardening effect that enhances tensile strength, compressive modulus, and fatigue resistance, thereby providing key characteristics of hydrogels specifically engineered for extreme mechanical environments.

Following these strategies, Wang et al. [61] developed a MXene-reinforced DN hydrogel by incorporating MXene nanosheets into a polyacrylamide (PAM) and sodium alginate (SA) matrix. The MXene nanosheets not only provided a large specific surface area, promoting strong interfacial interactions with the polymer network, but also facilitated the formation of an “egg-carton” crosslinked architecture, which significantly enhanced mechanical resilience. As a result, the hydrogel’s compressive strength increased from 0.17 to 0.35 MPa, and it retained 90.9% recovery after 50 compression cycles at 80% strain, demonstrating outstanding elastic recovery and fatigue resistance. Additionally, the tensile strength was remarkably improved from 28.1 kPa to 2.19 MPa, highlighting the robust reinforcement capability of 2D MXene fillers (Fig. 3c). In a complementary study, Chen et al. [120] engineered a high-stiffness, fast-response hydrogel actuator using GO and deoxyribonucleic acid (DNA). In this system, GO sheets induced a layered heterogeneous structure, comprising two GO outer layers and a central layer of double-stranded DNA (dsDNA). Upon stimulus, dsDNA was reversibly converted into single-stranded DNA (ssDNA), and the resulting structure, stabilized by extensive hydrogen bonding interactions, conferred an ultrahigh Young’s modulus of up to 10 GPa alongside rapid volumetric swelling. This design exemplifies how 2D materials can be leveraged not only for mechanical strengthening but also for stimulus-induced structural transformation, achieving multi-responsive performance beyond conventional hydrogels.

While 0D, 1D, and 2D nanofillers all operate through the concentrated crosslinking point mechanism, their geometric dimensionality fundamentally dictates distinct toughening mechanisms and performance profiles. This “dimensionality effect” means that nanomaterials of different shapes enhance hydrogels in unique ways, as systematically compared in Table 1. Leveraging this effect, the combined use of multi-dimensional nanofillers presents a powerful strategy for creating smart hydrogels with simultaneous reinforcement and advanced functionality (Table 3). Rooted in the concentrated crosslinking point theory, nanofillers enhance the mechanical performance of hydrogels by increasing crosslinking density and introducing synergistic mechanisms such as traction strengthening [121] and void-bridging toughening [122]. This multiscale approach significantly improves tensile strength, crack resistance, and overall structural integrity, which are properties essential for hydrogels in extreme mechanical environments. Furthermore, by combining dimensions, materials gain integrated functional properties such as electrical conductivity, optical responsiveness, and sensitivity to smart responses [123].

In the toughening strategies of hydrogels, 0D, 1D, and 2D nanomaterials have been widely studied due to their unique structures and properties. However, these nanomaterials still face numerous challenges and limitations in practical applications. For instance, 0D nanomaterials tend to agglomerate, which affects their uniform dispersion in the hydrogel network and thus reduces the toughening effect. Additionally, insufficient interfacial interactions between nanoparticles and the polymer matrix may lead to low stress transfer efficiency, thereby impacting the overall performance of the material. 1D nanomaterials can effectively disperse stress and limit crack propagation, but they may fail under high-strain conditions due to structural damage. 2D nanomaterials, although possessing excellent mechanical properties, may reduce the transparency and biocompatibility of hydrogels, limiting their application in the biomedical field. Therefore, when designing mechanically extreme hydrogels, it is crucial to balance reinforcement efficiency, functional integration, and safety considerations. A comprehensive understanding of nanomaterial interactions at molecular and network scales will be essential to optimize both mechanical toughness and application-specific viability.

**Table 3:** Summary of six critical target properties (fracture energy/toughness, fatigue resistance, extensibility, strength, modulus control) with biological/synthetic reference ranges, matched hydrogel systems, and corresponding toughening mechanisms, providing a theoretical framework for multiscale toughening design

Target property	Reference data range	Matching hydrogel type	Mechanism mapping	References
Fracture Energy and Toughness	*Fracture Energy:* Articular Cartilage: ~ 10^3^ J m^−2^; Tendon: ~ 10^3^ J m^−2^; Rubber: ~ 10^4^ J m^−2^. *Toughness:* Spider Silk: ~ 150 MJ m^−3^;	Double-network Hydrogels; Nanocomposite Hydrogels	*Bio-inspired Energy Dissipation:*1. Rigid network fracture in DN gels (mimics tendon collagen).2. Nanofiller bridging cracks (mimics rubber carbon black)	[104–107]
Fatigue Resistance	Tendon: 10^4^-10^6^cycles; Cartilage 10^6^–10^8^ cycles; Rubber: 10^6^–10^9^ cycles	Slide-ring Hydrogels; Dual-crosslinked Hydrogels	*Dynamic Stress Buffering:* 1.Topological disentanglement in slide-ring gels (mimics rubber entropy elasticity).2. Self-healing dynamic bonds (mimics tendon protein remodeling)	[105, 108, 109]
Extensibility	Rubber: 500%-800%	Highly-entangled Hydrogels; Dual-crosslinked Hydrogels	*Chain-slip Dominated Deformation:*1. Physical entanglement enables chain slippage (mimics rubber supercoiled chains).2. Dynamic bond reorganization (mimics skin elastin)	[110]
Strength	Tendon: 50–150 MPa (Tensile Strength); Knee Cartilage: 4–9 MPa (Compressive Strength); Spider Silk: 0.8–1.4 GPa (Tensile Strength)	Double-network Hydrogels; Nanocomposite Hydrogels	*Inter-scale Energy Transfer and Dissipation:* 1. Sacrificial Network Fracture. 2. Nanofiller-induced Crack Pinning	[111–113]
Modulus Control	*Biological Tissues:* Softest: Brain tissue (kPa); Stiffest: Bone interface (GPa); Intermediate: Tendon (MPa); *Synthetic Materials:* Soft: Rubber (MPa); Stiff: Plastics (GPa)	Nanocomposite Hydrogels; Electrolyte Hydrogels	*Multiscale Stiffness Design:* 1. Nanofillers directly elevate modulus (mimics plastic rigid skeleton). 2. Ion concentration tunes crosslink density (mimics skin gradient structure)	[114, 115]

### Sacrificial Bond Theory

The sacrificial bond theory, first conceptualized in hydrogels by Gong et al. in 2003 [26], has since become a foundational framework for designing mechanically extreme hydrogels with high toughness and energy dissipation capabilities. In their pioneering study, bilayered poly(2-acrylamido-2-methyl-1-propanesulfonic acid) (PAMPS)/PAM double-network hydrogels were synthesized, demonstrating remarkable mechanical enhancement through the introduction of hierarchical bonding interactions. The principle of sacrificial bonding is based on the strategic incorporation of “weak” and “strong” bonds within the hydrogel network. Upon mechanical loading, weak, reversible bonds such as ionic interactions or hydrogen bonds act as sacrificial units, breaking first and dissipating energy to delay catastrophic failure. These bonds absorb deformation energy and prevent immediate structural collapse. Meanwhile, strong covalent bonds serve to maintain network integrity, supporting the load after the sacrificial bonds are ruptured. In the case of PAMPS/PAAm DN hydrogels, the first network comprised of densely crosslinked PAMPS is highly brittle and composed of pre-stretched polymer chains that cannot accommodate further deformation. When stress is applied, this brittle network fractures early, functioning as the sacrificial phase. In contrast, the second network, based on loosely entangled PAAm chains, remains intact under deformation. These long, flexible chains gradually straighten under load and eventually sustain stress via covalent bond rupture, enabling the hydrogel to maintain structural continuity and stretchability. This double-network architecture thus couples brittle energy dissipation with elastic resilience, producing a hydrogel with high fracture energy and extreme deformation tolerance.

#### Double-Network Hydrogel

Double-network (DN) hydrogels are a class of polymeric materials consisting of two interpenetrating or semi-interpenetrating three-dimensional networks, each with distinct mechanical roles and bonding characteristics. This unique architecture effectively addresses the intrinsic mechanical limitations of single-network hydrogels, offering a robust balance between strength, toughness, and flexibility. DN hydrogels are typically categorized based on their crosslinking mechanisms into three types: (1) fully chemically crosslinked, (2) fully physically crosslinked, and (3) physically–chemically hybrid crosslinked systems. While chemically crosslinked DN hydrogels exhibit high mechanical strength, they are often prone to irreversible damage due to the permanent nature of covalent bonds. In contrast, the latter two types incorporate dynamic non-covalent interactions, such as hydrogen bonding, ionic coordination, or hydrophobic associations, which function as sacrificial bonds capable of reversible breakage and reformation. In hybrid double-network systems, the primary network is typically covalently crosslinked and acts as a structural backbone, while the secondary network, composed of reversible physical bonds, provides a dissipative phase that absorbs deformation energy under stress. This hierarchical bonding strategy prevents stress overshoot and crack propagation, significantly enhancing both fracture energy and fatigue resistance [111, 112]. The reversible nature of the physical bonds also contributes to self-recovery and damage tolerance, which are essential for applications involving cyclic or high-impact mechanical loading.

**Fig. 4:**
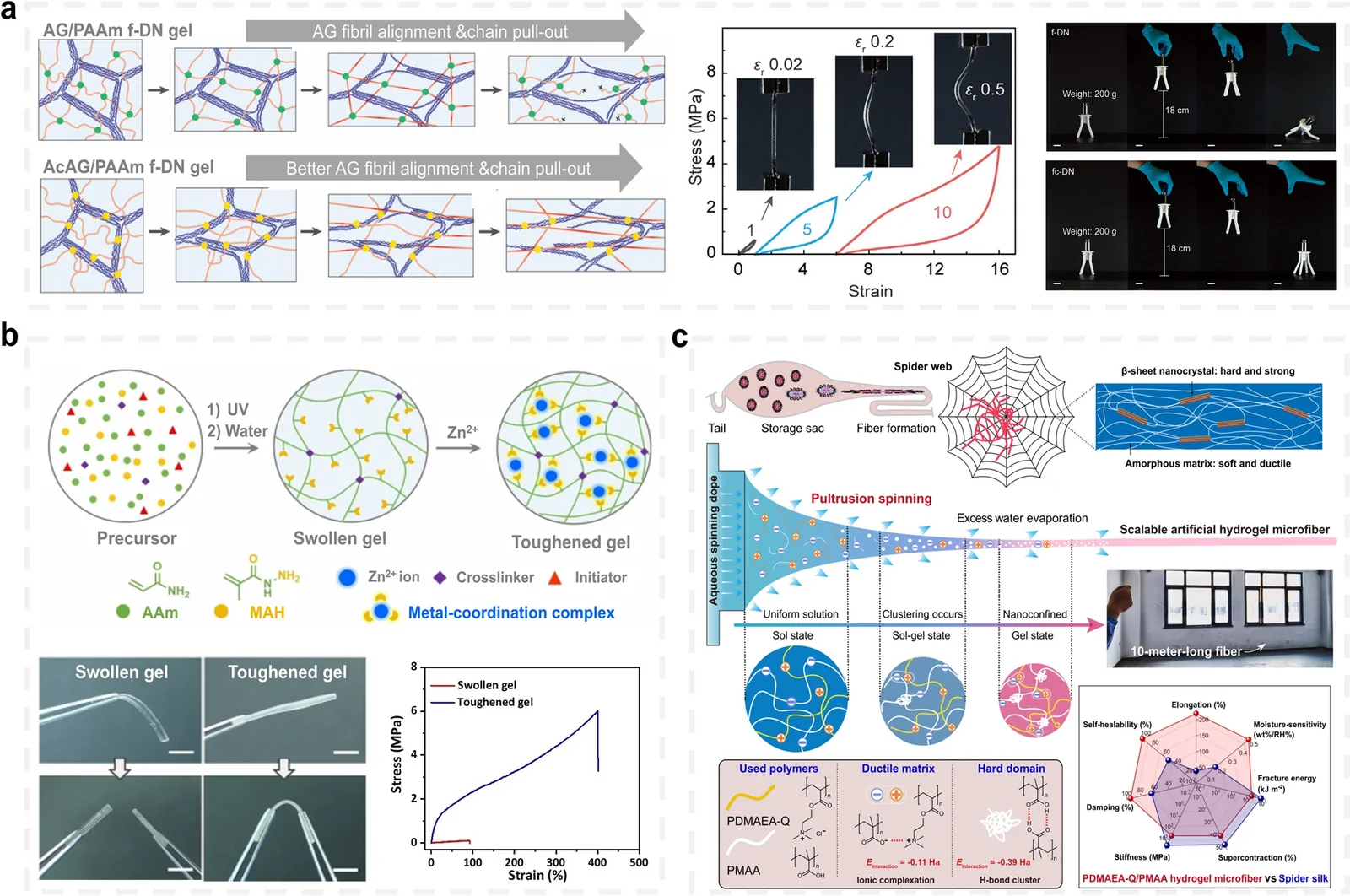
Representative hydrogel systems based on the sacrificial bond theory. **a** Fiber-connected double-network (fc-DN) hydrogel with enhanced stress transfer and fiber alignment for improved toughness and tensile strength. **b** Dual-crosslinked hydrogel incorporating hydrazone–Zn^2+^ coordination complexes as dynamic crosslinkers, enabling both mechanical reinforcement and autonomous repair. **c** Spider silk-inspired hydrogel microfibers, fabricated via a drawing-spinning technique, featuring hydrogen-bonded networks for high fracture energy and rapid self-healing. Reproduced with permission from [69, 74, 76]

Specifically, Fang et al. [69] developed a fiber-connected double-network (fc-DN) hydrogel by integrating PAM with an acrylic acid-modified agarose (PA) fiber network. The introduction of interconnected fiber structures facilitated improved stress transfer, enhanced network alignment, and load-bearing capacity, resulting in a hydrogel with a tensile strength of 8 MPa and an exceptional toughness exceeding 55 MJ m^−3^ (Fig. 4a). This design strategy demonstrates remarkable potential for mechanically extreme applications, such as damping materials in robotic landing gear, where impact absorption and energy dissipation are critical. In a parallel effort, Paola et al. [70] engineered a double-network hydrogel composed of crosslinked poly(2-oxazoline) (POx) and PAAm. In this system, reversible hydrogen bonding functions as a sacrificial network, dissipating stress during deformation and preserving the structural integrity of the chemically crosslinked backbone. The resulting hydrogel exhibited a compressive strength of 60 MPa, reflecting its ability to withstand extreme compressive loading.

Gong et al. [26] were the first to construct a typical DN hydrogel system through a two-step free-radical polymerization strategy: the highly crosslinked, rigid, and brittle poly-2-acrylamide-2-methylpropanesulfonic acid (PAMPS) as the first network, which serves as a sacrificial network to dissipate the energy; and the low-crosslinked, flexible, and ductile PAM as the second network to maintain the overall stability of the structure. The system exhibits excellent mechanical properties: toughness up to 10^2^–10^3^ J m^−2^, fracture stress up to 1–10 MPa, and tensile strain up to 1000%-2000%. On this basis, Gong’s team [22] further proposed key design principles for DN hydrogels: The first network should be a rigid, highly crosslinked brittle polymer to assume the sacrificial role; the second network should be a soft, highly ductile neutral polymer with a molar concentration 20–30 times that of the first network; the crosslinking densities of the two networks should be significantly. The crosslinking density of the two networks should be significantly differentiated, with the first network being used for energy dissipation and the second network ensuring elasticity and integrity. Subsequently, Suo et al. [124] systematically investigated the fatigue fracture behavior of PAMPS/PAAm double-network hydrogels proposed by Gong’s group. The results show that the fatigue fracture threshold of the material decreases significantly when the chain length of the second network (PAAm) is shortened, indicating that the flexible chain segments play a key role in crack extension inhibition.

Although double-network and multilayer network hydrogels exhibit excellent toughness, they have significant limitations: First, pronounced hysteresis effects hinder full recovery during stretch-relax cycles, limiting their use in dynamic load environments like soft robotics. Second, they often undergo permanent softening after large deformations due to irreversible bond fracture in the first network, leading to performance decline. Third, while sacrificial bond fracture dissipates energy, it can also trigger macroscopic irreversible damage during crack propagation. Fourth, increasing network layers enhances toughness but introduces structural complexity, manufacturing challenges, and new failure modes like interfacial debonding. Thus, these designs still face practical challenges, necessitating new toughening mechanisms for more efficient and stable hydrogel materials.

#### Dual-Crosslinked Hydrogel

Dual-crosslinked hydrogels are engineered by synergistically integrating covalent crosslinks and dynamic non-covalent crosslinks. Defined by a stable covalent network framework coupled with reversible non-covalent bonds acting as sacrificial motifs, their toughening mechanism relies on efficient hierarchical energy dissipation. Under mechanical load, dynamic bonds reversibly rupture to dissipate energy, redistributing stress concentrations and shielding the covalent network from catastrophic failure. This process effectively suppresses crack propagation and enhances fracture energy.

Specifically, Ju et al. [76] developed a poly(acrylamide-co-methylacrylamidrazone) [P(AAm-co-MAH)] hydrogel reinforced with hydrazone–Zn^2+^ coordination complexes (Fig. 4b). These metal–ligand complexes serve as dynamic and reversible physical crosslinks, enabling efficient energy dissipation during mechanical deformation while preserving the network integrity. By tuning the copolymer composition and Zn^2+^ ion concentration, the mechanical and self-healing properties of the hydrogel can be systematically optimized. The hydrogel also exhibited shape memory behavior, expanding its potential for programmable and multifunctional soft devices. In a parallel approach inspired by the mechanics of spider silk, Shi et al. [74] fabricated self-healing hydrogel microfibers via an energy-efficient drawing–spinning method using poly(methacrylic acid) (PMAA) and poly(2-(dimethylamino)ethyl methacrylate) methyl chloride salt (PDMAEA-Q). These fibers form hydrogen-bonded supramolecular networks, which contribute to high fracture energy, rapid self-repair, and damping behavior under cyclic loading (Fig. 4c). The resulting microfibers demonstrated superior toughness and durability, making them promising for applications involving repetitive motion, shock absorption, or structural deformation.

Double-crosslinked hydrogels have emerged as a promising strategy to enhance the mechanical properties of hydrogels, exhibiting significant potential in biomedical fields such as tissue engineering and drug delivery. However, their practical application is still hindered by several challenges, including the irreversible nature of covalent crosslinking networks, the insufficient stability of physical crosslinking networks, complex fabrication processes, and issues related to biocompatibility. In particular, when applied under physiological conditions, double-crosslinked hydrogels often suffer from problems such as fracture, limited self-healing ability, unstable swelling behavior, and inadequate energy dissipation mechanisms. Future research should focus on optimizing the structural design of double-crosslinked hydrogels to enhance their stability and functionality in complex physiological environments, thereby promoting their widespread use in biomedical applications. In physiological conditions, the performance of hydrogels can be influenced by various factors, including temperature, pH value, and ionic concentration. The stimulus-responsive behavior of double-crosslinked hydrogels can be achieved by introducing dynamic crosslinking networks, such as borate ester bonds and disulfide bonds. However, further research is needed to improve the response speed and controllability of these systems.

### Homogeneous Network Theory

As research on DN hydrogels has advanced, attention has turned to a critical limitation of conventional systems, that is, network inhomogeneity at the nanoscale. During random polymerization processes, DN hydrogels often exhibit structural defects such as polymer chain looping [125], entanglement [126], and chain isolation [127]. These imperfections result in uneven polymer chain lengths and irregular crosslinking densities, which compromise the gel’s structural uniformity. Under mechanical loading, stress tends to concentrate in these defect regions, accelerating crack initiation and propagation, ultimately leading to catastrophic failure. To address this, the homogeneous network theory was proposed, serving as a guiding principle for the design of topological hydrogels, i.e., a new class of materials characterized by well-defined, defect-minimized network architectures. While the sacrificial bond theory achieves energy dissipation through the introduction of breakable “weak bonds,” the homogeneous network theory enhances mechanical robustness by focusing on the topological structure of the network itself. Its core concept does not rely on localized, predetermined sacrificial sites, but aims to construct uniform, reversible networks with distinctive topological features (e.g., high entanglement or slide-ring structures). This enables homogeneous stress distribution (stress homogenization) and highly efficient, reversible energy dissipation. As a result, it significantly reduces hysteresis and improves fatigue recovery, functioning in a complementary manner to sacrificial bonding.

#### High-Entanglement Hydrogel

High-entanglement hydrogels are characterized by polymer networks in which chain entanglements far outnumber chemical crosslinking points. The dense entanglement network enables stress to be effectively transferred along the contour length of polymer chains and shared among neighboring chains. In contrast, sparsely distributed chemical crosslinks serve mainly to maintain overall network cohesion without restricting chain mobility. This architecture yields hydrogels with enhanced toughness, greater extensibility, and superior fatigue resistance under repeated deformation. When subjected to external loading, the deformation energy in high-entanglement hydrogels is partitioned: A portion is stored elastically within the entangled network due to the inherent elasticity of the polymer chains, while the remainder is dissipated through viscous flow and internal friction, providing effective energy dissipation and damage delay mechanisms. This design strategy rooted in the homogeneous network theory provides a robust framework for engineering hydrogels with mechanical resilience.

**Fig. 5:**
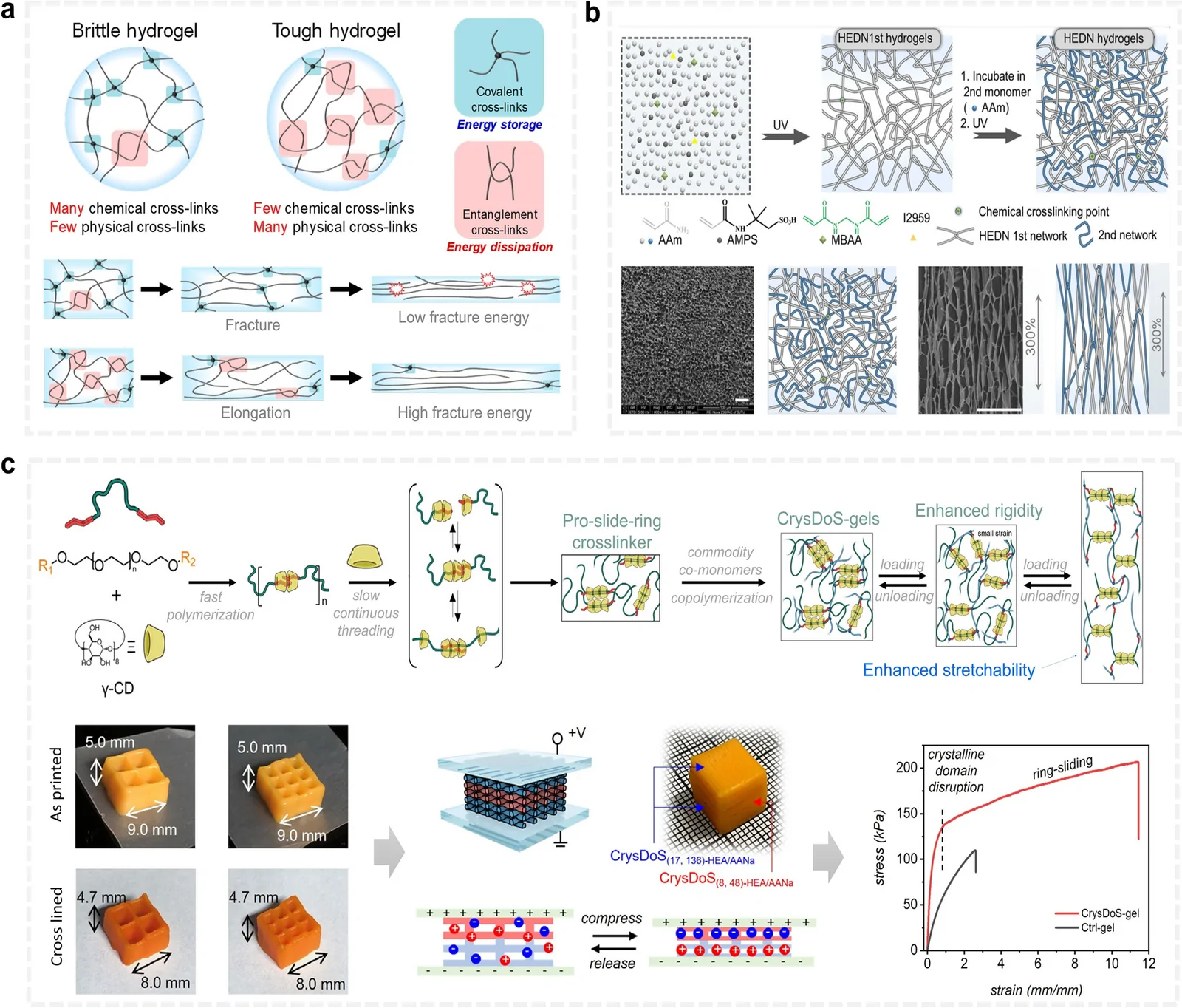
Representative hydrogel systems based on the homogeneous network theory. **a** High-entanglement hydrogel formed through physical crosslinking, enabling efficient energy dissipation and enhanced stress distribution under deformation. **b** Double-network high-entanglement hydrogel, in which swelling of the second network is controlled via electrolyte modulation in the first network, allowing tunable mechanical properties. **c** Slide-ring hydrogel formed by PEG with methacrylate–azobenzene end groups and *γ*-cyclodextrin, introducing double-penetration architecture and crystallization to improve elongation and toughness. Reproduced with permission from [83, 89, 128]

Specifically, Norioka et al. [128] enhanced hydrogel toughness by reducing the use of chemical crosslinking agents, thereby promoting the formation of a physically crosslinked, entanglement-rich network (Fig. 5a). This architecture increased the hydrogel’s viscous response, allowing part of the mechanical energy to be dissipated through chain mobility and internal friction, which mitigated stress concentration and delayed crack propagation. During stretching, the entangled network efficiently distributed stress and facilitated energy dissipation, resulting in excellent mechanical recovery and improved toughness. Building upon this principle, Zhu et al. [83] developed a double-network hydrogel in which the first network was created using a high monomer concentration with minimal crosslinking, followed by immersion in a second monomer solution to form the secondary network (Fig. 5b). By tuning the electrolyte content in the primary network, they precisely controlled the swelling behavior of the second network, thereby regulating the hydrogel’s mechanical properties. The physical entanglements in the first network played a crucial role during deformation, promoting uniform alignment and significantly enhancing tensile strength and fracture energy. In a complementary approach, Kim et al. [38] focused on the role of polymer chain entanglement in single-network hydrogels, showing that when chains are sufficiently long and the number of crosslinking points is kept low, the material achieves a favorable balance of rigidity and ductility. In such systems, entangled polymer chains act as sliding filaments, enabling effective stress redistribution without sacrificing deformability.

Despite the excellent toughening performance of entanglement strategies in hydrogels, they also exhibit significant limitations and failure modes. First, entanglement strategies may lead to performance degradation under extreme environmental conditions. For example, in low-temperature conditions, the elastic modulus of hydrogels may significantly increase, making them too rigid at low temperatures and thus affecting their application in cold environments. Second, entanglement strategies may result in structural instability during long-term use. For example, under cyclic loading or extreme environmental conditions, the destruction of entangled structures may lead to irreversible degradation of material performance.

#### Slide-Ring Hydrogel

Slide-ring (SR) hydrogels are a class of supramolecular materials characterized by topologically interlocked polymer networks in which cyclic molecules (rings), typically cyclodextrins, thread through linear polymer chains and form movable crosslinking points. These unique “figure of eight” or sliding crosslinks can reposition themselves along the polymer backbone when subjected to stress, enabling a pulley-like effect that dynamically redistributes mechanical forces across the networks [129]. This property imparts SR hydrogels with exceptional extensibility, toughness, and energy dissipation, making them ideal candidates for mechanically extreme and multifunctional applications. A pioneering example was reported by Okumura et al. [20] in 2001, who constructed a topologically connected hydrogel by threading poly(ethylene glycol) (PEG) chains through *α*-cyclodextrin rings, which were then capped and chemically crosslinked to form sliding crosslinking nodes. Under external load, these nodes could slide freely, reducing localized stress concentrations and preventing single-chain rupture. As a result, the hydrogel exhibited extraordinary mechanical properties, including elongation up to 2,000% and a swelling capacity exceeding 400 times its dry weight, a testament to its flexible yet structurally stable design. Further advancing this concept, Tang et al. [89] developed SR hydrogels using PEG chains with methacrylate–azobenzene termini that formed dual-penetration complexes with *γ*-cyclodextrins (Fig. 5c). This design enabled photo-responsive crystallization and chemically tunable mechanical properties, enhancing both the Young’s modulus and elongation capacity. These hydrogels also showed excellent compatibility with multi-material 3D printing and demonstrated potential for use in flexible stress sensors and adaptive structural materials. In summary, the slide-ring topology offers a versatile platform for designing homogeneous, stretchable, and fatigue-resistant hydrogels. By integrating sliding crosslinks, dynamic crystallization, and stimuli-responsive motifs, SR hydrogels bridge the gap between mechanical performance and smart material functionality, expanding their utility in soft robotics, wearable electronics, high-strain actuators, and bio-integrated devices operating under mechanical extremes.

Beyond the design of chain viscosity and length in highly entangled and slide-ring hydrogels, recent advances in macromolecular topology have opened promising avenues for constructing novel high-performance hydrogel networks. In the realm of discrete molecular topology, four primary classes have been identified: branched structures, multicyclic structures, knots, and links [39]. Branched topologies, particularly hyperbranched polymers, offer unique advantages for hydrogel reinforcement due to their three-dimensional architecture, highly branched backbones, and multifunctional end groups. These features enhance chain flexibility, network uniformity, and crosslinking efficiency, leading to mechanically robust and highly tunable materials. For example, Jiang et al. [130] synthesized macromolecular crosslinkers using carboxyl-functionalized, acrylate-terminated hyperbranched polycaprolactone (PCL) to fabricate super-strong and tough ionic hydrogels. Meanwhile, dynamic non-covalent interactions, including Fe^3+^-COO^−^ coordination bonds and hydrophobic domain aggregation of PCL, introduced energy-dissipating mechanisms that further enhanced toughness and durability.

While topological hydrogels (e.g., highly entangled and slide-ring structures) show great promise in mechanical performance, their translation from the laboratory to practical use faces significant hurdles. The precise control of crosslinking density to balance strength and flexibility remains a primary challenge, especially in complex, multi-step synthesis systems. Furthermore, the material’s performance is highly sensitive to its hydration state; variations in environmental humidity or excessive swelling can compromise network integrity, leading to unstable mechanical properties under dynamic loading. Additionally, intricate synthesis processes, stringent reaction conditions, and high costs severely limit scalable production and batch-to-batch consistency.

### Hard–Soft Phase Structure Theory

The hard–soft phase structure theory is a relatively recent design concept introduced in the year of 2020, originating from studies on polyampholyte (PA) hydrogels that demonstrated exceptional toughness through phase-separated, bicontinuous network architectures [131]. This theory is based on the principle of mechanical synergy between a rigid, energy-dissipative “hard phase” and a ductile, load-absorbing “soft phase.” It can be regarded as a specific and advanced manifestation of the sacrificial bond theory at the microscale phase separation level, while also incorporating the design philosophy of structural synergy.

In this theory, the “hard phase” (such as ionic clusters or crystalline regions) essentially acts as high-strength, nanoscale “sacrificial regions,” whose rupture dissipates energy. Meanwhile, the “soft phase” provides a continuous, deformable matrix that transmits loads and accommodates deformation. However, its uniqueness lies in emphasizing the regulation of phase separation thermodynamics and kinetics to construct long-term stable, interpenetrating bicontinuous phase structures. Such structures enable synergistic crack resistance and multi-level energy dissipation, surpassing designs relying on single-type sacrificial bonds.

Mechanistically, the hard phase is typically constructed from strongly physically crosslinked domains, including crystalline regions [121], glassy polymer cores [132], or self-assembled helical structures [48]. These elements act as quasi-permanent crosslinks, offering mechanical rigidity and dimensional stability under high stress. The hard–soft phase design approach is highly adaptable and has been applied to various hydrogel systems to enhance their mechanical robustness, fatigue resistance, and fracture energy.

#### PA Hydrogel

Poly(amphoteric electrolyte) (PA) hydrogels are formed through random copolymerization of oppositely charged monomers, resulting in polymer chains bearing alternating polycationic and polyanionic units. These ionic groups interact via inter- and intra-chain electrostatic complexation, giving rise to heterogeneous ionic clusters that vary widely in both size and binding strength. This structural inhomogeneity forms the basis for the hard–soft phase separation that defines the mechanical behavior of PA hydrogels. Within the hydrogel network, ionic clusters are categorized based on the number and strength of ion-pair interactions. Large clusters, composed of hundreds of ion pairs, function as hard phases, i.e., rigid domains that act as quasi-permanent physical crosslinks. These domains provide structural reinforcement and serve as primary obstacles to crack propagation. In contrast, smaller clusters, consisting of a dozen or fewer ion pairs, constitute the soft phases, which behave more flexibly and allow for reversible deformation under load. This dual-phase structure enables the hydrogel to exhibit mechanical synergy: Under external stress, the hard domains resist deformation and dissipate high-energy impacts, while the soft domains accommodate strain and reversibly reorganize to prevent catastrophic failure. As a result, PA hydrogels display excellent toughness, extensibility, and fatigue resistance, even under complex and dynamic mechanical environments.

**Fig. 6:**
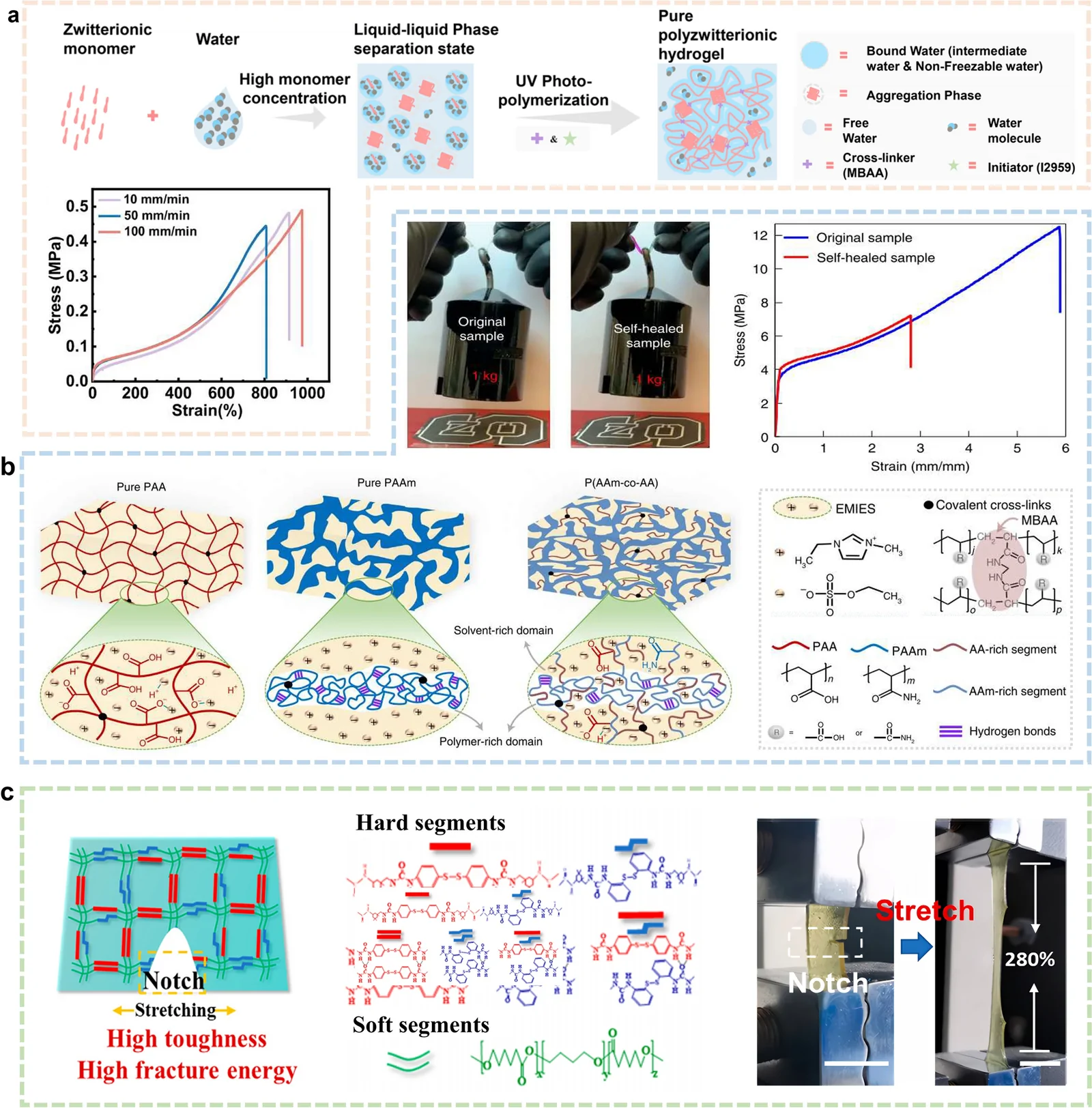
Representative materials illustrating the hard–soft phase structure theory in hydrogel and elastomer systems. **a** Poly(amphoteric electrolyte) hydrogel, exhibiting microphase-separated hard and soft domains, where hard phases provide compressive strength and rigidity, and soft phases enable toughness and recovery. **b** Ionic liquid hydrogel with a double-continuous phase structure composed of AAm-rich hard segments (via hydrogen bonding) and AAc-rich soft solvent domains, enhancing strength, toughness, and self-healing behavior. **c** Elastomer system comprising hard segments formed by strong hydrogen bonding (amino and carbonyl groups) and soft segments from polyester/polyether copolymers, offering flexibility, energy dissipation, and biodegradability. Reproduced with permission from [133, 134, 136]

One of the major advantages of PA hydrogels is their simplicity of fabrication. They can be synthesized through free-radical copolymerization of cationic and anionic monomers at optimized ratios, without the need for post-processing or complex structural templating. In the studies by Li et al. [131] and Yin et al. [133], the concept of hard–soft phase structuring was employed to simultaneously enhance the mechanical strength, energy dissipation, and self-healing capabilities of hydrogels through different material design routes. Briefly, Li et al. [131] synthesized PA hydrogels by chemically crosslinking oppositely charged monomers of sodium polystyrene sulfonate (NaSS) and chloromethyl-quaternary *N*,*N*-dimethylaminoethyl methacrylate (DMAEA-Q). Upon removal of the small counterions (Na^+^ and Cl^−^), strong ionic bonds spontaneously formed between oppositely charged polymer chains, driving local ionic aggregation and inducing microphase separation into hard and soft domains. The hard phase, composed of densely packed, high-strength ionic clusters, contributed significantly to the gel’s rigidity and mechanical integrity, while the soft phase, formed by weaker and more reversible ionic interactions, served as a dissipative domain that could undergo breakage and reformation under stress, enhancing the material’s toughness and flexibility. In a complementary approach, Yin et al. [133] developed a high-strength, high-toughness zwitterionic hydrogel based on poly(sulfobetaine methacrylate) (PSBMA) via the synergistic combination of liquid–liquid phase separation and polymer chain entanglement (Fig. 6a). This hydrogel similarly formed hard–soft phase-separated domains, with the hard phase imparting high compressive strength and dimensional stability, and the soft phase contributing to strain adaptability, toughness, and recovery dynamics. Despite differences in chemistry and phase formation mechanisms, both studies highlight the crucial mechanical synergy achieved through the coexistence of hard and soft phases. The hard domains, formed via strong ionic bonding or phase-separated dense networks, provide structural reinforcement and crack resistance, while the soft domains, consisting of flexible, reversibly interacting segments, enable energy dissipation, self-repair, and stress redistribution under mechanical loading.

As a toughening strategy for hydrogels, polyelectrolyte-based hydrogels do have certain limitations in raw material selection and preparation. In terms of raw material selection, the high hydration of zwitterionic groups in these hydrogels leads to weak inter-chain interactions, resulting in limited mechanical properties. Additionally, traditional zwitterionic hydrogels are difficult to achieve injectability, limiting their use in dynamic environments. To address these issues, researchers have explored methods such as introducing dynamic crosslinks, hybrid materials, or elastic polymers to enhance toughness while maintaining biocompatibility and flexibility. Beyond material selection and preparation, zwitterionic hydrogels also face limitations in other aspects of toughening strategies. For instance, their toughening mechanism primarily relies on the breaking and reformation of ionic bonds, which may fail under extreme conditions (e.g., high temperature, high humidity), leading to irreversible degradation of material properties. Additionally, zwitterionic hydrogels undergo irreversible softening after large deformations, which limits their use in applications requiring high stability and long-term performance. Therefore, despite their excellent toughening capabilities, zwitterionic hydrogels still require further research and optimization to overcome these challenges in practical applications.

#### Other Hydrogels

The hard–soft phase concept has been successfully extended beyond conventional hydrogels to a broad range of ionic liquid gels, organic hydrogels, and elastomers, enabling the design of mechanically robust, adaptable, and multifunctional materials. For instance, Wang et al. [134] enhanced ionic liquid P(AAm-co-AAc) hydrogels by engineering a double-continuous phase structure composed of hard AAm-rich domains (stabilized by hydrogen bonding) and soft AAc-rich solvent domains (Fig. 6b). This architecture significantly improved the gel’s tensile strength, toughness, and self-healing capability. Physically, the homogeneous distribution of ionic liquids facilitated stress dispersion, inhibited crack formation, and enhanced long-term durability. Chemically, the ionic liquids reacted with functional groups in the polymer network to promote crosslinking or structural rearrangement, further boosting mechanical performance. Similarly, Zhou et al. [135] developed double-crosslinked PVA-PI organic hydrogels, in which the hard phase was composed of PVA crystalline domains and covalent crosslinking points, while the soft phase consisted of poly(imide) (PI) chains that imparted flexibility and extensibility. In this system, the hard domains benefit from solvent-induced crystallization and uniform crosslinking, while the soft domains gain enhanced fluidity and ductility, collectively contributing to a highly durable and adaptable gel network. In the domain of elastomers, Xun et al. [136] achieved substantial improvements in self-healing and mechanical performance of the TDSE elastomer by tailoring interactions between hard segments formed through strong hydrogen bonding between amino and carbonyl groups and soft segments comprising polyester/polyether copolymers (Fig. 6c). This optimized distribution of hard and soft domains enabled effective energy dissipation under mechanical stress while maintaining elasticity, flexibility, and biodegradability.

In the design of microphase-separated hydrogels, researchers commonly employ the hard–soft phase structure as a key concept to construct heterogeneous networks, synergistically enhancing mechanical properties. By integrating sacrificial bonding mechanisms with hard and soft microdomains, such hydrogels can effectively resist fracture, dissipate energy efficiently, and maintain structural integrity under large deformations. The reinforcement and toughening mechanisms primarily stem from the formation of discrete hard domains—typically composed of crystalline, glassy, or highly crosslinked segments embedded within a compliant soft matrix. Under mechanical loading, the soft phase absorbs and dissipates energy through reversible interactions or chain rearrangements, while the hard phase bears the load and suppresses crack propagation. Their synergistic interaction endows the material with outstanding overall performance. It is worth noting that the hard–soft phase structure, as a prevalent design strategy, is not limited to typical hydrogel systems but also extends to various polymer materials such as ionic liquid gels, organic gels, and elastomers, where it helps balance strength, flexibility, and functional responsiveness. In later sections of this paper, we will further discuss preparation methods for microphase separation, where the concept of hard–soft phase structure will be illustrated in concrete experimental design and structural control.

### Self-Growth Theory

Self-growth hydrogels represent a novel class of dynamic materials capable of autonomously increasing in volume, structure, or network complexity under specific external stimuli or internal chemical triggers. Analogous to biological growth, these hydrogels exhibit expansion through mechanisms such as water absorption, monomer polymerization, or mechanically induced chemical reactions. Critically, the core of self-growth lies in stimulus-triggered in situ chemical reactions (e.g., mechanoradical polymerization, interface-initiated polymerization, catalytic crosslinking), rather than simple physical swelling. Environmental conditions (e.g., pH, temperature, ionic strength) act as essential “triggers” or “modulators” for these processes, dictating their initiation, kinetics, and outcome. This growth behavior not only expands the spatial footprint of the hydrogel but also enhances its mechanical properties, adaptability, and longevity.

A pioneering example was introduced by Gong et al. in 2019 [28], who developed a mechanically activated self-growth double-network hydrogel system. The hydrogel consisted of a brittle primary network formed from poly (2-acrylamido-2-methylpropanesulfonic acid sodium salt) (PNaAMPS) and a ductile secondary network based on poly(acrylamide) (PAAm). Upon application of mechanical force, the brittle PNaAMPS network undergoes bond scission, generating free radicals that trigger the in situ polymerization of monomers in the surrounding medium. Simultaneously, the extensible PAAm network maintains the overall structural integrity, allowing the material to grow internally while remaining functionally intact. This mechanoradical polymerization mechanism enables the hydrogel to “self-grow” in both mass and complexity in response to mechanical stimuli. Such behavior provides a feedback loop between mechanical damage and structural reinforcement, endowing the material with autonomous adaptability, prolonged service life, and potential self-repair capabilities. The self-growth theory introduces a new paradigm in hydrogel design, moving beyond passive structural resilience toward active mechanical regeneration and programmable growth. It holds great promise for applications in tissue engineering, regenerative soft robotics, and adaptive biointerfaces, where materials must respond dynamically to stress, wear, or environmental change.

**Fig. 7:**
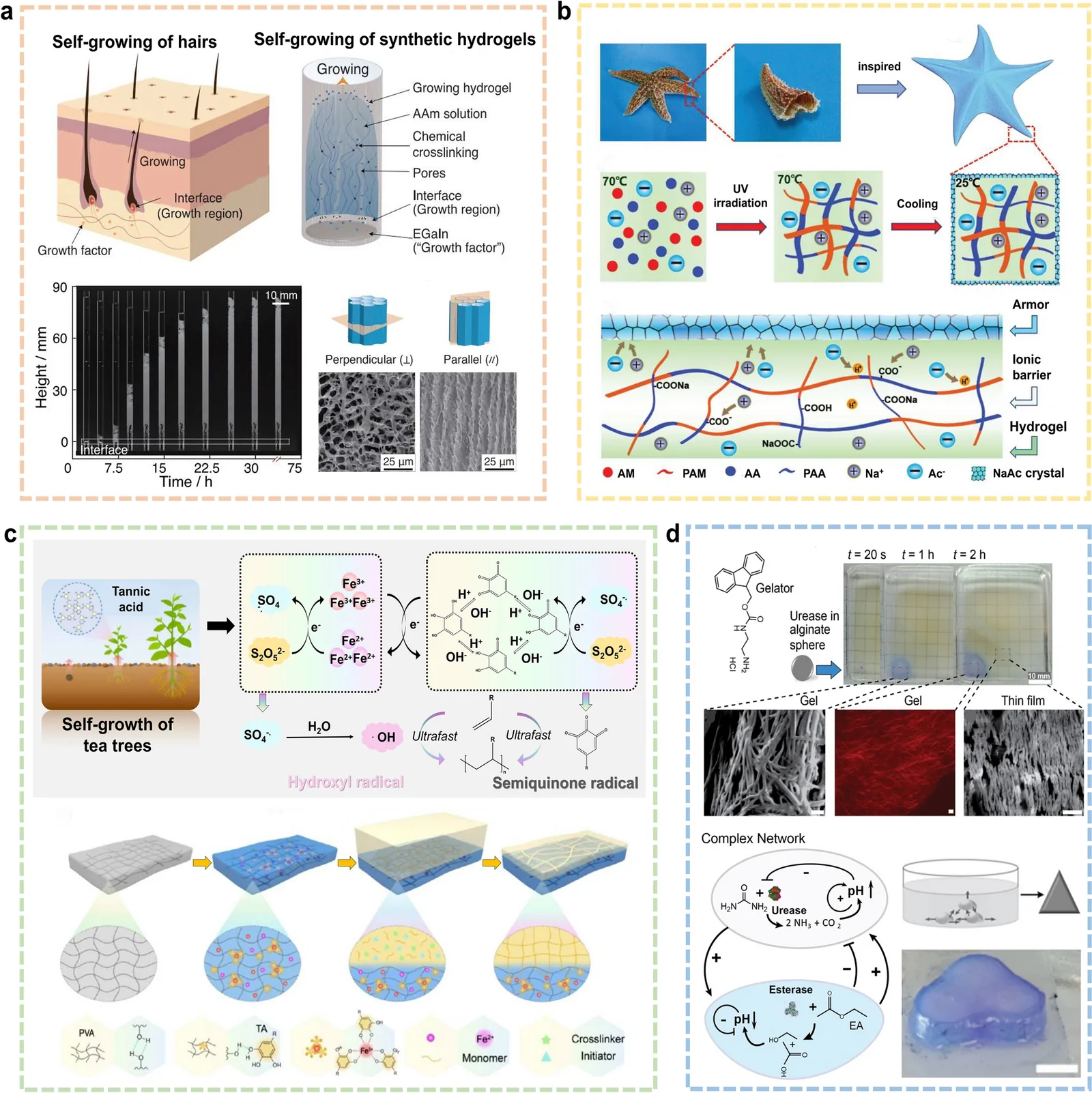
Representative systems illustrating the self-growth theory in hydrogel materials. **a** Bioinspired self-growth hydrogel initiated by liquid metal (EGaIn), which triggers free-radical polymerization to mimic keratin-like growth under mechanical stimulation. **b** Sodium acetate crystallization on the hydrogel surface forms a self-growing protective layer, capable of autonomous repair after damage. **c** Autocatalytic hydrogel matrix based on plant polyphenol-Fe.^3+^ complexes, enabling monomer incorporation and network expansion via self-catalyzed reactions. **d** Urease-catalyzed hydrogel system guiding morphological evolution of hydrogel spheres in response to environmental stimuli. Reproduced with permission from [43, 137–139]

In nature, living organisms possess the remarkable ability to grow, repair, and evolve complex architectures without continuous external energy input. Inspired by these biological principles, recent research has demonstrated that self-growth hydrogels can be designed to autonomously expand, reinforce, or morphologically adapt in response to environmental or mechanical stimuli, offering exciting prospects for next-generation smart materials. For instance, Jian et al. [43] developed a self-growth double-network hydrogel in which new polymer networks are formed via radical polymerization at the interface between liquid metal (eutectic gallium indium, EGaIn) and an acrylamide (AAm) precursor solution (Fig. 7a). Under mechanical activation, the hydrogel continuously expands its network, mimicking the dynamic growth observed in biological tissues. In a complementary strategy, Zhao et al. [137] fabricated a hydrogel capable of forming a self-growing protective crystalline layer through surface crystallization of sodium acetate (Fig. 7b). This protective shell not only self-repairs upon damage but also enhances the mechanical durability of the hydrogel, offering potential for harsh-environment applications. Wei et al. [138] proposed a self-catalyzing hydrogel system, where plant-derived polyphenols complexed with Fe^3+^ ions enable the hydrogel matrix to incorporate new monomers via catalytic reactions (Fig. 7c). This chemical design allows the material to regenerate and expand its internal network in situ, enabling both structural renewal and functional enhancement. Further extending the concept, Maity et al. [139] utilized urease-catalyzed reactions to direct the growth of hydrogel spheres, allowing the fabrication of complex 3D hydrogel geometries with high precision (Fig. 7d).

The study of self-growth hydrogels, although initiated relatively recently, has developed rapidly in recent years, particularly in the fields of biomimetic material design and smart-responsive materials. These investigations have not only expanded the application scope of hydrogels but also provided new insights for the design of high-performance hydrogels in the future. However, due to the complexity of the preparation process, which involves the synergistic interaction of multiple chemical and physical mechanisms, current research remains largely at the laboratory scale and has not yet established a mature industrial application system. Self-growth hydrogels exhibit irreversible softening after large deformations. Although the introduction of sacrificial bonds or dissipative structures can enhance the self-healing capability of the material, their recovery capacity under continuous cyclic loading remains limited. Furthermore, the stability of self-growth hydrogels under extreme conditions, such as high temperature, high humidity, or strong mechanical stress, is also a concern. A pivotal, unresolved question is whether the self-growth mechanism, over many cycles, leads to a more homogeneous and robust network, or if it inadvertently introduces compositional drift and structural heterogeneity that ultimately undermine the long-term performance. As an emerging method for toughening hydrogels, self-growth hydrogels hold significant potential, but their research is still in a developmental stage and faces certain limitations. Future studies should concentrate on gaining a deeper understanding of their toughening mechanisms and exploring more efficient preparation methods and broader application scenarios.

## Further Toughing Strategies

The growing application of hydrogels in fields such as biomedicine, soft robotics, and wearable sensors has driven a surge of research focused on improving their mechanical strength, durability, and multifunctionality. To meet these demands, recent strategies have increasingly drawn inspiration from nature’s high-performance structural materials, such as spider silk, silkworm silk, tendon, wood, shells, and corals, all of which exhibit exceptional combinations of toughness, flexibility, and hierarchical architecture. In addition to by leveraging these biomimetic design principles, researchers have implemented various approaches to enhance hydrogel performance, including: (1) externally induced microphase separation, (2) temperature-regulated or solvent-mediated phase reorganization, and (3) advanced fabrication techniques, such as 3D printing, which enables precise spatial control of material composition and architecture. This section will explore the biomimetic inspirations, phase separation techniques, and emerging fabrication methods that are shaping the development of next-generation hydrogels engineered to perform under mechanical extremes and dynamic functional demands.

### Bioinspired Structural Design

Nature provides powerful design cues through hierarchically organized biological structures, such as spider silk, silk fibers, and plant tissues, which exhibit exceptional combinations of mechanical toughness, extensibility, and energy dissipation. Unlike traditional hydrogels with randomly distributed polymer networks, these natural materials leverage ordered, multiscale architectures to achieve superior mechanical performance. This has led to the emergence of bioinspired ordered-structure hydrogels, which aim to replicate the mechanical strategies of natural soft tissues, such as high tensile strength, enhanced fracture toughness, and superior fatigue resistance, along with intelligent stimuli responsiveness. This section explores key biological archetypes, such as protein-based fibers (e.g., spider silk, silk), plant-derived architectures (e.g., wood, lotus fibers), and human tissue analogues (e.g., skin, ligaments), to highlight recent advances in mechanically adaptive hydrogel design. By analyzing how biological systems achieve multiscale toughness and durability, we provide a conceptual and practical framework for the next generation of hydrogels tailored for use in biomedicine, soft robotics, and advanced structural materials.

#### Spider Silk/Worm Silk

Spider silk and silkworm silk are renowned for their exceptional mechanical properties, which arise from their hierarchical structural organization, molecular alignment, and controlled self-assembly processes. These natural protein fibers serve as biomimetic models for designing tough, strong, and resilient hydrogels, offering insights into how multiscale architectures can be leveraged to achieve mechanical extremes [130]. From the nanoscale β-sheet crystalline domains to micron-scale fibrillar assemblies, silk materials exhibit a layered hierarchical structure that enables effective stress distribution across multiple length scales [140, 141]. This architecture not only enhances energy dissipation and fracture resistance but also supports dynamic adaptability in response to external stimuli.

**Fig. 8:**
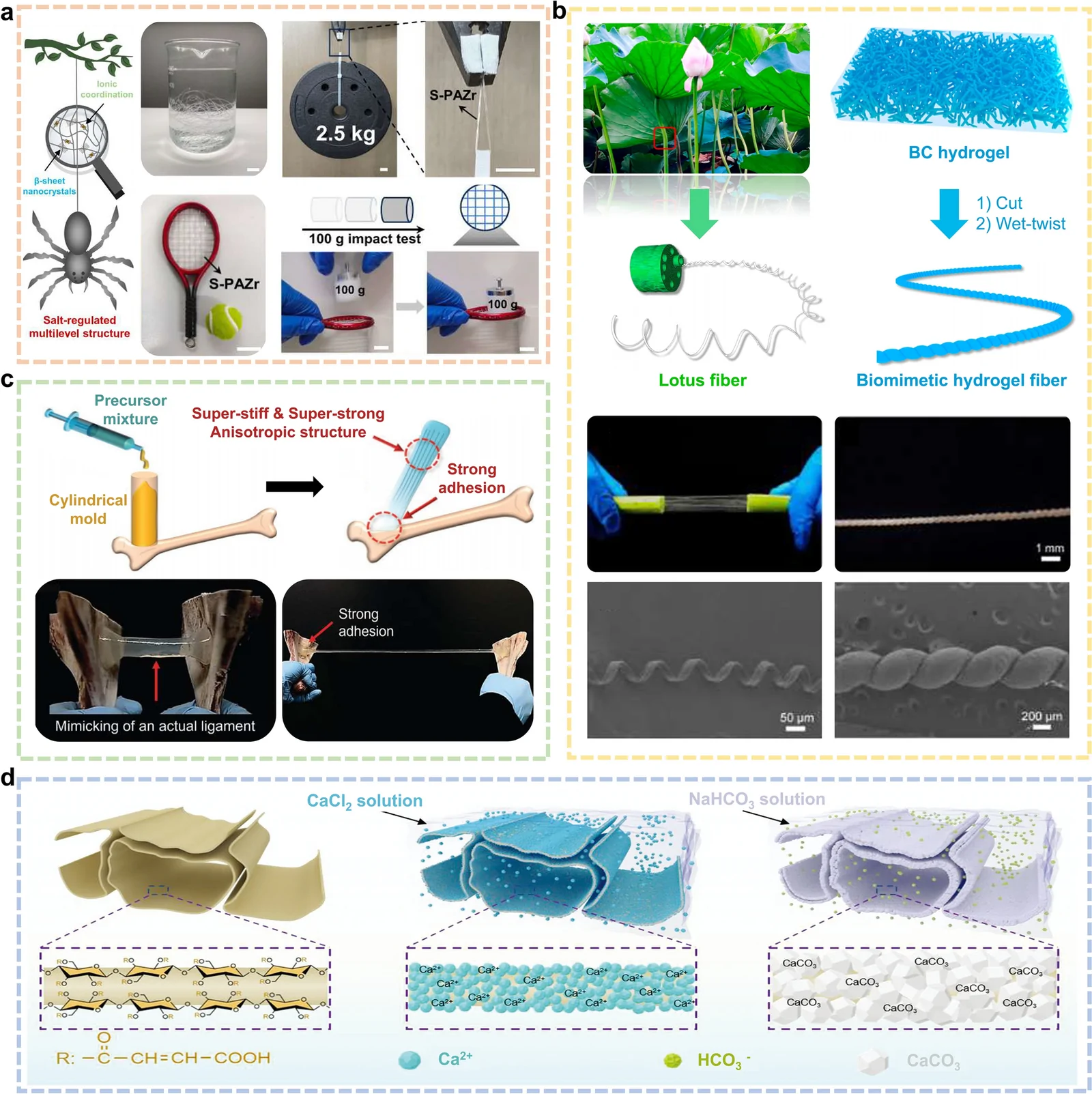
Biomimetic strategies inspired by natural materials for enhancing the mechanical properties of hydrogels. **a** Spider silk-inspired design: Mimicking the *β*-sheet nanocrystal and amorphous domain hierarchy to improve strength and toughness. **b** Lotus root fiber-inspired helical structure: Tangentially deformed bacterial cellulose hydrogel replicating spiral morphology for enhanced stretchability and crack resistance. **c** Tendon-inspired triple-network hydrogel: Anisotropic alignment and interfacial adhesion mimicking hierarchical soft tissues for robust energy dissipation and fatigue resistance. **d** Pearl formation-inspired mineralization: Alternating deposition of calcium carbonate on wood scaffolds to create layered mineral-polymer composites with improved mechanical integrity. Reproduced with permission from [142, 149, 154, 156]

Inspired by these natural structures, researchers have developed various strategies to mimic the mechanical behavior of silk. Liu et al. [142] engineered a nanoconfined hydrogel using polyvinyl alcohol to replicate amorphous protein domains and aramid nanofibers to mimic β-sheet nanocrystals. This biomimetic design achieves simultaneous high tensile strength (2.07 MPa), extensibility (1084%), toughness (12.66 MJ m^−3^), fracture energy (3196 J m^−2^), and fatigue threshold (157 J m^−2^), offering a robust platform for soft robotics and artificial tendons (Fig. 8a). This natural paradigm inspires bioinspired hydrogel fabrication where tailored temperature, pH, and ionic strength direct microstructural organization for enhanced mechanical performance [143]. Mimicking silkworm silk’s solution spinning and phase separation, Lu et al. [144] fabricated sericin-based ionic hydrogel fibers with semicrystalline alignment via continuous wet-spinning, achieving 55 MPa tensile strength, 530% ductility, and 0.45 S m^−1^ conductivity for flexible transparent electrodes. In spider silk spinning, inorganic salt ions also play a key role by inducing protein aggregation, stabilizing crystalline domains, and modulating mechanical behavior [145]. Inspired by salt-regulated spider silk spinning, Wu et al. [146] developed a biomimetic spinning-structure-environment strategy where ionic crosslinking and crystalline domains synergistically impart 162.25 MJ m^−3^ toughness, with Young’s modulus tunable from 0.27 to 118.53 MPa. These fibers maintain 0.0014 mS cm^-1^ conductivity at − 40 °C, exceed 50 MPa strength with 200% elongation.

The design of bioinspired hydrogels often involves multiscale engineering, such as aligning polymer chains through air-drying or freeze-casting techniques to form hierarchical structures from nanoscale to microscale. These methods not only enhance mechanical properties but also provide a generalizable framework for designing ordered hydrogels. By understanding and emulating these principles, researchers can develop materials with superior mechanical and functional properties for a wide range of applications.

#### Wood/lotus Fibers

Wood exhibits multiscale hierarchical architecture spanning macroscopic trunks/growth rings, cellular wall layers (including middle lamella and secondary walls), and microscale crystalline microfibrils ordered within lignocellulosic matrices. This anisotropic composite, formed through sequential cellulose/lignin deposition during growth, delivers exceptional compressive strength, crack propagation resistance, and mechanical resilience.

In situ self-assembly of cellulose into crystalline microfibrils within lignin–hemicellulose matrices creates laminated anisotropic composites. This growth-coupled consolidation process confers inherent compressive strength (~ 60 MPa), crack deflection capacity, and deformation energy dissipation to wood [147]. Mimicking wood’s anisotropic toughening mechanism, Wu et al. [148] engineered PVA/CNF hydrogels via salt-induced phase separation coupled with pre-stress alignment, bio-replicating hierarchical cellulose-lignin architecture. Achieving breakthrough tensile strength (> 40 MPa), elongation (> 250%), and toughness (61.8 MJ m^−3^). Guan et al. [149] engineered spiral hydrogel fibers via tangential-force-induced hydrogen bond reconfiguration in bacterial cellulose, achieving 116.3 MJ m^−3^ toughness (9 × untreated fibers) and > 90 MPa tensile strength, and controllable deformation recovery for revolutionary surgical sutures (Fig. 8b). The structural features of both wood and lotus fibers demonstrate the optimization of structure–function relationships, emphasizing the critical role of microstructural alignment, phase separation, and geometric configuration in improving material performance.

#### Human Soft Tissues and Muscle Ligaments

Human soft tissues exhibit unique mechanical behavior through multiscale hierarchical architecture, anisotropy, and high-water content (70–90%). The epidermal–dermal–hypodermal tri-layer system exemplifies protection–elasticity–energy dissipation synergy, offering fundamental biomimetic principles for tough adaptive hydrogels [150]. Inspired by skin’s multiscale anisotropy, Fu et al. [151] applied directional freezing to enhance toughness parallel to freezing while achieving high responsiveness perpendicularly. This architecture replicates dermal mechanical gradients with directional functionality. Such biomimetic systems enable tough-yet-compliant solutions for artificial ligaments and muscle actuators, advancing bio-integrated applications.

Moreover, inspired by the highly organized collagen fiber alignment in human soft tissues, often in parallel or staggered configurations enabling simultaneous high tensile strength and elasticity, bioinspired designs can emulate this structural [152]. Emulating the oriented collagen fiber architecture of natural skin to boost mechanical resilience, the PVA-Gp/TA-CaCl_2_ hydrogel attains 5.79 MPa tensile strength and high toughness via salt-assisted freeze–thaw crosslinking, while integrating conductivity, UV-blocking capability, and recyclability for multifunctional e-skin systems [153]. Inspired by the hierarchical dissipative architecture of tendon tissue, Choi et al.’s triple-network hydrogel integrates ionic/covalent/supramolecular interactions, achieving tendon-like anisotropy via stretch-mediated remodeling (Fig. 8c) [154].

#### Underwater Creature

In recent years, the structural ingenuity of marine organisms has offered a powerful biomimetic framework for the design of strong and tough hydrogels. Marine organisms offer biomimetic paradigms for tough hydrogel design through stratified soft–hard architectures, where alternating phase interfaces enhance compressive/fracture resistance via crack-arresting and energy dissipation [155]. Their layered composite materials combine calcium-based minerals with organic polymers, enabling the hydrogel to achieve synergistic stiffness and toughness.

One of the most iconic biomineralization processes in nature is the formation of pearls, wherein calcium carbonate is gradually deposited onto an organic matrix scaffold, forming a smooth, compact, and highly durable structure. Mimicking this mechanism, Qiu et al. [156] developed a bioinspired mineralized scaffold by alternately immersing wood substrates in calcium chloride (CaCl_2_) and sodium bicarbonate (NaHCO_3_) solutions (Fig. 8d). Nacre-wood bionic coupling via alternating mineralization yields millimeter-thick artificial nacre with 93.31 MPa bending strength, 122.59 MPa tensile strength, 7.40 MPa m^1/2^ fracture toughness, and 4.61 MJ m^−3^ work of fracture, whose 1.59 g cm^−3^ density enables specific strength surpassing natural nacre and analogs for structural engineering. Biological core–shell architectures synergize rigid exteriors for high hardness/compressive strength with deformable energy-absorbing cores, enabling materials to achieve impact resistance, structural protection, and damage tolerance under complex loading. Mimicking the rigid–ductile synergy of marine shells and cortical bone, Xu et al.’s core–shell hydrogel integrates stiff protective exteriors with dissipative cores to achieve extreme mechanical properties: 55.3 MPa compressive strength, 1031 MJ m^−3^ toughness, and 40.9 kJ m^−2^ fatigue threshold, positioning it as an ideal platform for impact-absorbing biomaterials and adaptive composites [157]. The hollow porous architecture of coral skeletons synergistically provides mechanical stability and high surface area, enabling efficient mass/heat transfer and environmental interactions in biological-aqueous systems. Liu et al. [158] developed coral-channel-inspired hollow microneedles that synergize wound exudate absorption with pathogen-responsive drug release, where therapeutic delivery automatically adjusts to infection severity while enabling real-time monitoring.

### Microphase Separation

#### External Force-Driven Orientation

**Fig. 9:**
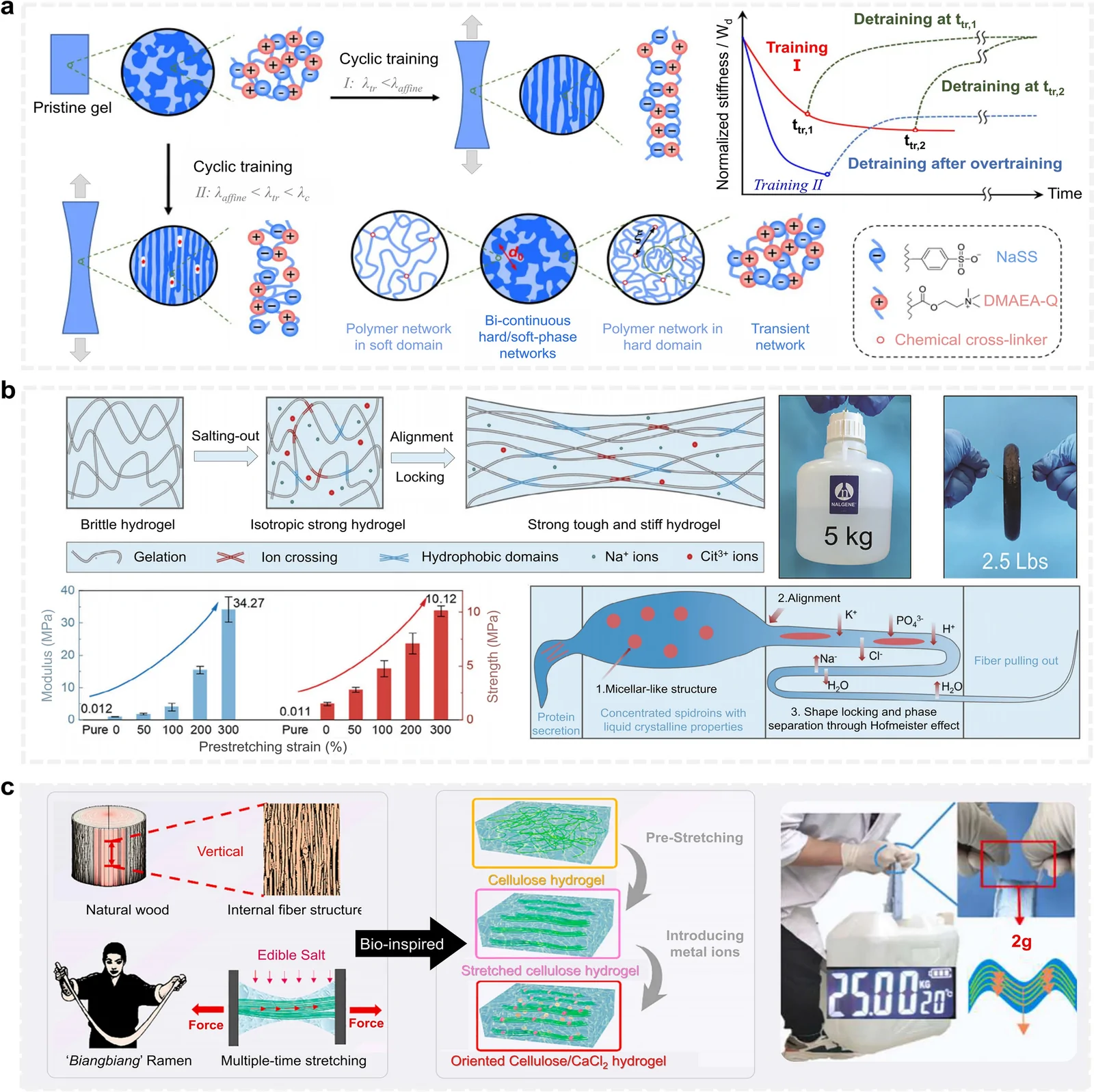
Mechanical external force-induced strategies for enhancing the mechanical properties of hydrogels. **a** Cyclic stretching training improves the stress–strain behavior and fatigue resistance of polyelectrolyte hydrogels by inducing dynamic chain reorganization and microphase adjustment. **b** Pre-stretching combined with salting-out effects locks anisotropic chain orientation, mimicking the hierarchical structure of spider silk and dramatically enhancing mechanical performance. **c** Metal ion coordination-reinforced cellulose-based hydrogel, formed by pre-stretching the initial network followed by metal ion infusion, exhibits significantly increased strength and fracture toughness. Reproduced with permission from [159–162]

External mechanical forces during hydrogel fabrication induce microphase separation and polymer chain alignment, enhancing anisotropy to improve stress distribution, energy dissipation, and resistance to mechanical degradation. For example, Li et al. [159] utilized cyclic stretching to induce progressive reorganization and alignment of polymer chains in polyelectrolyte hydrogels, significantly enhancing mechanical energy dissipation, fatigue resistance, and mechanical durability (Fig. 9a). Similarly, Liu et al. [160] employed mechanical training to transform κ-carrageenan hydrogels into ordered rigid structures, achieving 396% fracture strain, 0.55 MPa tensile strength, and 1.52 MJ m^−3^ toughness while enhancing crystallinity and anisotropy.

By physically deforming the hydrogel during or after synthesis, pre-stretching promotes fiber orientation, crystallinity, and intermolecular interactions, thereby significantly improving strength, toughness, and durability, especially under dynamic loading conditions. In a representative study, Sun et al. [161] proposed a “salt-induced coordination-locking” strategy that combines pre-stretching with salting-out effects to mimic the hierarchical nanostructure of spider silk (Fig. 9b). The hydrogel was first mechanically stretched to orient its internal network and then stabilized by salt-induced locking, effectively “freezing” the anisotropic architecture. This approach led to dramatic enhancements in mechanical performance, with increases in tensile stress, toughness, and modulus by approximately 940-, 1075-, and 2830-fold, respectively. Similarly, Jing et al. [162] employed pre-stretching combined with metal coordination bonds to align cellulose microfibers and reinforce the network, achieving a tensile strength of 52.79 MPa, Young’s modulus of 287 MPa, and fracture toughness of 34.41 MJ m^−3^ for enhanced load-bearing performance (Fig. 9c).

#### Solvent Immersion

As a critical fabrication strategy for hydrogel toughening, solvent immersion leverages diffusion-driven exchange to remove unreacted monomers and by-products, enhancing network purity and structural uniformity while strengthening mechanical properties across diverse media. By precisely modulating crosslinking density and inducing microphase rearrangement, this technique enables organic solvents to trigger polymer densification, and salt/metal ion solutions to activate ionic crosslinking and coordination reinforcement, thereby concurrently enhancing strength, stiffness, and swelling stability (Table 4). Furthermore, its capacity for functional dopant incorporation establishes a versatile platform for developing smart hydrogels with integrated sensing, actuation, and self-healing capabilities.

**Table 4:** Impact of four solvent immersion strategies on hydrogel toughness and functionality

Types	Solvent	Aspect	Effects and Pros/Cons
Water Immersion	Water	Toughness	Pros: High extensibility (swollen state); Cons: Lowest strength/modulus, extreme brittleness below 0 °C; Mechanism: No efficient energy dissipation
		Functionality	Pros: Optimal biocompatibility, high conductivity, transparency; Cons: Rapid dehydration, microbial growth, no freeze-resistance
		Key Limitation	Poor mechanical properties, low environmental stability
		Applications	Cell culture, biosensors, drug delivery carriers
Organic Solvent Immersion	Glycerol, DMSO, Formic acid, etc.	Toughness	Pros: Drastically toughness (plasticization + energy dissipation), cryogenic flexibility (-50 °C); Cons: Potential strength reduction at high concentrations
		Functionality	Pros: Superior freeze/desiccation resistance, mild antimicrobial effect (glycerol); Cons: Biocompatibility (DMSO/formic acid toxicity), conductivity
		Key Advantage	Top strategy for toughening, extreme environmental stability
		Applications	Cryogenic flexible electronics, soft robotics, cryopreservation
Metal Ion Immersion	Ca^2^⁺, Fe^3^⁺, Al^3^⁺, etc.	Toughness	Pros: Ultimate strength/modulus (coordination crosslinks), toughness via dynamic bonds; Cons: Drastic extensibility, brittleness from over-crosslinking
		Functionality	Pros: Enables magnetism (Fe^3^⁺), self-healing, antimicrobiality (Ag⁺/Zn^2^⁺); Cons: Cytotoxicity risk (heavy metals), transparency
		Key Advantage	Most significant mechanical enhancement, multifunctionality
		Applications	Self-healing hydrogels, magnetic actuators, antibacterial wound dressings
Salt Solution Immersion	NaCl, NaSO_4_, KCl, etc.	Toughness	Pros: Strength/modulus (“salting-out”), improved low-temperature flexibility; Cons: Limited toughness enhancement, extensibility
		Functionality	Pros: Highest ionic conductivity, cost-effective freeze-resistance (freezing point); Cons: Hyperosmotic stress to cells, long-term instability (salt crystallization)
		Key Advantage	Optimal conductivity, low-cost cryoprotection
		Applications	Flexible conductors, electrolyte membranes, low-cost cryomaterials

**Fig. 10:**
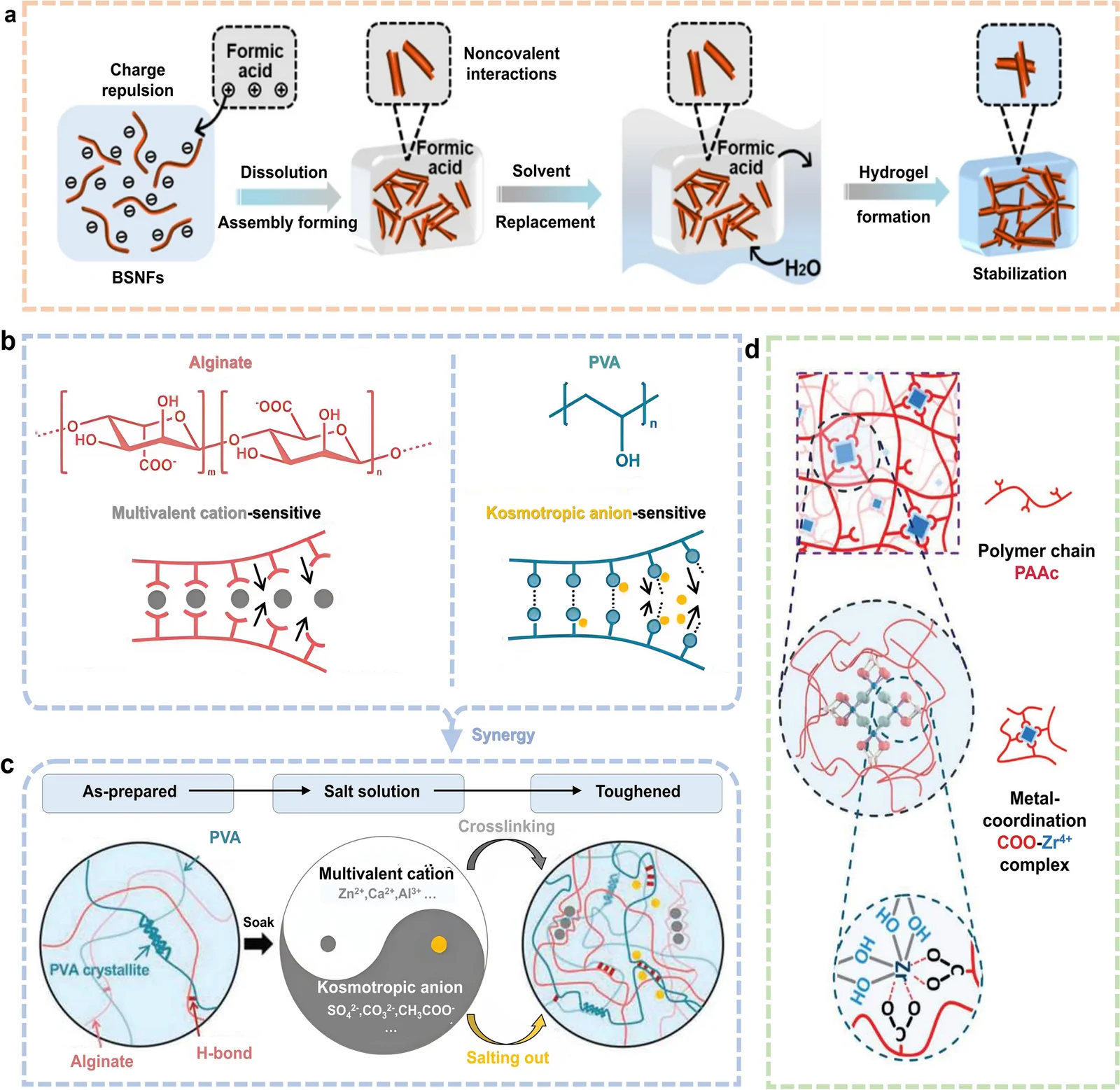
Solvent immersion strategies for tuning hydrogel structure and mechanical properties. **a** Organic solvent treatment: DMSO regulates polymer chain conformation, enhancing crystallinity and electrochemical performance in hydrophilic salt-compatible PVA hydrogels. **b, c** Salt solution immersion: Ion diffusion induces crosslinking and microphase separation in physical double-network hydrogels, significantly improving tensile strength, fracture energy, and conductivity. **d** Metal ion coordination: Zr^4+^ ions form stable coordination bonds with sulfonate groups, enhancing mechanical strength and transparency of polyelectrolyte hydrogels. Reproduced with permission from [166, 168, 169]

In the pursuit of enhancing hydrogel mechanical performance, the organic solvent substitution strategy has emerged as a widely adopted and efficient post-treatment technique. Commonly employed organic solvents include glycerol [163], ethylene glycol [164], formic acid [165], and dimethyl sulfoxide (DMSO) [166]. Xu et al. [29] demonstrated that DMSO-to-water solvent exchange maintains extended PVA chain conformations while rebuilding crosslinks, significantly enhancing hydrogel toughness and anti-swelling properties. Zhang et al. [165] further employed formic acid/water exchange to reorganize nanofiber networks and elevate β-sheet content, yielding silk hydrogels with modulus 5.88 ± 0.82 MPa, tensile strength 1.55 ± 0.06 MPa, and toughness 0.85 ± 0.03 MJ m^−3^. Similarly, He et al. [166] engineered a salt-compatible PVA hydrogel electrolyte for zinc anode stabilization, utilizing DMSO as the substitution solvent. In DMSO, the PVA chains were maintained in an extended and evenly dispersed conformation, whereas in water, the chains adopted folded, aggregated structures (Fig. 10a). This electrolyte delivered exceptional performance in energy storage systems. A Zn/Zn symmetric pouch cell demonstrated stable cycling for over 1300 h at 1 mA cm^-2^, while a Zn/MnO_2_ full cell retained 98.4% capacity after 100 cycles, confirming the effectiveness of solvent-mediated structural control for electrochemical applications.

The mechanism of ion diffusion-induced microphase separation in salt solutions is fundamentally governed by the Hofmeister series, which describes the ion-specific regulation of salt-out and de-salting effects in polymer systems, particularly proteins. This principle has been broadly applied to various hydrogel-forming polymers. Salt ions, depending on their position in the Hofmeister series, influence polymer–solvent interactions, leading to phase reorganization, network densification, and enhanced mechanical and functional properties. In a systematic investigation, Wu et al. [167] achieved mechanically robust phase-separated gels via saturated sodium sulfate bath immersion, where sulfate ion-hydroxyl dipole interactions triggered chain aggregation to reinforce the matrix. In a related study, Cui et al. [168] investigated the role of salt immersion in tuning the properties of physically crosslinked double-network hydrogels (Fig. 10b, c). Upon immersion in selected salt solutions, the hydrogels underwent secondary crosslinking and salt crystallization, resulting in profound enhancements in both mechanical and conductive performance. After treatment, the hydrogels exhibited a tensile strength increase of 530-fold, fracture energy enhancement by 1100-fold, and a 4.9-fold rise in ionic conductivity, ultimately achieving a tensile strength of 15 MPa, fracture energy of 39 kJ m^−2^, and ionic conductivity of ~ 1.5 S m^−1^, valuing that surpass most conventional high-conductivity hydrogels.

Currently, by modulating the type, concentration, or coordination environment of metal ions and ligands in solution, researchers can precisely control the crosslinking density, enabling fine-tuning of both the mechanical performance and stimuli-responsive behavior of hydrogels. These metal ions form coordination networks or metal–organic frameworks (MOFs) within the hydrogel matrix, significantly enhancing structural integrity, toughness, and functional adaptability. For instance, in previous work by Zhen and Wu’s group [17], Zr^4+^ ions were shown to form stable coordination bonds with sulfonate groups, dramatically improving the mechanical robustness of sulfonated polyelectrolyte hydrogels. Expanding upon this, Yu et al. [169] developed high-transparency hydrogel films by initiating acrylic acid polymerization in the presence of Zr^4+^ (Fig. 10d). The resulting COO^−^-Zr^4+^ coordination bonds served as dynamic yet stable physical crosslinking sites, effectively enhancing the tensile strength, Young’s modulus, and elongation at break of the hydrogel films. Chen and Cui et al. [170] engineered mechanically robust fluorescent hydrogels (tensile strength: 3.9 MPa, Young’s modulus: 0.66 MPa) via synergistic lanthanide coordination/hydrogen bonding in solution. Precise tuning of Eu^3+^/Tb^3+^ ratios enabled emission-color control, while acid–base reversible coordination granted cycling-stable fluorescence for information encryption and optical sensing. These multivalent coordination interactions typically involve functional groups such as carboxyl (-COOH) [171], amine (-NH_2_) [172], and hydroxyl (-OH) groups [173], which are abundant in natural and synthetic polymer matrices. This modular crosslinking strategy enables tunable mechanics for applications including soft robotics, stretchable electronics, and injectable therapeutics.

#### Temperature-Induced Microphase Separation

Temperature-induced microphase separation is a widely employed and highly effective strategy for enhancing the mechanical strength, structural stability, and functional robustness of hydrogels. Precise regulation of thermal gradients during sol–gel transition induces thermodynamic phase separation within the polymer network. The core mechanism involves temperature-dependent changes in the solubility and mobility of polymer chains or fillers, leading to domain formation, pore alignment, and localized densification. Recently, temperature-induced phase separation has been synergistically combined with techniques like freeze-casting, cyclic freeze–thaw, and wet annealing to further tune network architecture and mechanical performance. This section outlines several representative methods based on temperature-induced phase separation and discusses their progress and applications in the preparation of strong, tough, and multifunctional hydrogels.

**Fig. 11:**
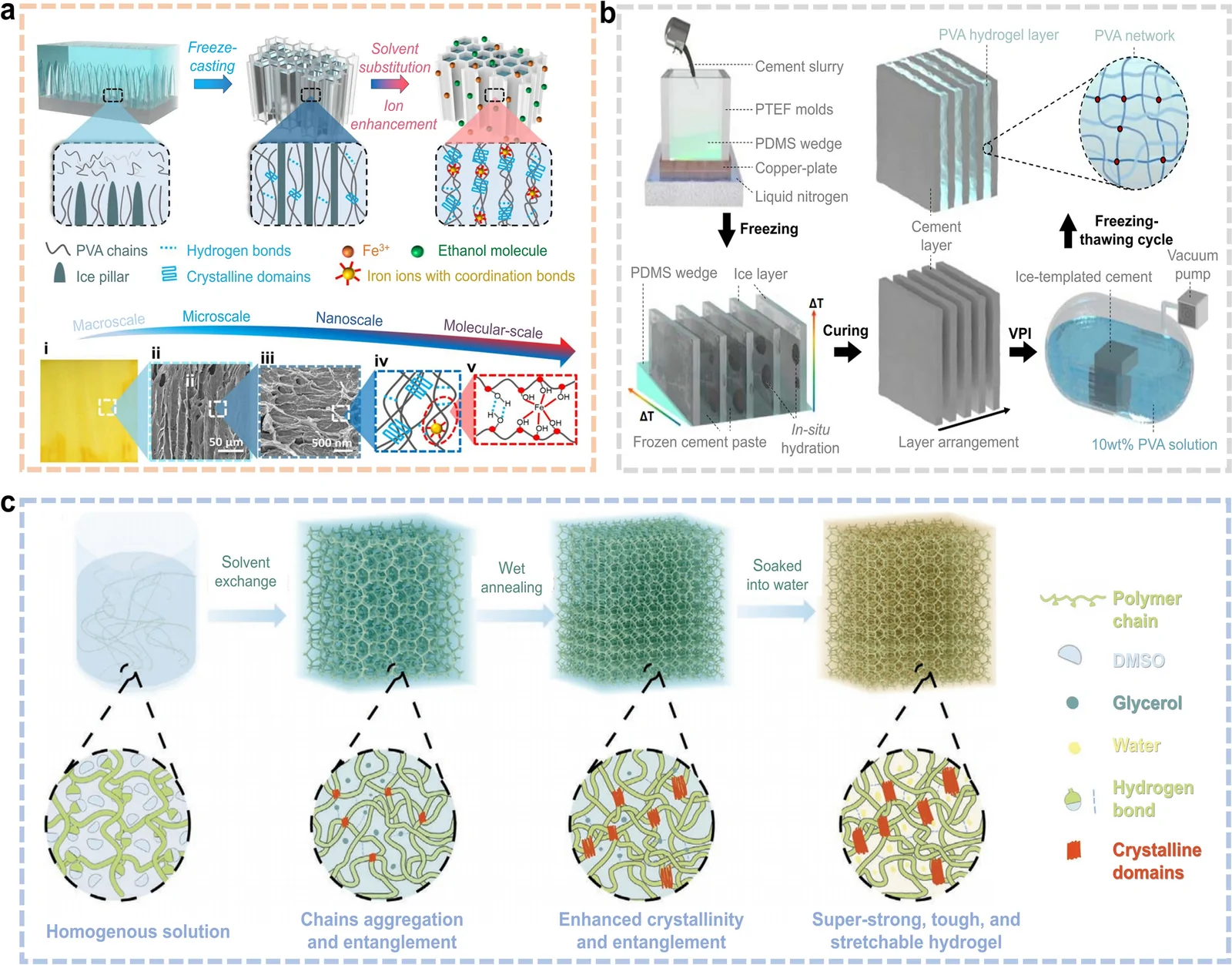
Temperature-induced phase separation strategies for enhancing hydrogel mechanical properties. **a** Freeze-casting-assisted solution substitution produces hydrogels with a multiscale hierarchical structure, including anisotropic fiber honeycombs and nano-grid reinforcements, resulting in superior toughness and tensile strength. **b** Cyclic freeze-thawing using ice crystal templating fabricates lamellar cement–hydrogel composites with improved microcrack deflection and significantly enhanced toughness and flexural strength. **c** Wet annealing via solution exchange increases macromolecular chain mobility, promoting densification, crystallinity, and high-performance mechanical behavior in PVA-based hydrogels. Reproduced with permission from [174–176]

Freeze-casting is a temperature-driven technique that exploits the directional solidification of hydrogel precursor solutions at subzero temperatures to fabricate anisotropic, porous structures. During the freezing process, ice crystals act as dynamic templates, guiding the alignment of polymer chains and creating ordered microchannels. Upon sublimation of the ice, the hydrogel retains a directionally aligned porous architecture, which greatly enhances its mechanical performance, particularly in the direction parallel to the ice growth front. For example, Guo et al. [174] developed a freeze-casting-assisted solution substitution strategy to fabricate hydrogels with a multiscale hierarchical network (Fig. 11a). The resulting structure consisted of anisotropic fiber-honeycomb frameworks, nano-gridded fiber bundles, and PVA molecular chains reinforced by both hydrogen-bonded crystallites and metal coordination bonds. These interconnected structural elements synergistically contributed to exceptional mechanical properties, including a tensile strain of 1623%, tensile strength of 7.11 MPa, and fracture toughness of 58.9 MJ m^−3^. Notably, the elastic modulus of the aligned fiber bundles was 12.3 times higher than that of the surrounding matrix. In parallel, the cyclic freeze–thaw technique is widely used to reinforce hydrogel networks. During the process, ice crystal formation compresses the polymer network, promoting additional physical crosslinking points and resulting in a tighter, more robust structure. This increases hydrogen bonding density and chain entanglement, ultimately enhancing structural integrity and mechanical resilience. Chen et al. [175] developed hydrogel-reinforced cement scaffolds with unidirectional lamellar pores using ice-templating combined with two freeze–thaw cycles (Fig. 11b). This processing induced nanoscale stretching and deflection of microcracks, enhancing toughness by 175-fold and flexural strength by more than twofold.

Wet annealing is a powerful post-treatment strategy by precisely controlling annealing temperature, solvent environment, and chain mobility, and wet annealing rearranges and densifies the internal polymer network, enhancing crosslinking density, crystallinity, and mechanical anisotropy. The process involves heating the hydrogel in a liquid medium, often after solvent exchange, to promote chain relaxation and reorganization. This facilitates chain alignment, increased entanglement, and sometimes crystalline domain formation, substantially improving tensile strength, toughness, and fatigue threshold. In a notable example, Wu et al. [176] developed a dual-step strategy combining wet annealing with solvent substitution to engineer ultra-tough PVA-based hydrogels (Fig. 11c). The resulting hydrogels exhibited remarkable mechanical performance, including a tensile strength of 11.19 ± 0.27 MPa, fracture toughness of 82.28 ± 2.89 MJ m^−3^, and elongation at break exceeding 1879%. Additionally, the hydrogel showed a high fatigue threshold (~ 1233 J m^−2^), confirming its robustness under cyclic mechanical loading. Similarly, Wei et al. [177] combined wet annealing and solution annealing to optimize cellulose-based hydrogels, significantly enhancing polymer chain aggregation, hydrogen bonding, cellulose crystallization, and network densification during low-temperature annealing. This process yielded substantial strength improvements, achieving compressive strength of 21 ± 3 MPa and tensile strength of 7.2 ± 0.7 MPa.

### 3D Printing Strategies for Mechanically Extreme Hydrogels

With the rapid evolution of additive manufacturing technologies, 3D printing has emerged as a transformative tool for fabricating mechanically robust and architecturally complex hydrogels. 3D printing enables bioinspired replication of extracellular matrix architectures via programmable patterning, enhancing hydrogel mechanical compliance for biomedical and robotic applications. It surpasses traditional methods with three key merits: precise material deposition for gradient heterogeneity, architectural freedom for stress optimization, and rheological tunability for reinforcement integration. Synergistic coupling of material composition with geometric design drives breakthrough mechanical enhancements. In the following sections, we explore major 3D printing strategies applied to tough hydrogel fabrication including photo-curing, extrusion-based, inkjet, and laser-assisted printing and discuss how printing parameters, ink formulations, and architectural designs contribute to the realization of mechanically resilient and functional hydrogels.

**Fig. 12:**
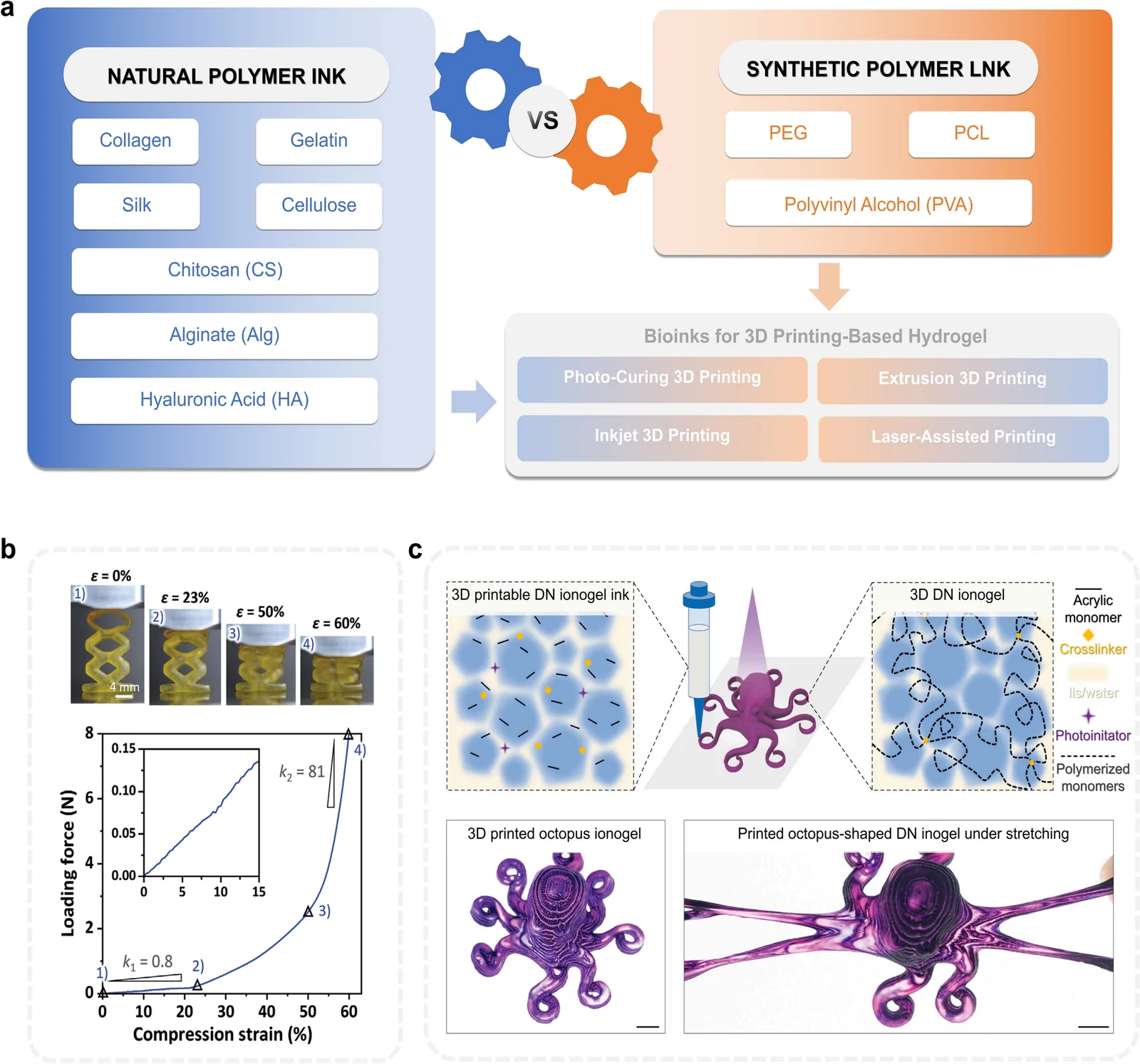
3D printing strategies for the fabrication of strong and tough hydrogels.** a** Common bioink components used in hydrogel 3D printing, including natural and synthetic polymers as well as reinforcing nanomaterials.** b** DLP-printed metal–organic supramolecular hydrogels capable of absorbing high-impact forces and exhibiting excellent compressive resilience under large strains. **c** A 3D-printed octopus-shaped double-network (DN) ion gel, stained with purple dye, demonstrating high stretchability, toughness, and shape fidelity. Reproduced with permission from [191, 192]

In the formulation design of hydrogel-based bioinks for 3D printing, a wide range of natural and synthetic polymers serve as the foundational matrix materials (Fig. 12a). Natural polymers, such as collagen [178], gelatin [179], silk fibroin [180], hyaluronic acid (HA) [181], alginate (Alg) [182], chitosan (CS) [183], and cellulose [184], are frequently selected for their biocompatibility, biodegradability, and physiological relevance. These materials exhibit excellent cytocompatibility and are particularly effective in replicating the structure–function relationships of native extracellular matrices, making them ideal for tissue engineering, organ printing, and bioactive wound healing applications. In contrast, synthetic polymers such as polyethylene glycol (PEG) [185], polyvinyl alcohol (PVA) [186], and polycaprolactone (PCL) [187] offer greater design flexibility, enabling the fine-tuning of mechanical strength, swelling behavior, and degradation rates. Their molecular structure can be precisely modified to meet application-specific requirements, especially in scenarios that demand enhanced mechanical resilience, controlled release profiles, or load-bearing capabilities.

3D printing optimizes hydrogel mechanics and functionality by precise control of critical structural parameters—porosity, fiber alignment, and network architecture—which govern stress distribution, energy dissipation, and functional integration under mechanical robustness and environmental adaptability demands. These include honeycomb structures [188] (designed for isotropic load-bearing), interconnected network structures [189] (that facilitate uniform stress propagation), fiber-based architectures [190] (that mimic collagen bundles or muscle fascicles), and highly porous scaffolds [189] (that enhance compressibility and mass transport). The ability to fabricate such structures with micrometer-scale resolution allows for tailored anisotropy, tunable modulus gradients, and programmable deformation behavior, making them indispensable in applications ranging from soft robotics and flexible electronics to bio-integrated implants and artificial organs.

In the following subsections, we explore four major 3D printing modalities particularly well suited for constructing strong and tough hydrogel systems: (1) photo-curing 3D printing based on UV or visible light–induced polymerization; (2) extrusion-based printing, which offers material versatility and filament continuity; (3) inkjet-based printing, enabling high-resolution patterning and multi-material integration; (4) laser-assisted printing, leveraging energy deposition for rapid structuring and localized reinforcement. These techniques will be discussed in detail with a focus on their working principles, suitability for hydrogel formulations, and their role in enabling mechanically extreme performance through structural and material design.

#### Photo-Curing 3D Printing

Photo-curing 3D printing is a light-assisted additive manufacturing technique that utilizes photopolymerizable hydrogel precursors, enabling layer-by-layer solidification through spatially controlled light exposure. This process provides exceptional resolution and architectural fidelity, making it particularly suitable for fabricating complex, mechanically reinforced hydrogel structures. The primary photo-curing modalities include stereolithography (SLA) [193], digital light processing (DLP) [194], and two-photon polymerization (TPP) [195]. These technologies rely on light-sensitive photoinitiators embedded within the hydrogel matrix, which initiate chain-growth polymerization or crosslinking reactions upon exposure to specific wavelengths [196]. Among these, DLP-based hydrogel printing has gained increasing attention for its speed, scalability, and ability to achieve microscale resolution. For instance, Wang et al. [196] developed a DLP printing platform integrated with a microfluidic mixer, enabling real-time modulation of bioink composition. This approach allowed the fabrication of tough hydrogels with spatially graded properties, including cell density, chemical composition, mechanical modulus, and porosity, closely mimicking the hierarchical heterogeneity of native tissues. The printing system projected patterned light via a digital micromirror device (DMD), initiating layer-by-layer photopolymerization to form intricate, gradient-structured hydrogels ideal for applications in tissue engineering and regenerative medicine.

To address limitations in resolution and throughput, Wu et al. [197] introduced a soft, deformable separation interface to accelerate the printing process while maintaining high aspect ratios. This enabled rapid DLP printing of high-fidelity cylindrical structures (500 µm in diameter) at a speed of 400 mm h^−1^, showcasing the compatibility of photo-curing strategies with complex, large-volume hydrogel constructs. Moreover, Dong et al. [191] demonstrated the potential of DLP printing for tough supramolecular hydrogels based on carboxyl–Zr^4+^ coordination complexes. The printed structures exhibited low initial stiffness and a capacity to absorb impact through sequential deformation of structural units (Fig. 12b). This design enabled the development of digitally patterned soft impact buffers, capable of dissipating mechanical energy efficiently under compression, highlighting the synergy between structural programmability and supramolecular chemistry in enhancing mechanical performance. In summary, photo-curing 3D printing, particularly DLP technology, enables the precise fabrication of hydrogels with spatially programmed composition, mechanical gradients, and architectural complexity. These features are crucial for realizing hydrogels that meet the mechanical demands of real-world applications, from load-bearing implants to impact-tolerant soft devices.

#### Extrusion 3D Printing

Extrusion-based 3D printing, also known as pressure-assisted or direct ink writing (DIW), is a widely adopted method for constructing mechanically robust, cell-compatible hydrogel architectures. In this technique, viscous hydrogel inks typically composed of polymer solutions or particle-laden suspensions are extruded through syringes or precision nozzles driven by pneumatic, piston, or screw-based systems. As the nozzle traverses the X–Y–Z coordinates based on a CAD design, the hydrogel is deposited layer by layer to form three-dimensional structures with tunable architecture and mechanical performance [198]. A major advantage of extrusion printing lies in its compatibility with a wide range of viscoelastic bioinks, including those with embedded nanofillers, reinforcing networks, or living cells. This technique also allows for multi-material co-printing, enabling the integration of mechanical gradients, conductive pathways, or biological interfaces in a single construct.

Zheng et al. [199] demonstrated that incorporating β-chitosan nanofibers significantly enhanced the rheological and mechanical properties of a chitosan-based hydrogel ink. By tuning the nanofiber content optimally to 5–10 wt%, they achieved high print fidelity, self-supporting capacity, and structural stability during direct ink writing. This strategy effectively combined biodegradability with mechanical toughness, making the printed scaffolds suitable for biomedical implants. Furthermore, Yao et al. [192] developed a double-network (DN) ion gel ink with exceptional mechanical properties. Amphoteric ion hydrogels were synthesized, freeze-dried, ground into microparticles, and re-swollen in ionic liquids to form a printable matrix. Subsequent monomer infusion, UV crosslinking, and DIW printing enabled the formation of octopus-shaped DN gels with > 600% stretchability and fracture energy exceeding 10 kJ m^−2^ (Fig. 12c). These architectures demonstrated programmable deformation, resilience, and ionic conductivity.

Extrusion-based 3D printing offers a versatile and scalable route to fabricating tough, functional, and structurally complex hydrogels. Through precise control over ink formulation, deposition path, and post-treatment, this technique enables the creation of biomimetic structures with enhanced Young’s modulus, fracture toughness, energy dissipation, and bio-integration capacity, pushing the boundaries of hydrogel performance under mechanical and functional demands.

#### Inkjet 3D Printing

Inkjet 3D printing is a non-contact additive manufacturing technique that evolved from traditional 2D industrial printing. As one of the earliest methods adapted for bioprinting, inkjet printing offers high-resolution deposition, rapid material switching, and minimal material waste, making it particularly advantageous for applications requiring precise patterning, multi-material integration, and bioactive encapsulation. Inkjet printing systems are primarily categorized into two modes of continuous inkjet (CIJ) and drop on demand (DOD) [200]. In CIJ printing, a continuous pressurized liquid stream is ejected through a micro-nozzle, and due to Rayleigh–Plateau instability, the jet breaks into droplets [200]. These droplets are selectively charged via an electrode and deflected onto a substrate using an electric field. Non-deposited droplets are recirculated, ensuring material efficiency. However, due to its complexity and droplet control limitations, CIJ is less commonly used in hydrogel bioprinting. DOD printing, on the other hand, ejects droplets only when triggered by a pulse signal, usually from a piezoelectric actuator. This enables precise spatial control, reduced shear stress, and compatibility with biological materials. DOD systems often incorporate multiple printheads, allowing for the co-deposition of different materials or cells within complex hydrogel matrices. For example, Peng et al. [201] utilized drop-on-demand inkjet printing to deposit bioinks containing cells and functional biomaterials onto predefined substrates. Subsequent photopolymerization or thermal curing produced 3D hydrogel constructs with high mechanical integrity and biocompatibility. This process enabled the fabrication of tough, patterned constructs suitable for tissue engineering scaffolds and biomedical device coatings. Kim et al. [202] demonstrated the use of a piezoelectric inkjet printer to precisely deposit polyethylene glycol diacrylate (PEGDA) hydrogel precursors onto a micropillar-patterned PDMS elastomer substrate. Following UV-induced photopolymerization, the resulting hydrogel-elastomer composite exhibited strong interfacial adhesion, excellent mechanical robustness, and anti-fogging capabilities. The printed micropattern retained optical transparency and mechanical stability under high humidity and thermal stress, illustrating the potential of inkjet-printed hydrogels for advanced surface engineering and optical applications.

In summary, inkjet-based 3D printing offers a precise, versatile, and contactless strategy for fabricating microscale hydrogel architectures. Though somewhat limited by the rheological constraints of printable inks, this technique excels in scenarios demanding high-resolution patterning, localized property modulation, and biologically compatible constructs.

#### Laser-Assisted Printing

Laser-assisted printing (LAP) is a high-precision, non-contact fabrication technique originally developed for metal deposition via laser-induced forward transfer (LIFT). Since its successful adaptation for biological systems in the year of 2004 including the transfer of cells, DNA, and bioinks [203], LAP has emerged as a powerful tool for micro- and nanoscale patterning of functional hydrogels. The standard LAP setup comprises three core components of (1) a laser source, (2) a ribbon (donor substrate) coated with the desired bioink atop a thin metal absorbing layer, and (3) a receiving substrate onto which the material is deposited. When the laser irradiates the metal layer, it generates localized heating and pressure, propelling a droplet of the bioink forward onto the target substrate without direct contact. Beyond droplet transfer, LAP is increasingly being used for direct laser structuring and microscale patterning within soft hydrogel matrices. For example, Fan et al. [204] utilized femtosecond lasers to induce volume phase transitions in thermoresponsive hydrogels such as PNIPAAm and PEGDA. Point-by-point scanning led to nanoscale wrinkle formation via localized heating and subsequent cooling-induced stress mismatch. This technique enabled the generation of hierarchical wrinkle structures in a single step and single material system, providing a path toward hydrogels with tunable surface morphology, enhanced surface area, and improved mechanical behavior. In another study, Li et al. [205] applied LAP for layer-by-layer 3D structuring of hydrogels. By finely adjusting the laser power and scan rate, they induced localized gelation within liquid hydrogel precursors, enabling the construction of complex three-dimensional networks. This method preserved the inherent biocompatibility and porosity of the hydrogel while offering control over internal architecture, which translated into notable improvements in tensile strength, compressive modulus, and fracture toughness. The resulting hydrogels showed excellent shape fidelity and were particularly well suited for soft electronics, microscale actuators, and cell-laden constructs requiring extreme mechanical resilience. Laser-assisted printing offers unique advantages for fabricating mechanically enhanced hydrogels with micro- and nanoscale spatial resolution. Its ability to pattern internal microstructures, induce dynamic surface features, and trigger localized crosslinking without physical contact makes it an ideal platform for next-generation smart hydrogel systems.

### Stimulus-Responsive Modulation of Mechanical Properties

Inspired by adaptive behaviors observed in biological systems, stimuli-responsive materials have emerged as a compelling class of bioinspired platforms capable of dynamically adjusting their structure and function in response to environmental changes. Notably, when tailored for mechanical extremity, such hydrogels offer unique advantages for applications requiring adaptive stiffness, real-time actuation, and damage mitigation under variable operating conditions.

A fundamental feature of these smart hydrogels is their ability to transduce external physical or chemical stimuli into mechanical responses. These hydrogels are exposed to changes in temperature, pH, ion concentration, electric or magnetic fields, or even biochemical signals. Such exposure triggers internal reconfigurations, including the reorganization of polymer chains, shifts in water distribution, or alterations in dynamic crosslinks, which ultimately modulate the hydrogel’s modulus, strength, and toughness. This microstructural reconfiguration allows external cues to directly influence macroscale mechanical behaviors. Consequently, it enables new functionalities such as tunable elasticity, programmable damping, and strain-adaptive energy dissipation. During these stimuli-induced transitions between hard and soft states, additional functional attributes such as volume change, optical clarity, hydrophilicity, and ionic/electronic conductivity are often concurrently modulated. Although extensive studies have focused on the swelling behavior, optical response, and conductivity modulation of stimuli-responsive hydrogels, research into the precise regulation of their mechanical properties by external stimuli remains comparatively underdeveloped. Particularly, the mechanistic understanding of how external fields manipulate internal network topology, dynamic bond kinetics, or phase behavior to achieve predictable changes in tensile strength, compressive modulus, or fracture energy is still in its early stages.

In this section, we aim to systematically explore how external stimuli modulate the mechanical properties of tough hydrogels, focusing on four representative categories of thermal responsiveness, pH responsiveness, electric field responsiveness, and magnetic field responsiveness. By examining the underlying mechanochemical coupling mechanisms and the resulting macro-mechanical performance shifts, this discussion seeks to provide both theoretical insight and engineering guidance for the design of next-generation adaptive hydrogels tailored for extreme mechanical demands in dynamic environments.

#### Temperature-Responsive

Temperature-responsive hydrogels exhibit a pronounced ability to modulate their structure and properties in response to thermal stimuli. These materials undergo a reversible sol–gel phase transition at a specific critical solution temperature, making them highly attractive for applications in soft actuators, drug delivery, and adaptive biomedical scaffolds. Thermoresponsive hydrogels are typically classified into two main categories based on their response direction: (1) lower critical solution temperature (LCST)-type hydrogels (negatively thermoresponsive), which collapse or shrink when the ambient temperature exceeds the LCST, and swell when the temperature drops below it; (2) upper critical solution temperature (UCST)-type hydrogels (positively thermoresponsive), which in contrast swell upon heating above the UCST and shrink when cooled below this threshold. The LCST and UCST thus define the characteristic temperatures at which reversible phase transitions occur, dictating the direction of thermoresponsive swelling or collapse. The thermoresponsiveness of such systems is governed primarily by the balance between hydrophilic and hydrophobic interactions and is strongly influenced by the formation and disruption of hydrogen bonds. As temperature increases, hydrogen bonding may be weakened, promoting hydrophobic interactions and triggering polymer chain aggregation or collapse, which is the central mechanism behind the volume phase transition. These thermally tunable characteristics not only allow precise control over mechanical stiffness, toughness, and resilience, but also support integration into environments with cyclical thermal loads or on-demand actuation requirements. Importantly, temperature-responsive tough hydrogels offer pathways for engineering reversible hard–soft transitions, localized mechanical reinforcement, and stimuli-adaptive damping behaviors, making them promising candidates for wearable devices, tissue mimics, and robotic components operating under varying thermal conditions.

**Fig. 13:**
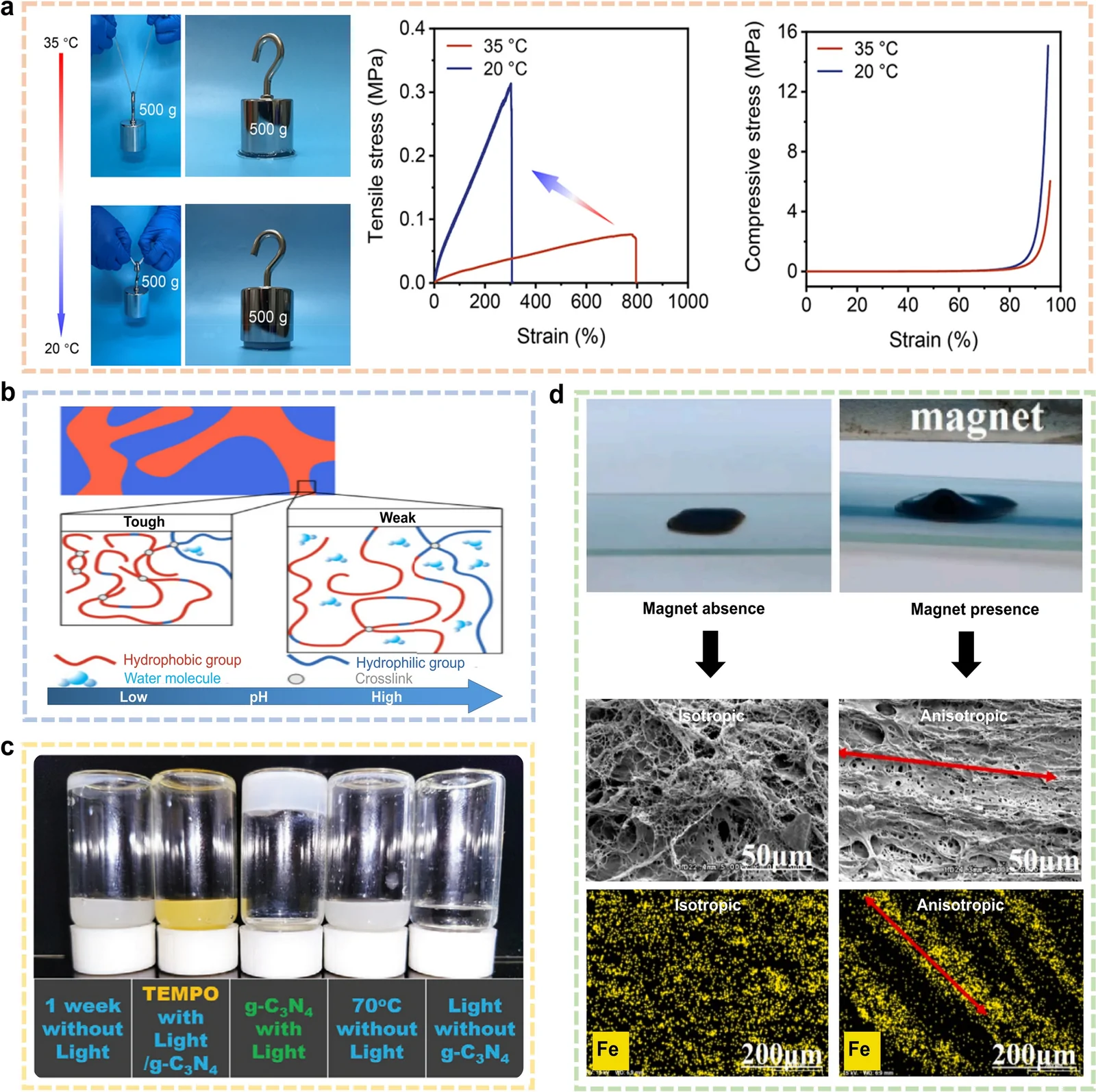
Various stimuli regulating the mechanical properties of hydrogels. **a** Temperature-induced stress–strain behavior modulation in thermoresponsive hydrogels, highlighting the effects of hydrogen bonding and phase separation mechanisms. **b** pH-sensitive hydrogels exhibiting substantial modulus variation in response to minimal pH shifts, driven by ionization and osmotic pressure effects. **c** Visible light-triggered polymerization using a graphene-based photocatalyst, enabling the fabrication of mechanically robust and low-hysteresis hydrogels. **d** Magnetic field-guided alignment of nanoparticles within the hydrogel matrix, forming an anisotropic architecture that significantly enhances tensile strength, compressive resilience, and tribological performance. Reproduced with permission from [19, 210, 218, 222]

One of the key mechanisms by which temperature-responsive hydrogels achieve enhanced mechanical performance is through polymer phase separation, which facilitates the self-assembly of polymer chains into physically reinforced frameworks. This microstructural reorganization increases the network’s stiffness, toughness, and resistance to deformation, providing a robust strategy for mechanically strengthening smart hydrogels under thermal stimuli. Yang et al. [206] developed a fast-response bilayer hydrogel actuator by combining hydrogels with distinct LCSTs. The device exhibited thermally tunable bending behavior, where the deformation rate was governed by both temperature elevation and thickness ratio between the layers. Upon heating to the LCST, phase separation induced localized contraction, producing rapid shape transformation due to differential strain between the bilayers. At the molecular level, PNIPAAm chains self-associated into a polymer-rich phase, forming a dense. In a related study, Zhang et al. [19] leveraged temperature to reversibly modulate hydrogen bonding interactions between poly(acrylic acid-acrylamide) copolymers and cellulose nanofibrils. By thermally driving the association and dissociation of hydrogen bonds, they enabled on-demand control over interfacial toughness and mechanical stiffness, with the compressive modulus tunable from 0.14 to 0.02 MPa (Fig. 13a).

Another critical mechanism underlying the thermal adaptability of tough hydrogels is the glass transition, a phenomenon wherein the polymer matrix undergoes a reversible shift from a rigid, glassy state to a soft, rubbery state as the temperature crosses a defined glass transition temperature (*T*_g_). Hou et al. [207] designed a poly(AAm-co-MAAc) supramolecular hydrogel that exhibits a glassy nature at room temperature, driven by dense intermolecular hydrogen bonding networks. Interestingly, upon cooling to – 45 °C, the hydrogel becomes stronger and stiffer with a Young’s modulus of 900 MPa, tensile strength of 30 MPa, and elongation at break of 35%, yet retains sufficient ductility. This counterintuitive enhancement under subzero conditions is enabled by the strengthening of hydrogen bonds during cooling, coupled with a glass transition-induced energy dissipation mechanism. The result is a material capable of operating under extreme cold environments without loss of mechanical reliability or optical clarity. The exploitation of glass transition dynamics provides an effective strategy to design temperature-adaptive hydrogels capable of maintaining extreme mechanical performance across diverse environmental conditions, particularly in cold climates or applications demanding impact resistance and dimensional stability.

The incorporation of fusible linkages has emerged as a compelling strategy to enhance both the thermal responsiveness and mechanical extremity of hydrogels. Inspired by the thermal behavior of crystalline materials that soften upon heating above their melting point, this design principle leverages thermodynamic phase transitions to achieve reversible modulation of mechanical properties. By engineering semicrystalline polymer networks or introducing crystallization-promoting additives, hydrogels can dynamically transition between rigid and soft states, enabling functionalities such as shape memory, self-healing, and thermal actuation. Okay et al. [208] developed a class of thermoresponsive hydrogels by copolymerizing acrylic acid with 20–50 mol% of crystalline acrylic acid octadecyl ester. Within these networks, hydrophilic segments conferred flexibility, while hydrophobic crystalline domains served as thermally fusible crosslinks, providing rigidity and structural reinforcement. Upon heating, these crystalline domains undergo melting, temporarily softening the material. Upon cooling, recrystallization restores the original stiffness, yielding a material with remarkable compressive strength (up to 90 MPa) and an extraordinarily high Young’s modulus (26 GPa). In addition, these hydrogels exhibited shape memory behavior and self-healing capabilities, facilitated by the reversible nature of the crystalline crosslinks. In a similar approach, Zhao et al. [209] designed hydrogels embedded with semicrystalline comb-like polymer networks derived from stearyl methacrylate (SMA). These SMA-based networks incorporate microphase-separated crystalline side chains, which undergo melting upon heating, transitioning the material into a soft and elastic state. This results in a sharp drop in elastic modulus from 7.2 MPa (at 20 °C) to 0.47 MPa (at 60 °C). Conversely, upon cooling, the reformation of crystalline domains reinstates the hydrogel’s rigidity and load-bearing capacity, allowing it to recover its mechanical strength without structural degradation.

Studying the temperature-responsive hydrogels holds both theoretical and applied significance. Theoretically, it elucidates how thermally driven processes including polymer phase separation and glass transitions govern the network dynamics and energy dissipation of hydrogels. Practically, this understanding enables adaptive hydrogels that meet dynamic mechanical demands in tissue engineering, achieve temperature-triggered drug release, and serve as smart materials in wearables and soft robotics capable of real-time response to thermal changes.

#### pH-Responsive

The pH-responsive hydrogels enable on-demand mechanical tuning through real-time performance adaptation, making them promising for targeted delivery, smart diagnostics, and environmentally responsive devices. The pH-responsive hydrogels are polymeric networks that undergo conformational and volumetric changes in response to variations in environmental pH, due to the presence of ionizable functional groups along the polymer backbone. These materials exhibit reversible swelling and contraction behavior driven by the gain or loss of protons, making them particularly attractive for applications requiring stimulus-dependent mechanical modulation. In anionic hydrogels which incorporate acidic moieties such as carboxylic or sulfonic acid groups, an increase in pH above the polymer’s acid dissociation constant (pKa) leads to deprotonation, generating negatively charged sites. This increases electrostatic repulsion, resulting in network expansion and macroscopic swelling. In contrast, cationic hydrogels containing basic groups (e.g., amines) become protonated under acidic conditions, similarly triggering swelling due to Coulombic repulsion among positively charged chains. These pH-mediated changes in ionic density and osmotic pressure significantly influence the mechanical stiffness, toughness, and resilience of the hydrogel. Three key mechanisms underlie the mechanical modulation of pH-responsive hydrogels, including: (1) ionization-induced swelling enhances chain separation and network porosity, modulating stress transfer pathways [210], (2) charge shielding, especially in buffered environments, reduces electrostatic interactions and enables tunable softening or stiffening responses [211], and (3) hydrogen bonding disruption or formation at specific pH values contributes to dynamic crosslinking behavior, providing reversible energy dissipation and recovery under load [212].

In the study by Yang et al. [213], the mechanical properties of the hydrogel were precisely modulated through pH-responsive network dynamics. The system employed Pluronic F-127 (F-127) micelles and poly(2-hydroxyethyl methacrylate) (PHEMA) chains to form a phase-separated cluster architecture stabilized by hydrogen bonding and hydrophobic interactions. These clusters imparted the hydrogel with high toughness and anti-swelling behavior. Upon the introduction of hydrochloric acid (HCl), protonation of hydroxyl and carbonyl groups on the PHEMA chains disrupted the interfacial hydrogen bonds with F-127, thereby reducing the interaction strength. This transition led to swelling-induced softening and optical transparency changes, showcasing the reversible tuning of hydrogel mechanics via pH control. When neutralized with NaOH, the original mechanical state was restored, highlighting a robust and fully reversible pH-driven mechanical switch. Similarly, Wang et al. [210] demonstrated ultrasensitive pH-dependent stiffness modulation, although the underlying mechanism differed. Their hydrogel system exhibited a 50-fold change in modulus in response to a minute pH variation of just 0.06 units (Fig. 13b).

#### Light-Responsive

Light serves as a compelling external stimulus in hydrogel design due to its non-contact nature, high spatial–temporal resolution, and minimal biological interference. These characteristics make it an ideal tool for modulating the mechanical behavior of hydrogels with precision. The mechanical enhancement or tuning mechanisms in light-responsive hydrogels fall into three main domains of (1) double bond isomerization [214] (altering chain rigidity or conformation to switch between soft and stiff states); (2) photosensitive group reactions [215] (triggering crosslinking or degradation based on photochemical cleavage or bonding); and (3) additive reinforcement [216] (where nanofillers or photoactive compounds modulate mechanical strength, enabling dynamic shape memory or adaptive damping).

The mechanical performance of light-responsive hydrogels is primarily governed by light-induced crosslinking reactions, molecular conformational changes, and photothermal effects. Variations in light wavelength, intensity, and duration can trigger distinct physical and chemical transitions within the polymer network, significantly modulating its modulus, toughness, elasticity, and recovery behavior. Zheng et al. [217] demonstrated a multistimuli-responsive hydrogel system incorporating spirobenzopyran as a hydrophobic crosslinker into a hydrophilic polymer matrix via UV-induced polymerization and micellar copolymerization. Upon UV exposure, the spirobenzopyran undergoes cleavage and ring-opening reactions, which reinforce the network structure. These transitions, initiated not only by light but also by thermal and mechanical stimuli, provide a multistage, mechanically adaptive hydrogel. et al. [218] utilized a graphitic carbon nitride (g-C_3_N_4_) photocatalyst to initiate acrylamide polymerization under visible light, forming hydrogels with low hysteresis and high elasticity (Fig. 13c). The g-C_3_N_4_ not only facilitated efficient photopolymerization but also enhanced polymer chain entanglement, increasing the hydrogel’s tensile strength by over 1000%. To circumvent the penetration limitations of UV and visible light in biomedical settings, Ryan et al. [219] introduced a near-infrared (NIR) triggered crosslinking system using gold nanorods. These nanostructures convert NIR light into localized heat, facilitating the release of calcium ions or chelating agents that dynamically elevate crosslinking density. This process not only boosts the Young’s modulus of the hydrogel but also mimics dynamic extracellular matrix (ECM) stiffening, which plays a critical role in cell proliferation, migration, and differentiation.

Light-responsive hydrogels exhibit exceptional potential for programmable modulation of mechanical properties, offering a noninvasive, spatiotemporally precise, and tunable approach to material adaptability. By leveraging diverse light sources including ultraviolet (UV), visible (Vis), and near-infrared (NIR) light, these systems enable real-time adjustment of stiffness, toughness, and elasticity through a variety of mechanisms, such as photo-initiated crosslinking, molecular isomerization, and localized photothermal effects. Importantly, light-responsive hydrogels enable on-demand mechanical actuation, anisotropic stiffness gradients, or localized reinforcement, offering unique capabilities for precision mechanical control in real time. These features are particularly advantageous for mechanically extreme applications, where adaptability under cyclic or variable stress is essential.

#### Magnetic-Responsive

Magnetic-responsive hydrogels are a class of smart materials that integrate the soft, hydrated nature of traditional hydrogels with magnetically tunable mechanical behavior [220]. These materials typically consist of a polymeric network embedded with magnetic nanoparticles (MNPs) such as Fe_3_O_4_. When exposed to an external magnetic field, the magnetic particles undergo orientation, alignment, or aggregation, inducing localized changes in the hydrogel’s microstructure [221]. For instance, Chen et al. [222] developed a strategy to precisely modulate the mechanical properties of hydrogels through magnetic field-assisted structural induction. In their design, polydopamine (PDA) was employed as a chelating interface to facilitate the in situ growth of Fe_3_O_4_ nanoparticles on montmorillonite (MMT) surfaces, utilizing mussel-inspired adhesion chemistry. These PDA- Fe_3_O_4_-MMT nanohybrids were uniformly dispersed within a poly(vinyl alcohol)/poly(acrylic acid) (PVA/PAAc) hydrogel matrix. Upon application of an external magnetic field, the embedded nanoparticles self-organized into an anisotropic alignment, which was subsequently fixed via freeze–thaw cycles and thermal annealing (Fig. 13d). This process yielded a hydrogel with a directionally structured network, resulting in significant improvements in mechanical performance: a tensile strength of 10.65 MPa, toughness of 52.2 MJ m^−3^, and compressive strength of 4.86 MPa. In another example, Wang et al. [223] engineered an ultra-tough magnetic hydrogel with uniformly distributed Fe_3_O_4_ nanoparticles embedded within a poly(N,N-dimethylacrylamide) (PDMA) matrix crosslinked by titanium dioxide (TiO_2_) nanoclay. The Fe_3_O_4_ nanoparticles served as dynamic crosslinking agents, engaging in non-covalent interactions with the PDMA chains. During phase separation induced in a NaOH environment, an applied magnetic field further aligned the nanoparticle network, promoting hierarchical reinforcement. This led to a toughness of 11 kJ m^−2^, a marked enhancement compared to non-magnetic counterparts.

The imposed anisotropy via magnetic alignment not only optimizes the internal architecture of the hydrogel but also endows it with adaptive responsiveness to diverse external stimuli, such as mechanical load, temperature, or electromagnetic signals. This programmable responsiveness greatly expands the functional landscape of magnetic hydrogels, particularly for applications in biomedical engineering (e.g., targeted drug delivery, implantable soft robotics), smart adaptive materials, and high-sensitivity sensor platforms. A fundamental yet unresolved question is how competing or synergistic effects from multiple simultaneous stimuli (e.g., temperature and magnetic field) affect the mechanical integrity and fatigue life of these hydrogels, which is critical for applications in complex, real-world environments.

## Applications of Mechanically Extreme Hydrogels

**Fig. 14:**
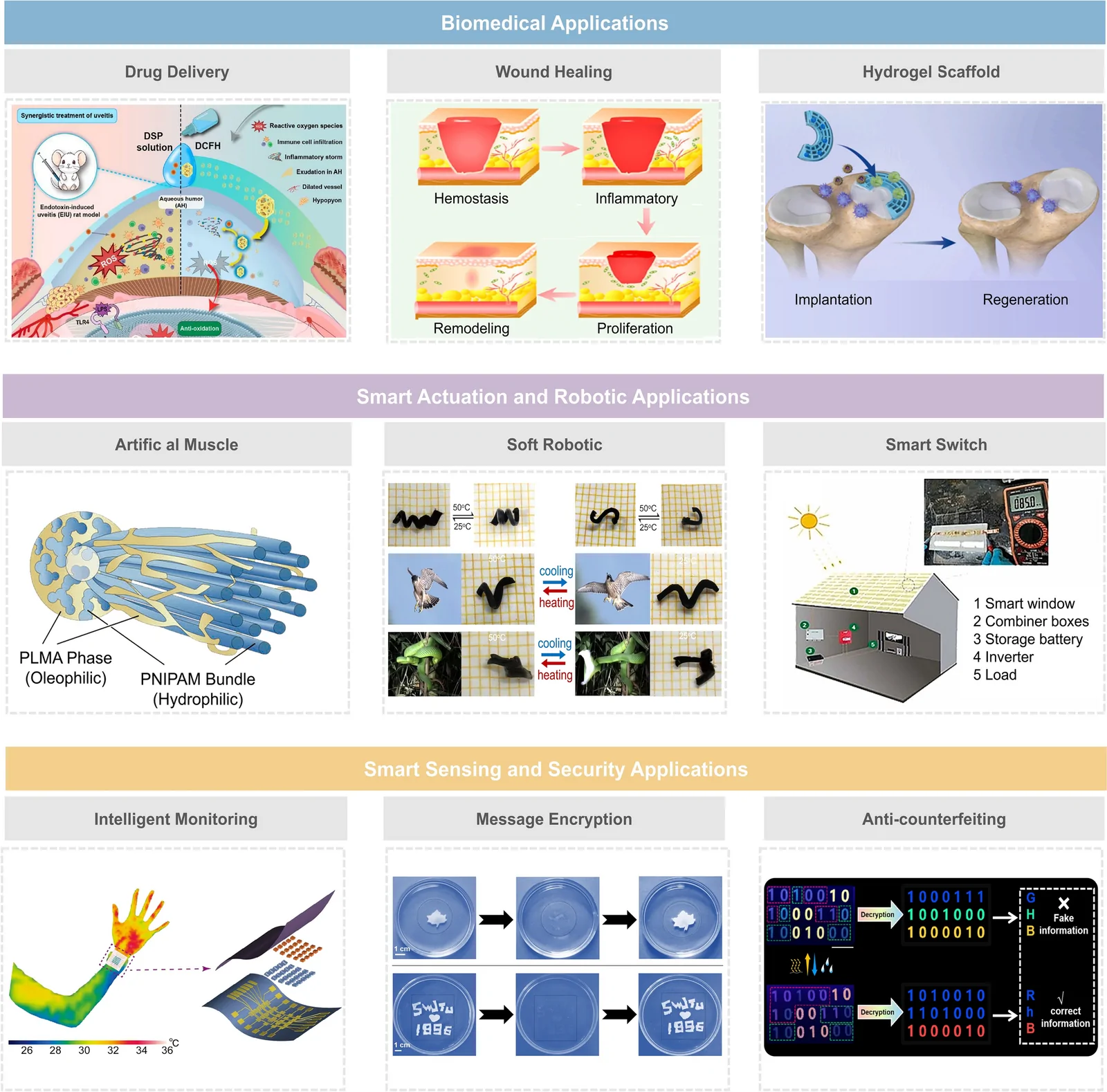
Intelligent applications of extreme-mechanics hydrogels. Reproduced with permission from [3, 7, 224–227]

In recent years, mechanically extreme hydrogels, characterized by their exceptional strength, toughness, and functional adaptability, have attracted considerable attention across a wide range of disciplines. Inspired by structural dynamics in biological systems, these programmable deformations endow hydrogels with significant application potential in responsive and multifunctional environments. As research continues to advance, the integration of mechanical resilience with multimodal responsiveness is expected to transform hydrogels into pivotal materials for emerging technologies in bioelectronics, human–machine interfaces, and adaptive infrastructure. This chapter provides a comprehensive overview of the key application domains for mechanically extreme hydrogels, outlining their practical advantages, current challenges, and future opportunities (Fig. 14).

### Tailoring Mechanical Properties for Targeted Extreme Environments

The pursuit of mechanically extreme hydrogels must be guided by a fundamental principle: Mechanical performance is not an absolute metric but a context-dependent requirement. As highlighted in this review, while significant advances have been made in enhancing strength, toughness, and fatigue resistance (Sects. 2 and 3), the ultimate measure of success lies in how well these properties align with the specific demands of the intended operating environment. Hydrogels designed for deep-sea exploration face drastically different mechanical challenges than those intended for epidermal electronics, neural interfaces, or aerospace applications. This section establishes a scenario-adaptive design framework that prioritizes mechanical properties based on four major categories of extreme environments, providing a roadmap for translating fundamental advances into practical, high-performance systems (Table 5).

**Table 5:** Mechanical requirements of hydrogels for major extreme application scenarios

Application scenario	Core mechanical demand	Key performance indicators	Typical environment and challenges	References
Marine/Underwater	Structural integrity under pressure	High compressive strength (MPa range); Low swelling in saline solutions; High fracture toughness & tear resistance; Fatigue resistance under cyclic loading	Environment: deep-sea exploration, underwater robotics, marine sensing; Challenges: hydrostatic pressure, saline corrosion, biofouling	[256]
Wearable/Epidermal	Compliance & conformal adhesion	Low elastic modulus (kPa-MPa, matching skin); High stretchability with low hysteresis; High resilience & elastic recovery; Strong yet compliant adhesion to dynamic tissues	Environment: health monitoring, wearable electronics, smart textiles; Challenges: dynamic body motion, sweat, long-term skin comfort	[228]
Aerospace/Extreme Temperature	Temperature-resilient mechanics	Low-temperature toughness (anti-freezing); High-temperature stability; Resistance to dehydration/vacuum; Radiation tolerance	Environment: space exploration, polar missions, high-altitude devices; Challenges: cryogenic temperatures, vacuum, thermal cycling, radiation	[229, 230]
Biomedical	Function-specific mechanical matching	Load-bearing implants: High compressive/wear resistance; Neural interfaces: Ultra-low modulus (∼kPa), minimal mismatch; Cardiac/vascular patches: High fatigue threshold; Drug carriers: Stimuli-responsive modulus/swelling changes	Environment: Tissue engineering, implantable devices, drug delivery; Challenges: physiological loading, biodegradation, immune response, dynamic tissue environments	[231, 232]

The diversity of requirements outlined in Table 4 necessitates equally diverse material strategies. It is not merely a matter of achieving higher values in traditional metrics, but rather strategically prioritizing certain properties while accepting necessary trade-offs in others. For instance, a hydrogel optimized for neural interfaces may sacrifice compressive strength to achieve ultralow modulus and minimal mechanical mismatch with brain tissue [232], while a marine actuator might prioritize pressure resistance and low swelling over extreme stretchability [233]. Nam et al. [232] developed a soft neural interface based on a supramolecular β‑peptide hydrogel. This material possesses an elastic modulus of approximately 1500 Pa, which matches the softness of neural tissue and helps minimize mechanical damage during implantation. Furthermore, the hydrogel exhibits an impedance below 0.2 MΩ within the physiologically relevant frequency range (0–200 Hz), demonstrating excellent electrical properties suitable for stable neural signal recording. Ren et al. [233] successfully fabricated a high-strength hydrogel actuator with anti-swelling capability by blending the pH-responsive copolymer [2-(methacryloyloxy) ethyl] dimethyl-(3-sulfopropyl) ammonium hydroxide/2-hydroxyethyl methacrylate (SBMA-HEMA) with PVA. This hydrogel actuator exhibits a high compressive modulus of up to 8.12 MPa, along with a toughness of 518 kJ m^−3^. Its high strength originates from the effective energy dissipation mechanism of the blended network. Meanwhile, the protonation (positively charged) of SBMA units in acidic environments reduces osmotic pressure and enhances electrostatic repulsion, which drives water molecules out, resulting in a low equilibrium swelling ratio of only 9% after 30 days in water.

### Biomedical Applications

#### Drug Delivery

Mechanically extreme hydrogels serve as critical enablers in advanced drug delivery systems, particularly in localized therapy, sustained-release platforms, and stimuli-responsive targeted delivery [9]. Their outstanding mechanical strength and toughness ensure structural integrity under physiological stresses, effectively preventing deformation, rupture, or material degradation during application. This mechanical resilience is pivotal for maintaining carrier stability and enabling temporally controlled drug release across diverse physiological environments. For instance, Giovanni Traversa’s group [234] introduced an innovative oral drug delivery strategy termed LIFT (liquid in situ-forming and tough) hydrogels, wherein liquid formulations transform into robust hydrogels within the gastric environment. This transformation is driven by ionic crosslinking between alginate and functionalized polyethylene glycol (PEG), triggered by gastric acid.

**Fig. 15:**
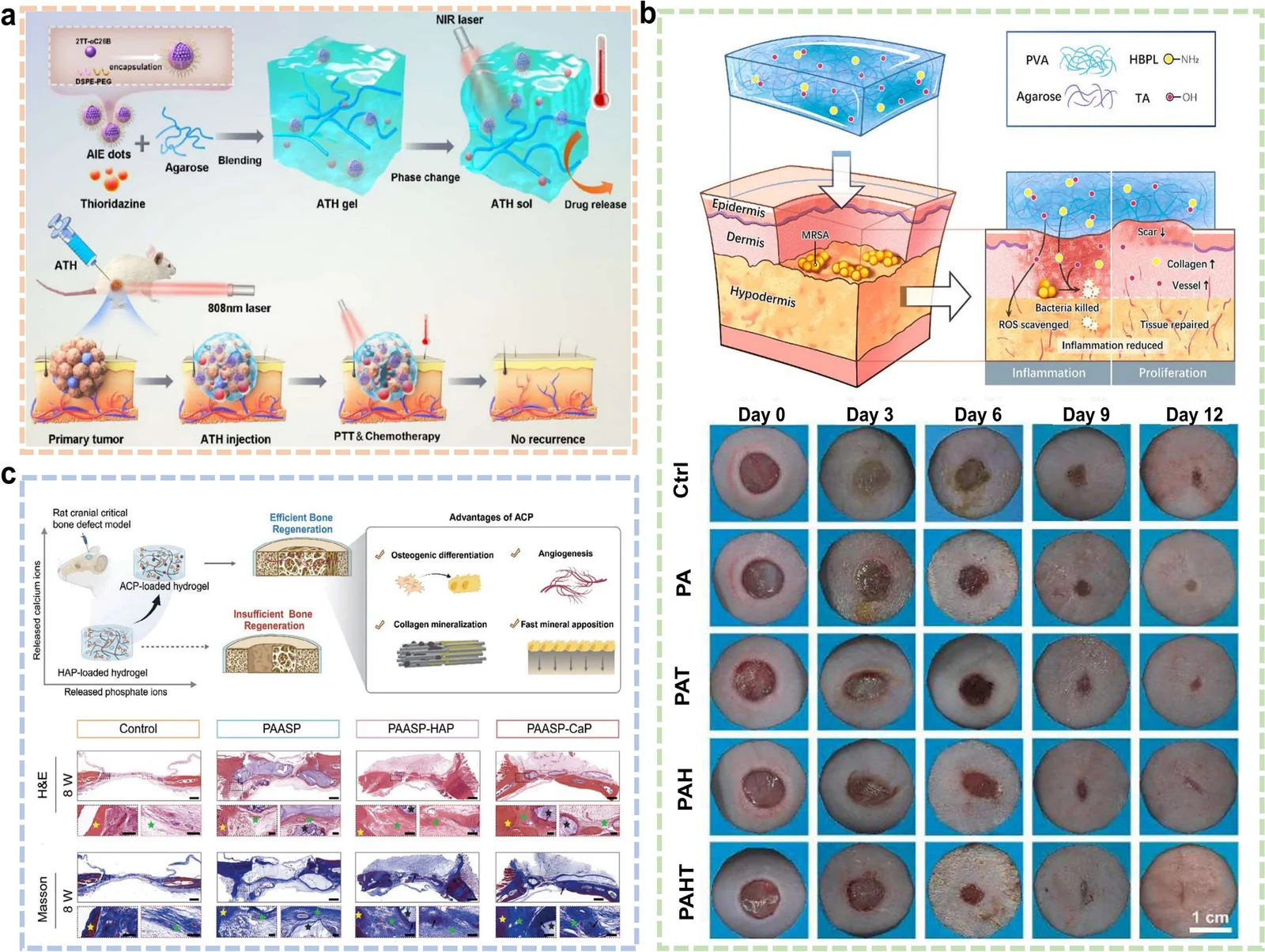
Biomedical applications of mechanically extreme hydrogels. **a** NIR-triggered hydrogel photothermal effects enhance localized drug release for deep tumor therapy.** b** PVA-agarose hydrogel reinforced with HBPL/TA demonstrates antibacterial, antioxidant, and scar-free wound healing properties, with robust mechanical performance. **c** Polyaspartate hydrogel stabilizes ACP via Ca.^2^⁺ chelation, forming a mechanically robust mineralizable scaffold that supports bioactive integration for bone regeneration. Reproduced with permission from [235, 237, 239]

Moreover, mechanically extreme hydrogels demonstrate substantial promise in advancing cancer therapies particularly in overcoming the limitations of traditional treatments such as high-dose systemic chemotherapy and multidrug resistance. Similarly, Zhang et al. [235] designed a hydrogel platform incorporating aggregation-induced emission (AIE) photosensitizers, which undergo deformation upon near-infrared (NIR) laser activation (Fig. 15a). This deformation enhances rapid, localized drug release through photothermal heating, significantly improving delivery efficiency to deep-seated tumor tissues such as those in breast cancer. These approaches illustrate how the integration of mechanical toughness and functional responsiveness can transform hydrogels into potent platforms for intelligent cancer therapeutics.

In conclusion, the integration of mechanically extreme hydrogels into drug delivery platforms has demonstrated remarkable promise in advancing precision medicine by enabling controlled, localized, and sustained therapeutic release under physiologically demanding conditions. Their superior mechanical resilience, resistance to deformation, and capacity to maintain functional integrity under stress allow these hydrogels to operate effectively in dynamic and often harsh biological environments. Across a wide spectrum of applications including localized therapy, extended-release formulations, ophthalmic treatments, and tumor-targeted delivery, these hydrogels exhibit enhanced therapeutic efficacy, reduced systemic toxicity, and improved patient compliance.

#### Wound Healing

Skin wounds, particularly chronic and large-area injuries, require materials that can adapt to complex healing dynamics while withstanding mechanical perturbations from movement, swelling, or external contact. Hydrogels with extreme mechanical properties, defined by high tensile strength, toughness, and fatigue resistance, can act as dynamic scaffolds that remain functional throughout the healing process, even in mechanically challenging environments [236]. These materials not only protect against microbial invasion and desiccation but also act as bioactive substrates to stimulate angiogenesis and dermal regeneration under biomechanical strain. With the growing demand for wound care solutions in mobile, wearable, and extreme environments (e.g., in battlefield medicine, space exploration, or wearable healthcare), mechanically extreme hydrogels represent a transformative material class. Their ability to couple robust mechanical performance with bioactivity positions them as ideal candidates for high-performance wound management technologies. For example, Liu et al. [237] developed a strong and tough hydrogel dressing based on a double-network of PVA and agarose, reinforced by hyperbranched polylysine and tannic acid (Fig. 15b). This hybrid system not only demonstrated potent antimicrobial and antioxidant functions but also promoted rapid healing in infected wounds while reducing scar formation. Inspired by bamboo, Zhang et al. [238] developed hollow hydrogel fiber dressings. Wall-thickness tuning enables controlled drug release, while H-bond/Ca^2^⁺ crosslinking enhances mechanical strength, allowing functionality under daily mechanical stress.

Mechanically extreme hydrogels advance wound healing by integrating robust mechanical properties with smart-responsive functions such as on‑demand drug release, real‑time monitoring, and antimicrobial action. This mechano‑functional synergy enhances healing precision and safety while extending applicability to complex clinical scenarios beyond the reach of conventional dressings.

#### Hydrogel Scaffolds

Mechanically extreme hydrogels, when employed as therapeutic scaffolds, offer critical advantages in the regeneration of load-bearing and structurally sensitive tissues such as articular cartilage, skeletal muscle, organs, and bone [231].For instance, amorphous calcium phosphate (ACP), a key precursor in bone mineralization, plays a central role in guiding bottom-up collagen mineralization. Zhang et al. [239] successfully stabilized ACP within a polyacrylamidoaspartic acid (PASP) hydrogel network containing repeating dicarboxylate units (Fig. 15c). The hydrogel matrix chelates calcium ions and forms hydrogen bonds with ACP, delaying its crystallization and enabling controlled mineral deposition. The hydrogel achieved a compressive strength of 1.2 MPa, significantly surpassing that of conventional scaffolds and preventing collapse under physiological loading. Siebert et al. [240] developed a 3D-printed hydrogel scaffold embedded with light-activated ZnO microparticles functionalized with vascular endothelial growth factor (VEGF), combining antibacterial functionality, angiogenesis promotion, and moisture retention within a single matrix. The combination of extreme mechanical performance with biological function modulation makes hydrogel scaffolds a cornerstone in next-generation tissue engineering platforms.

#### Bioelectronics

In addition to its wide range of applications in tissue engineering and biomedical materials, hydrogel has also shown great potential in the field of flexible bioelectronics in recent years. In particular, in self-powered and implantable patch systems, hydrogels are widely used as key functional units due to their excellent flexibility, conductivity, and biocompatibility [14, 241].

**Fig. 16:**
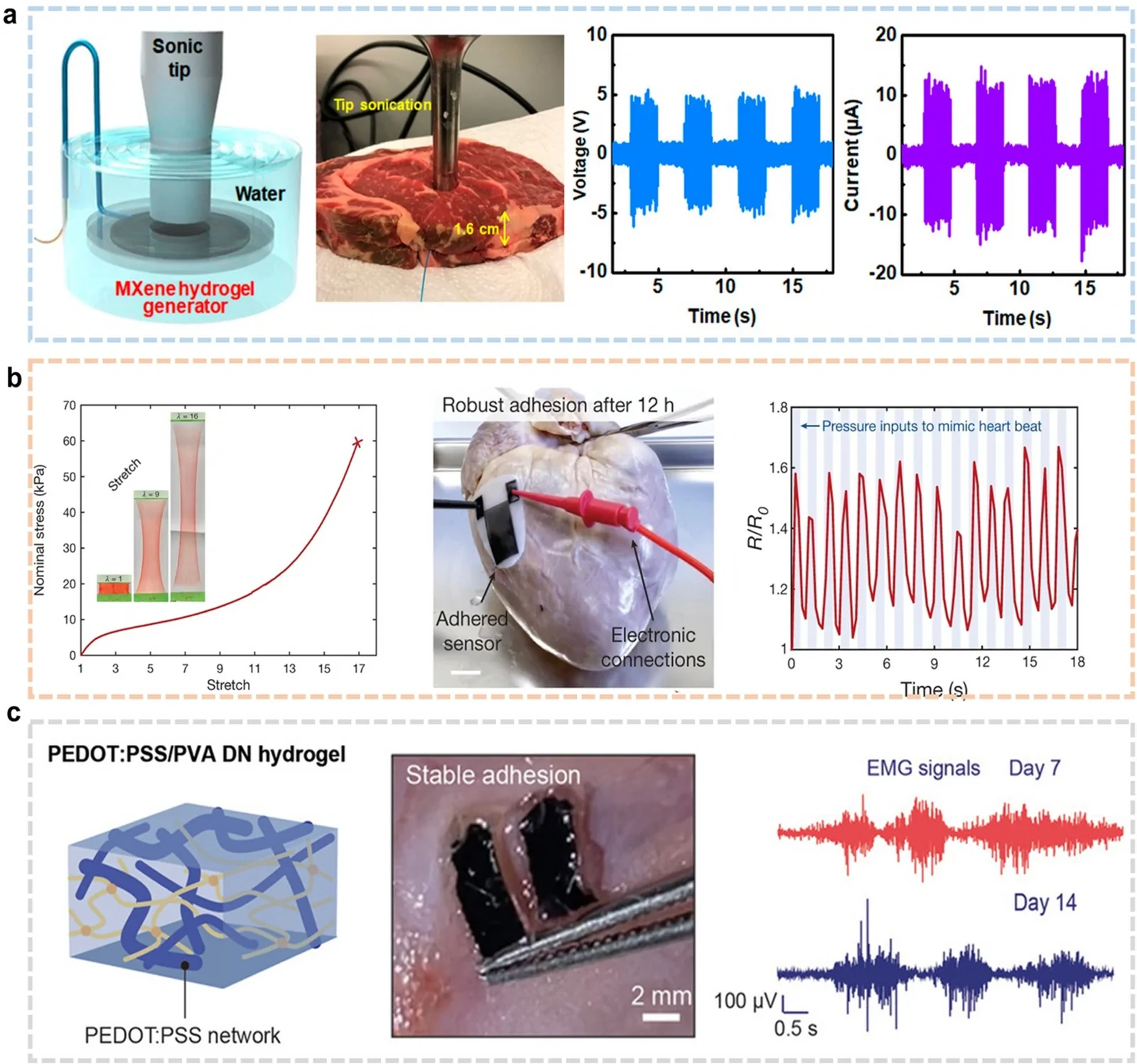
Mechanical behavior of mechanically extreme hydrogels and its applications in implantable bioelectronics. **a** T An M-gel generator encapsulated in beef tissue with voltage/current measurements at 10 MΩ and 100 kΩ loads. **b** Adhesion performance of a DST–strain-sensor hybrid on a beating ex vivo porcine heart: Photographs and nominal stress–stretch relationship. **c** EMG recording via implanted conducting hydrogel electrodes on muscle at days 7 and 14 post-implantations. Reproduced with permission from [242, 245, 246]

In terms of biological self-powered devices, hydrogels can be used to construct triboelectric effect-based nanoelectric generators (TENGs) for energy harvesting without external power supply. Excellent interfacial connectivity performance is crucial for the stable operation of TENG devices. For example, Wang et al. [242] enhanced the interfacial bonding between hydrophilic hydrogel (electrode layer) and hydrophobic polydimethylsiloxane (PDMS, electrified layer) by introducing phenylphenyl ketone molecules on the hydrogel surface, thereby constructing a flexible tension power generation device that can efficiently harvest energy and light up to 20 LEDs by pressing, bending, stretching, and other mechanical means. On the other hand, MXene nanosheets were introduced to significantly enhance the conductivity and mechanical properties of hydrogels. In 2020, Lee et al. [243] developed a tissue-mimicking implantable hair generator based on MXene-poly(vinyl alcohol) (PVA) hydrogel, which can respond to a wide range of common clinical and home ultrasound sources, achieve stable electrical output, and show promising applications in imaging and physical therapy (Fig. 16a).

Unlike noninvasive devices, implantable hydrogel devices need to be in direct contact with biological tissues for a long period of time, thus putting higher requirements on the biocompatibility and mechanical matching of materials [244]. Zhao et al. [245] designed a hydrogel tape that is resistant to shedding and mechanical damage, combining a biopolymer with polyacrylic acid grafted with NHS ester groups to achieve high toughness through a double-network structure, which can be stretched up to 16 times of the original length and can crosslink with tissues in situ after the interface has absorbed water, which significantly enhances the adhesion properties (Fig. 16b). In terms of electrode materials, Guo et al. [246] constructed PEDOT: PSS/PVA double-network hydrogel electrodes with high conductivity (~ 10 S cm^−1^) and high stretchability (150%) for implantable electromyography (EMG) signal acquisition by introducing PVA into the PEDOT/PSS system (Fig. 16c). Further, the adhesive layer formed by surface photopolymerization achieves a robust bond with the tissue (interfacial toughness of 600 J m^−2^), and high signal-to-noise signal acquisition with low-voltage neurostimulation has been successfully achieved in animal models. Bioelectronic systems based on hydrogel materials are breaking through the key bottlenecks of mechanical properties, biocompatibility and interfacial integration, and are showing broad development prospects in the fields of flexible electronics and implantable medical devices.

### Bioinspired Actuation and Robotic Applications

#### Artificial Muscle

Mechanically extreme hydrogels represent a new class of materials ideally suited for artificial muscle systems, offering a rare combination of high toughness, resilience, and rapid response to stimuli. These hydrogels can undergo reversible, large-amplitude deformations in response to external triggers such as electric fields, temperature shifts, pH changes, and light irradiation, closely mimicking the contraction-extension dynamics of natural muscle tissues. For instance, hydrogels engineered with electroactive polymers or photothermal nanomaterials can convert electrical or optical energy into mechanical work, delivering programmable deformation suitable for soft robotic grippers, biomimetic limbs, or adaptive wearable devices. As research in this area progresses, the development of hydrogels capable of sustaining large mechanical loads under extreme environmental conditions will continue to expand their utility in advanced actuation platforms. These hydrogels not only bridge the gap between biological function and synthetic design but also enable next-generation bioinspired motion systems that are lightweight, compliant, and highly functional.

**Fig. 17:**
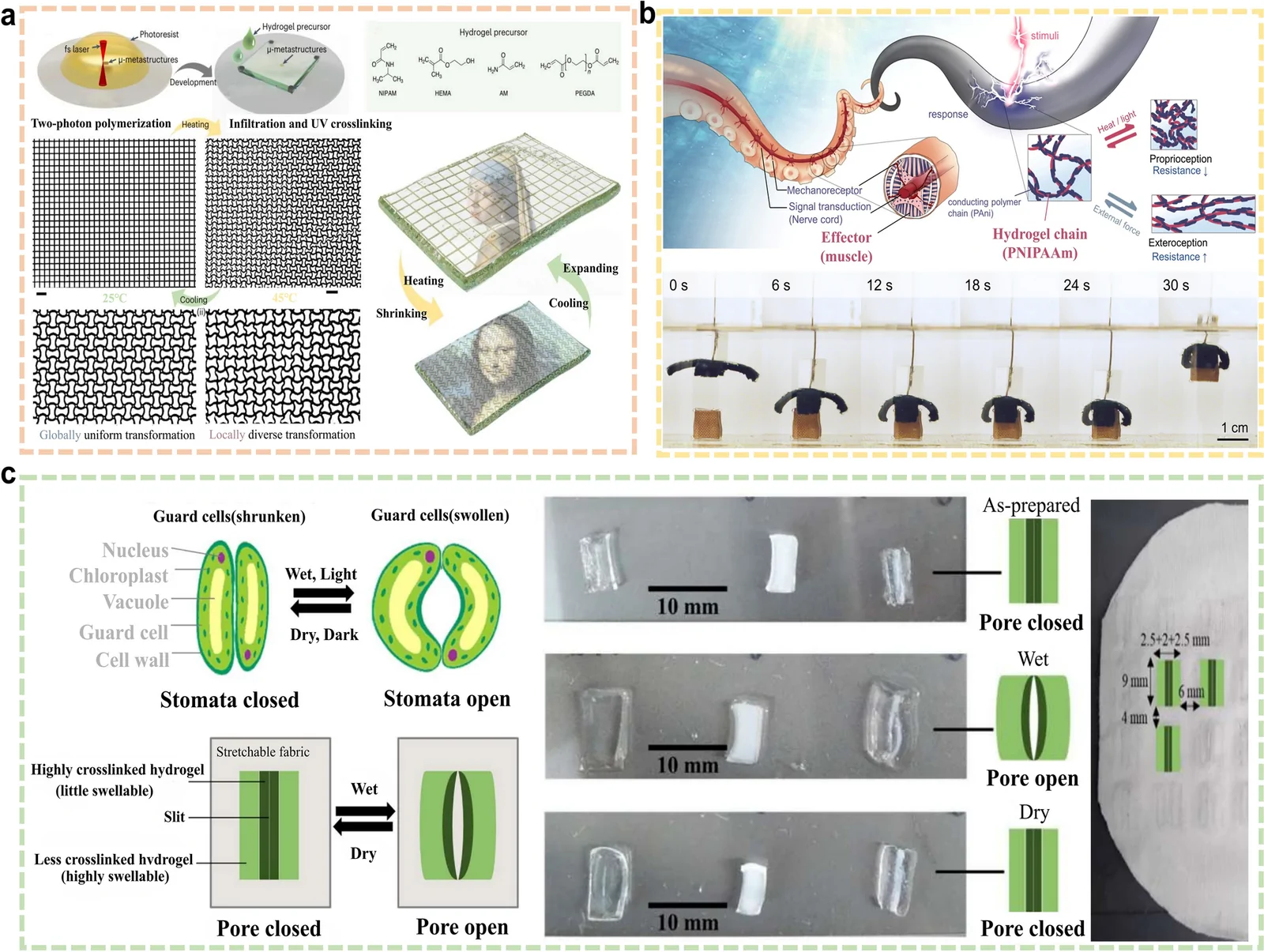
Bioinspired actuation and robotic applications of mechanically extreme hydrogels. **a** Two-photon printed microstructures in thermoresponsive hydrogels enable submicron reversible actuation for artificial muscles. **b** NIR-responsive conductive hydrogel actuators enable directional grasping and ultrafast expansion, offering robust biomimetic actuation for soft robotics. **c** Humidity-responsive smart textiles with durable hydrogel networks dynamically adjust moisture absorption and porosity. Reproduced with permission from [247, 249, 253]

Recent advancements in hydrogel-based actuators have demonstrated remarkable progress in bridging the performance gap between synthetic materials and biological muscles. For example, the research team led by Sitti [247], proposed a micro-superstructure conformational transformation strategy based on linearly responsive hydrogel artificial muscles (Fig. 17a).

To enhance actuation efficiency and long-term operability under ambient conditions, Jiang et al. [248] incorporated hydrophilic poly(ethylene glycol) (PEG) segments into a liquid crystal-crosslinked network. The introduction of just 10 mol% PEG enabled the material to achieve a solvent uptake of 78 wt%, reducing the phase transition temperature of the liquid crystalline domains and slowing solvent evaporation. This approach preserved actuation performance even in dry environments, with the resulting polymer–organogels achieving a high deformation amplitude (66%), rapid deformation rate (275% s^−1^), and power density (438 kW m^−3^). Notably, the Young’s modulus (~ 133 kPa) of these hydrogels approaches that of human skin, making them well suited for integration in soft wearable systems. These breakthroughs collectively underscore the capacity of mechanically extreme hydrogels to match and potentially exceed the functional performance of natural skeletal muscle, offering powerful platforms for next-generation soft robotics, bioinspired machinery, and wearable exoskeletal systems.

#### Soft Robotics

Mechanically extreme hydrogels have emerged as foundational materials in the development of next-generation soft robotics, offering a unique combination of compliance, toughness, and stimuli responsiveness. For instance, hydrogel actuators can mimic biological musculature by dynamically bending, twisting, or elongating in response to localized stimulation, making them suitable for multi-arm manipulators, bioinspired grippers, or autonomous locomotion units. As the field progresses, incorporating hydrogels with multimodal responsiveness and integrated sensing capabilities will allow soft robots to transition from passive deformation to intelligent and interactive systems, pushing the boundaries of what soft machines can achieve in extreme mechanical and physiological conditions.

He et al. [249] developed a flexible tactile actuator based on a conductive polymer double-network hydrogel, specifically engineered to mimic the extension and contraction of octopus tentacles through closed-loop feedback control (Fig. 17b). The actuator exhibits reliable object sensing and gripping functionality, driven by asymmetric shape transformation triggered via near-infrared (NIR) light irradiation. The incident NIR light creates a temperature gradient across the hydrogel, inducing directional bending that enables precise grasping motions. Beyond this, recent research has shown that cellulose nanofibers organized into an anisotropic network form a reinforcing scaffold that dramatically improves tensile properties and directional actuation [250]. In such designs, hydrogels are capable of ultrafast expansion rates (100–150% s^−1^) and maximum expansion ratios up to 250-fold, far exceeding those of conventional polymeric gels. Moreover, the incorporation of strong hydrogels significantly enhances the precision, robustness, and environmental adaptability of soft robots [251]. Responsive to light, heat, and magnetic fields, these hydrogels are task-programmable for stable operation in complex settings. Their mechanical reliability in gripping biological tissues, minimally invasive surgery, and high-speed precision operations establishes them as a core material for next-generation robotic systems.

#### Smart Switch

Integrating strong, tough hydrogels into smart switching systems significantly enhances response speed, operational durability, and structural stability. With high fatigue resistance, programmable deformation, and stimuli-responsive actuation, these hydrogels are well suited for frequent cycling under varying environmental conditions. The key lies in balancing strength and toughness to ensure long-term cyclic reliability, maintaining precise and efficient switching performance over extended service life. For example, Xie et al. [7] enhanced the thermal stability of thermochromic hydrogels by optimizing glycerol content. This adjustment improved the hydrogel’s ability to maintain electrical conductivity and chromic behavior under extreme temperature variations, allowing the smart switch to generate a stable current despite thermal cycling. The resulting smart window platform demonstrated improved responsiveness and reliability, crucial for energy-efficient climate regulation in building environments [252].

In the expanding field of smart textiles, strong and tough hydrogels play a pivotal role in achieving responsive, durable, and mechanically robust fabric-based systems. For instance, Lao et al. [253] developed smart fabrics incorporating humidity-responsive hydrogels, which dynamically regulate water absorption and pore opening based on environmental humidity levels (Fig. 17c). These textiles rely on the swelling–deswelling behavior of the hydrogel to adaptively alter fabric permeability. The incorporation of mechanically strong hydrogel networks ensures that these smart fabrics maintain structural integrity and functional consistency, even after repeated washing, stretching, and daily use.

Beyond textiles, the integration of strong hydrogels into fluidic regulation systems, such as smart hydrogel water valves, further illustrates their multifunctional adaptability. These systems exploit the stimuli-responsive volume changes of hydrogels to autonomously control water flow based on thermal or chemical triggers, thereby enabling the development of self-regulating, programmable valve platforms. Notably, researchers have engineered a 2D hydrogel by combining hydroxypropyl cellulose (HPC) with graphene oxide (GO), achieving a high-performance material that simultaneously exhibits rapid swelling kinetics, electrothermal actuation, and superior mechanical resilience [254]. The resulting hydrogel demonstrates a Young’s modulus of up to 2.5 GPa, a significant milestone for polymer-based soft materials.

### Smart Sensing and Security Applications

#### Intelligent Monitoring

Strong and tough hydrogels are emerging as critical enablers in the next generation of intelligent sensing technologies, particularly in applications requiring high resilience, adaptability, and precision such as health monitoring, injury detection, motion tracking, and disease diagnostics [255]. Hydrogel-based sensors operate by transducing external mechanical deformations (such as stretching, pressure, or strain) into measurable electrical, chemical, or optical signals. Their programmable responsiveness to environmental cues allows them to dynamically adjust characteristics such as shape, conductivity, and mechanical stiffness, providing highly accurate, flexible, and real-time monitoring in both wearable and implantable platforms.

**Fig. 18:**
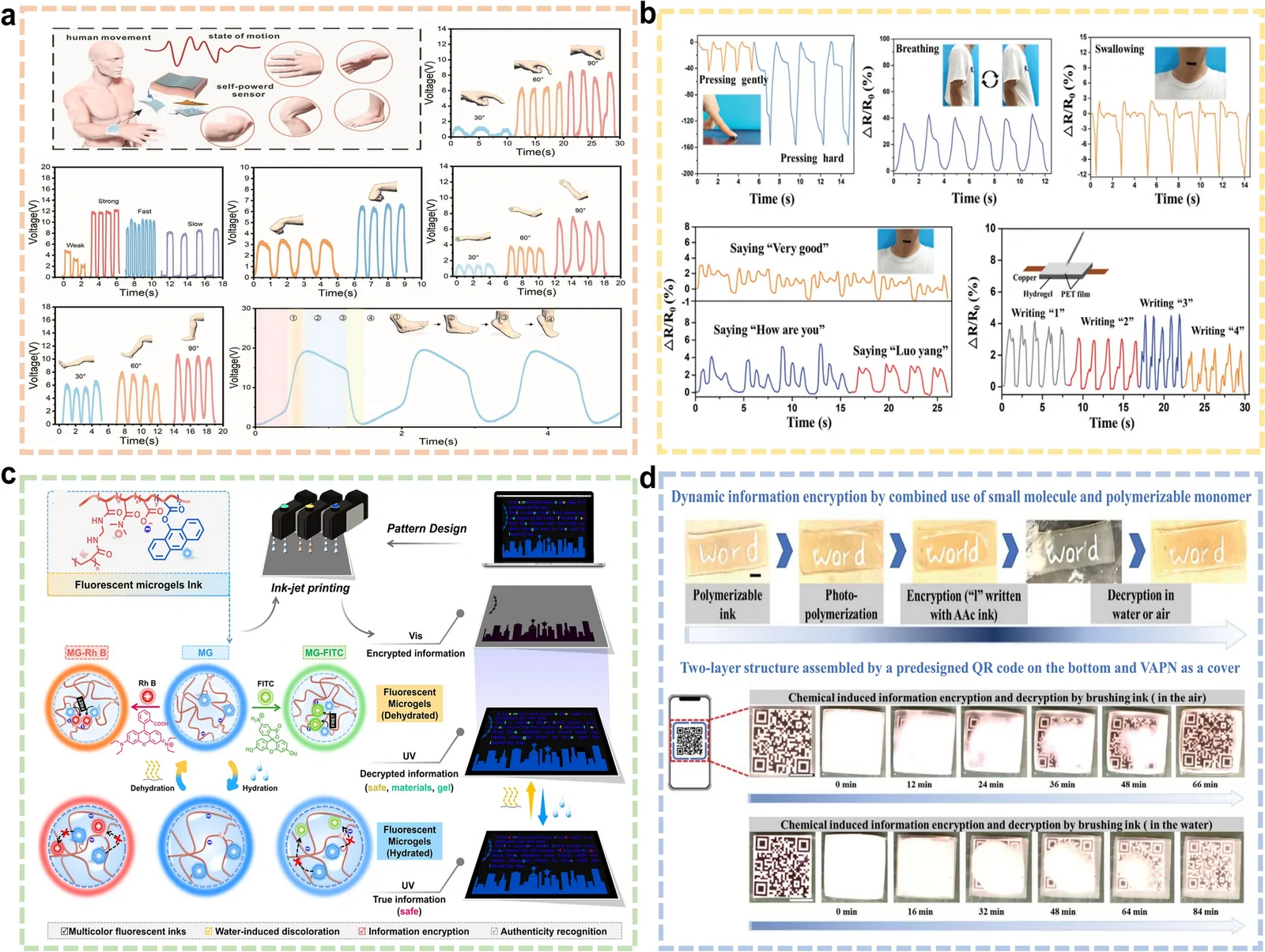
Smart sensing and security applications of mechanically extreme hydrogels. **a** Ion-hybridized hierarchical ionogel sensor shows ultrafast response and robust cyclability. **b** THMA/ILs-enhanced double-network hydrogel exhibits both high strength, ultrahigh extensibility, and wide-range sensing capabilities, enabling precise monitoring of pulse and speech. **c** Stimuli-responsive multicolor fluorescent hydrogel inks for advanced information encryption. **d** Chemically triggered phase separation encryption strategy based on PNVCL hydrogels achieves dual-mode (dynamic/permanent) encryption by disrupting local interactions through carboxyl-group inks to induce phase transition. Reproduced with permission from [87, 259–261]

One particularly impactful application is in biophysical signal detection, where strong and tough hydrogels are used to monitor arterial pulse waves, a vital biomarker for assessing blood pressure, vascular health, and cardiac function [256]. For example, Peng et al. [87] developed a high-performance ion gel featuring a layered mesh structure reinforced by ion hybridization, designed specifically for flexible strain sensing applications (Fig. 18a). This ion gel sensor not only achieved a fast-response time of 36 ms but also demonstrated remarkable durability, maintaining consistent signal output after over 4000 mechanical cycles. Furthermore, its thermal resilience allowed stable performance under extreme temperature conditions, ranging from subzero environments to as high as 200 °C, exhibiting great adaptability for harsh or biomedical environments.

As the demand for early-stage diagnosis and chronic disease management intensifies, the ability to continuously and noninvasively monitor non-cognitive biomarkers becomes increasingly vital. Traditional diagnostic techniques are often invasive, inconvenient, or unsuitable for long-term use, particularly in home healthcare or wearable applications [257, 258]. Wang et al. [259] developed a highly elastic and conductive hydrogel sensor by copolymerizing PAM with tris(hydroxymethyl)methyl acrylamide (THMA) and cationic imidazolium-based ionic liquids (ILs), all embedded in a core–shell conductive matrix of PEDOT:PSS (Fig. 18b). The resulting hydrogel exhibited a tensile strength of 0.25 MPa, an exceptional fracture strain of 1015%, and robust resilience and fatigue resistance. It maintained high-fidelity signal detection across a broad strain range (1–900%) and pressure sensitivity from 0.05 to 100 kPa, enabling precise detection of minute physiological movements, such as pulse fluctuations and joint flexion.

Strong and tough hydrogels are ideal for high-fidelity monitoring due to their mechanical properties that enable seamless adaptation to the complex dynamics of the human body. Through strategies like hierarchical crosslinking and supramolecular assembly, they achieve both high mechanical integrity and adaptive responsiveness. This makes them central to next-generation sensor applications such as real-time health monitoring and noninvasive diagnostics, driving the evolution of wearable, intelligent biomedical systems.

#### Information Encryption and Anti-Counterfeiting

The advent of mechanically extreme hydrogels, which combine structural robustness with dynamic responsiveness, has opened new frontiers in the fields of information encryption, identity verification, and anti-counterfeiting [6]. Shang et al. [260] developed a printable multicolor fluorescent microgel system with rapid water responsiveness by copolymerizing *N*-isopropylacrylamide (NIPAM), acrylic acid (AAc), and anthracene derivatives (Fig. 18c). These microgels exhibit programmable fluorescence behavior based on Förster Resonance Energy Transfer (FRET) interactions between anthracene moieties and cationic fluorescent dyes. In the dry state, distinct multicolor fluorescence is observed. Upon exposure to water, swelling disrupts the FRET pathway by diluting Rhodamine B aggregates, resulting in a sharp shift in emission color.

Further advancing the field, Hou et al. [261] developed a chemical-triggered phase separation encryption strategy based on mechanically reinforced poly(*N*-vinylcaprolactam) (PNVCL) hydrogels (Fig. 18d). By applying chemical inks containing carboxyl groups such as citric acid or polyacrylic acid, the team disrupted local hydrogen bonding, inducing phase separation and creating opaque regions that served to conceal information. Their method offered two main encryption modes: (1) dynamic encryption, wherein small-molecule inks produce self-erasing messages via diffusion-mediated re-equilibration; (2) permanent encryption, using polymer inks or photopolymerizable monomers to form durable, phase-separated structures for long-term information storage.

Leveraging multi-stimulus responsiveness and mechanical robustness, strong and tough hydrogels have become key materials for next-generation encryption and anti-counterfeiting. They enable reversible, condition-dependent information display/concealment and offer tamper-evident, durable performance, making them suitable for high-security applications such as financial instruments, legal documents, and pharmaceutical packaging, thereby driving the evolution of intelligent security platforms.

## Conclusion and Future Perspectives

### Conclusion

Conventional hydrogels, due to their sparse polymer networks and high-water content, often suffer from poor mechanical strength, limiting their performance under demanding application conditions. As a result, the development of mechanically extreme hydrogels (materials that combine exceptional toughness, strength, and resilience) has become a core objective in hydrogel research. These Adv. Mater. not only overcome traditional mechanical limitations but also retain essential features such as high biocompatibility, tunable responsiveness, and environmental adaptability.

Looking ahead, the future development of tough and intelligent hydrogels will be fundamentally guided by the scenario-adaptive design philosophy established in Sect. 4.1. This will shift the focus toward multiscale structural design informed by operational demands, bioinspired dynamic adaptability to environmental fluctuations, and the creation of energy-efficient material systems that maintain function under specific extremes. Innovations in raw material chemistry, composite interfaces, and multistimuli responsiveness will continue to be driven by the need to meet these scenario-specific performance profiles. Furthermore, the integration of artificial intelligence, machine learning, and computational modeling into hydrogel design pipelines may accelerate the discovery of novel formulations with predictive and adaptive mechanical behaviors tailored to complex, real-world environments. In summary, the field of mechanically extreme hydrogels is transitioning from foundational research to real-world deployment across healthcare, soft robotics, wearable electronics, and environmental technologies. As material science, biomedical engineering, and soft matter physics converge, strong and tough hydrogels are poised to serve as a cornerstone in the development of next-generation smart systems that demand simultaneous mechanical robustness and intelligent functionality.

This review systematically presents the theoretical frameworks and preparation strategies that underpin the development of mechanically extreme hydrogels, emphasizing their unique capabilities in sustaining high-strength performance under diverse mechanical loads such as stretching, compression, twisting, and knotting. By analyzing various reinforcement mechanisms from network architecture and crosslinking chemistry to multiscale structural design, this review highlights the critical principles guiding the synthesis of tough hydrogels and the translation of these materials into a broad range of high-performance applications. These include biomedicine, wearable sensors, soft robotics, and smart control systems where materials are required to maintain structural integrity and functionality under complex dynamic conditions.

### Perspective

In the next decade, the study of ultra-mechanical hydrogels may transition from a traditional material-centric design paradigm to a function-driven, scenario-aware, and intelligent feedback model. This evolution, building upon the framework of Sect. 4.1, hinges on moving beyond optimizing static mechanical properties and toward achieving real-time environmental adaptation, multifunctional responsiveness, and long-term durability within defined operational envelopes. For instance, integrating scenario-specific adaptive mechanics with intelligent feedback loops could elevate hydrogels from passive structural materials to active components capable of perception, decision-making, and actuation within their target environment.

#### Challenges and Opportunities in Multi-scale Design

The future of ultra-mechanical hydrogels hinges on moving beyond optimizing static mechanical properties and toward achieving real-time environmental adaptation, multifunctional responsiveness, and long-term durability within defined operational envelopes. This requires coherent integration across scales—from molecular design to macroscopic performance. For instance, at the molecular level, precise control of dynamic bond kinetics is essential. On the microstructural scale, orchestrating phase separation or nanoparticle alignment to simultaneously achieve high toughness, rapid stimulus response, and functional properties remains a key challenge. Bridging these scales to ensure robust and reproducible macroscopic performance is a fundamental hurdle for scalable manufacturing.

#### New Frontiers in Intelligent Integration

Machine learning algorithms can accelerate high-throughput screening and optimization of hydrogel formulations for target scenarios by leveraging predictive modeling and structure–property relationship under specific environmental conditions. For instance, inverse design approaches allow researchers to start with a desired set of mechanical properties (e.g., a specific toughness and swelling ratio) and use AI to identify feasible polymer compositions and crosslinking strategies that meet those targets. Furthermore, active learning frameworks can iteratively guide experimental synthesis by prioritizing the most informative compositions to test next, dramatically reducing the number of required trials. For example, AI-driven simulations can predict hydrogel behavior under diverse environmental conditions, reducing experimental trial-and-error costs and enabling customized design of ultra-mechanical hydrogels. These approaches move beyond simple prediction toward truly goal-oriented material discovery. However, challenges such as data quality, model interpretability, and large-scale manufacturing remain bottlenecks.

#### Pathways for Sustainability and Scalability

Translating laboratory breakthroughs into sustainable, real-world applications demands concurrent advances in biocompatibility, standardized manufacturing, and safety. Breakthroughs in biomedical applications are critical. To address toxicity and swelling issues during long-term implantation, future research will focus on developing more biocompatible ultra-mechanical hydrogels. For instance, molecular design can regulate crosslinked networks to achieve controlled swelling, preventing mechanical failure due to excessive expansion. Additionally, self-growth and self-strengthening mechanisms will enable hydrogels to repair their structures post-damage, thereby reducing potential toxic release risks. However, balancing biodegradability and immunocompatibility remains essential for personalized medicine, regenerative medicine, and other needs. Beyond material design, clinical translation faces tangible hurdles in standardization. A fundamental requirement for clinical translation is the precise control of crosslinking density across production batches to ensure identical swelling behavior and degradation rates. Furthermore, comprehensive safety profiles are required. This means not only testing for cytotoxicity but also assessing specific risks like in vivo hemolysis, complement activation, and the long-term inflammatory response to degradation fragments. For biomedical applications, breakthroughs depend on developing more biocompatible hydrogels and establishing rigorous standards for fabrication and safety evaluation. Furthermore, cross-disciplinary integration of technologies like 3D printing and bioorthogonal chemistry is crucial to drive industrialization. By converging these pathways, the next generation of hydrogels will not only be mechanically extreme but also manufacturable, safe, and sustainably integrated into advanced technologies.

## Abbreviations

AAc: Acrylic acid
AAm: Acrylamide
ACMO: Acryloylmorpholine
ACP: Calcium phosphate
AIE: Aggregation-induced luminescence
Alg: Alginate
AMPS: 2-Acrylamido-2-methyl-1-propanesulfonicacid
ANFs: Aramid nanofibers
CA: Carrageenan
CAD: Computer-aided design
Ce-MOFs: Cerium-based metal–organic skeletons
CIJ: Continuous inkjet printing
CMCT: Carboxymethyl chitosan
CNCs: Cellulose nanocrystals
CNS: Inorganic(clay) network structure
CNTs: Carbon nanotubes
CS: Chitosan
DIW: Direct ink writing
DLP: Digital light processing
DMAEA-Q: Methyl chloride quaternized *N*,*N*-dimethylamino ethylacrylate
DMD: Digital micromirror device
DMSO: Dimethyl sulfoxide
DMx: Quaternary ammonium ionic liquids
DNA: Deoxynucleic acid
DOD: Drop-on-demand inkjet printing
dPEDOT: Dopamine methacrylate-hybridized poly(3,4-ethylenedioxythiophene) nanoparticles
dsDNA: Double-stranded DNA
ECM: Extracellular matrix
EMIES: 1-Ethyl-3-methylimidazole ethyl sulfate
F-127: Pluronic F-127
f-BNNS: Functionalized-boron nitride nanosheets
fc-DN: Fibrillar connected double networks
FDA-SNF: Freeze-dried and annealed-flexible SiO_2_ nanofibers
Fe_3_O_4_@CNCs: Fe_3_O_4_-modified cellulose nanocrystals
g-C_3_N_4_: Graphitic carbon nitride
GO: Graphene oxide
GQDs: Graphene quantum dots
HA: Hyaluronic acid
HAPAAm: Hydrophobically associated polyacrylamide
HBPL: Hyperbranched polylysine
HIVE: Hive-structured assemblable bespoke scaffold
HPC: Hydroxypropyl cellulose
HP-*α*-CD: Hydroxypropyl-*α*-cyclodextrin
LAP: Laser-assisted printing
LCST: Lower critical solution temperature
Lig–Ag NPs: Lignin–silver hybrid nanoparticles
MAAc: Methacrylic acid
MAAm: Methacrylamide
MACNF: *β*-Chitosan nanofiber
MMT: Montmorillonite
MOF (V-ZIF-8): Metal–organic framework (vinyl-functional zeolitic imidazole framework-8)
mPEG: Poly(ethylene glycol)
mPEG 480-acrylate: Poly(ethylene glycol) 480 methyl ether acrylate
MPS-CS: 3-(Trimethoxysilyl) propyl methacrylate (MPS)-functionalized upconversion (UC)fluorescent NaREF4: Ln^3+^@NaYF_4_ core−shell nanoparticles
NaSS: Sodium *p*-styrenesulfonate
NIR: Infrared
NPs: Nanoparticles
P(AAm-co-AAc): Poly(acrylamide-co-acrylic acid)
P(AAm-co-MAH): Poly(acrylamide-co-methacryloyl-hydrazide
PAAc: Polyacrylic acid
PAM/PAAm: Polydopamin–polyacrylamid
PAMPS: Ploy(2-acrylamido-2-methyl-1-propanesulfonic acid)
PANI: Polyaniline
PAPBA: Poly(3-acrylamidophenylboronic acid)
pBP nanosheets: Polydopamine-modified black phosphorus nanosheets
PCL: Polycaprolactone
PDA: Polydopamine
PDMAEA-Q: Poly(2-(dimethylamino)ethylacrylate) methyl chloride quaternary salt
PDMS: Polydimethylsiloxane
PEG: Polyethylene glycol
PEG: Polyethylene glycol
PEGDA: Poly(ethylene glycol) diacrylate
PEGDA: Polyethylene glycol diacrylate
PHEAA: Poly(*N*-methylol acrylamide)
pKa: Acid dissociation constant
PMAA: Poly(methylacrylic acid)
PMAD: Copolymerization of acrylamide (AM), acrylic acid (AA), and [2-(methacryloyloxy)ethyl] trimethyl ammonium chloride solution (DMC)
PNIPAAm: Poly(*N*-isopropylacrylamide)
PNMA: Poly(*N*-methylol acrylamide)
POx: Poly(2-oxazoline)
PR: Polyrotaxane
PSBMA: Poly(sulfobetaine methacrylate)
PTHF: Polytetrahydrofuran
PVA: Polyvinyl alcohol
PVA-PI: Poly(vinyl alcohol)-poly(imide)
QACNF: Quaternized cellulose nanofibers
rGO: Reduced graphene oxide
ROS: Reactive oxygen species
SA: Sodium alginate
SiNPs: Silicon nanoparticles
SLA: Stereolithography
SMA: Stearyl methacrylate
ssDNA: Single-stranded DNA
TA: Tannic acid
TOCNF: TEMPO-oxidized cellulose nanofibers
TPP: Two-photon polymerization
TPU: Thermoplastic polyurethane
UCST: Upper critical solution temperature
UV: Ultraviolet
XLG: LAPONITE’s XLG nanosheet
*β*-CD-TCNCs: *β*-Cyclodextrin-modified bundled cellulose nanocrystals
*κ*-Car: *κ*-Carrageenan
